# New Universal Rules of Eukaryotic Translation Initiation Fidelity

**DOI:** 10.1371/journal.pcbi.1003136

**Published:** 2013-07-11

**Authors:** Hadas Zur, Tamir Tuller

**Affiliations:** 1 The Blavatnik School of Computer Science, Tel Aviv University, Tel-Aviv, Israel; 2 Department of Biomedical Engineering, The Engineering Faculty, Tel Aviv University, Tel-Aviv, Israel; 3 The Sagol School of Neuroscience, Tel Aviv University, Tel-Aviv, Israel; Rutgers University, United States of America

## Abstract

The accepted model of eukaryotic translation initiation begins with the scanning of the transcript by the pre-initiation complex from the 5′end until an ATG codon with a specific nucleotide (nt) context surrounding it is recognized (Kozak rule). According to this model, ATG codons upstream to the beginning of the ORF should affect translation. We perform for the first time, a genome-wide statistical analysis, uncovering a new, more comprehensive and quantitative, set of initiation rules for improving the cost of translation and its efficiency. Analyzing dozens of eukaryotic genomes, we find that in all frames there is a universal trend of selection for low numbers of ATG codons; specifically, 16–27 codons upstream, but also 5–11 codons *downstream* of the START ATG, include less ATG codons than expected. We further suggest that there is selection for *anti optimal* ATG contexts in the vicinity of the START ATG. Thus, the efficiency and fidelity of translation initiation is encoded in the 5′UTR as required by the scanning model, but also at the beginning of the ORF.

The observed nt patterns suggest that in all the analyzed organisms the pre-initiation complex often misses the START ATG of the ORF, and may start translation from an alternative initiation start-site. Thus, to prevent the translation of undesired proteins, there is selection for nucleotide sequences with low affinity to the pre-initiation complex near the beginning of the ORF. With the new suggested rules we were able to obtain a twice higher correlation with ribosomal density and protein levels in comparison to the Kozak rule alone (*e.g.* for protein levels r = 0.7 vs. r = 0.31; p<10^−12^).

## Author Summary

Gene translation is an important step of the intra-cellular protein synthesis, which is a central process in all living organisms. Thus, understanding how translation efficiency is encoded in transcripts has ramifications to every biomedical discipline. The aim of the current study is to decipher the way translation initiation fidelity is encoded in eukaryotic transcripts, and how evolution shapes the beginning of transcripts. Based on the genomes of dozens of organisms we were able to derive a new, more precise, set of rules related to this process, facilitating a high resolution view of the mechanisms aiding translation initiation fidelity. Among others, we show that there is a universal trend of selection for low numbers of ATG codons upstream, but also in the 5–11 codons *downstream* of the START ATG, presumably to prevent translation of alternative ORFs over the main one. With the new suggested rules we were able to obtain a twice higher correlation with ribosomal density and protein levels in comparison to the previous translation initiation efficiency rule.

## Introduction

Gene translation is the central cellular process of sequence decoding to produce a protein. This process occurs in every organism and consumes most of the cellular energy [1]–[3], thus it has important ramifications to every biomedical field [3]–[9]. Translation consists of three stages: initiation (the binding of the ribosome to the transcript and the association of the small and large subunits), elongation (the iterative translation of triplets of nucleotides to amino acids by the ribosome) and termination (the disassociation of the large and small subunits of the ribosome and the completion of the process), which form a recurring cycle of events.

In eukaryotes the initiation step usually involves formation of a pre-initiation complex (consisting of the small subunit, 43S or the 40S subunit, and initiation tRNA). According to the accepted scanning model [10]–[14], this complex accompanied by additional initiation factors scan the mRNA sequence starting from its 5′ end towards its 3′ end, until a start codon is recognized (usually an AUG that is identified by the initiation tRNA), which represents the beginning of the open reading frame (ORF). The recognition of the start codon triggers the association of the large subunit and the beginning of the elongation step [10]–[13]. However, ATG codons are expected to be present in all possible reading frames upstream and downstream the START of the ORF; how thus does the scanning pre-initiation complex recognize the start ATG?

Over 30 years ago Kozak suggested that a specific context (*i.e.* the nucleotides before and after a codon) surrounding the initiating ATG codon is required for its recognition by the pre-initiation complex; asserting that this context should appear only in the vicinity of the initiating (START) ATG codon of the ORF [10]. Nevertheless, several deviations from the aforementioned scanning model have been reported based on small scale experiments. For example, there are reported cases of leaky scanning where AUG codons with sub-optimal contexts are skipped and translation initiates at a downstream AUG [14], and there are cases of translation via internal ribosome entry site (IRES) and additional non-canonical mechanisms [15]–[17], but these have been reported to be relatively rare. In addition, though there is a relatively low number of ATG codons in the 5′UTR as expected by the scanning model [18], it was shown that there are many cases with non-optimal ATG context scores for the main ATG codon, and cases of ATG codons at the 5′UTR with relatively optimal context scores [19], [20], suggesting that the scanning model is an over-simplification of the reality.

Previous genomic studies [10]–[13], that were usually based on a relatively small number of transcripts and\or organisms, were aimed at understanding the nucleotide pattern surrounding the START ATG, with few considering alternative ATGs, and if so usually focusing on those in the 5′UTR and not the ORF. Specifically, amongst others, it was shown that there are a number of preferred nucleotide sequences surrounding the main translation initiation codon in eukaryotic genomes, which may vary among organisms [21], [22]; there is a relation between sequence features and context scores at the beginning of the ORF and alternative initiation START codons [23], [24]. In addition various papers studied alternative ORFs in the 5′UTR (uORFs) and their effect on translation regulation and re-initiation [17], [25]–[28], the effect of mRNA folding on translation and its initiation [17], [29]–[33], the relation between alternative ATGs near the beginning of the ORF and protein localization [34], and the length of the 5′UTRs [27], [35].

As mentioned, the fact that there are less AUGs at the 5′UTR [18], and the effect of uORFs on the main ORF downstream, have been previously suggested and are related to the scanning model [17], [25]–[28]. However, we are the first to quantify the 5′UTR (and ORF) region under selection for less ATGs, and the fraction of the protein levels variance that can be explained by these facts.

The aim of the current study is to infer new universal rules related to the way in which initiation efficiency and fidelity are encoded in transcripts, to quantify potential alternative translation initiation events, and discern the selection processes maintaining the integrity of translation initiation and the resultant protein product. To this end, we analyse large scale genomic data of dozens of eukaryotes. Based on this analysis we reformulate and refine the translation initiation rules, and improve the understanding of the biophysics of initiation and the evolution of transcripts. Specifically, we show for the first time that there is *selection* for less ATG codons downstream to the beginning of the ORF; we estimate the length of the region under such selection upstream (5′UTR) and downstream the beginning of the ORF; we report some additional sequence signals related to initiation fidelity such as anti-‘Kozak’ sequences surrounding ATG codons near the beginning of the ORF, and the appearance of stop codons close to them, *that are under selection*; and we are the first to quantify the partage of the protein levels and ribosomal density variance that can be explained by the different signals near the beginning of the ORF.

## Results

### The nucleotide context of the START ATG is not conserved among genes and is not strongly correlated with expression levels

**Figure 1:**
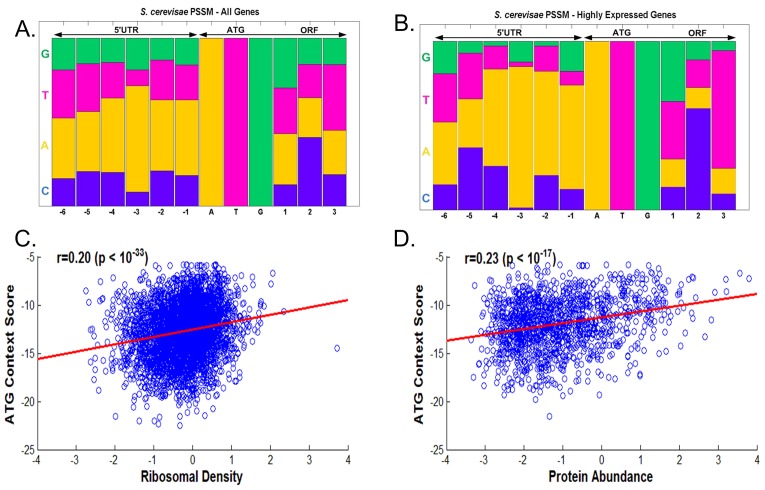
A–B. The position specific scoring matrix (PSSM) describing the distribution (*i.e.* relative frequency) of nucleotides near the main START ATG in *S. cerevisiae* for all genes (A.), and for genes with high ribosomal-load (B.). C.–D. The correlation between the ATG context score and protein levels and ribosomal densities in *S. cerevisiae*.

First, we re-studied the distribution of nucleotide allocation near the START ATG, focusing on *Saccharomyces cerevisiae*. As was reported in previous studies [21], we find that in most of the positions near the START ATG, the distribution of the nucleotide composition is relatively close to uniform in *S. cerevisiae* genes (Figure 1A; for example in position −1 the probability to see A/T/C/G is 0.45/0.21/0.18/0.16 respectively, and in position +1 the probability to see A/T/C/G is 0.3/0.27/0.13/0.3 respectively).

The meaning of the above result is that if we consider the nucleotide with the highest probability (*e.g.* A in positions ±1), there is a very high probability to see a different nt in this position (probability 0.55 and 0.7 respectively); even if we consider the two nt with highest probability (AT in position −1 and AG in position +1), there is still relatively high probability to see a nt different to these two (probability 0.34 and 0.4 respectively).

However, as previously reported [10], [36], [37], some of the positions (*e.g.* −3; *i.e.* 3 nt before the beginning of the ORF) exhibit a *relatively* non-uniform nucleotide distribution (Figure 1A–B, see also supplementary Figure S37 related to *Schizosaccharomyces pombe*).

Throughout the paper we use the measure, (ribosomal density)·(mRNA levels) (Methods), that we named *ribosomal-load* as a measure of the intensity of gene translation. This measure considers total ribosomal flux over the gene transcripts. As can be seen in Figure 1B, also in the case of highly translated genes with high ribosomal load, the nucleotide distribution near the beginning of the ORF is relatively uniform (Figure 1B; Methods).

Is it possible that the nt distribution near the START ATG is related to the gene translation rate? When we computed the correlation between the optimality of the context score (a score that is based on the comparison to the context of the most highly translated genes; details in the Methods section) and the protein levels (Figure 1C), we obtained relatively low but significant correlations; similar results were obtained when we correlated the context score with ribosomal density (Figure 1D; as we demonstrate later these correlations can be slightly improved when binning the data).

**Figure 2:**
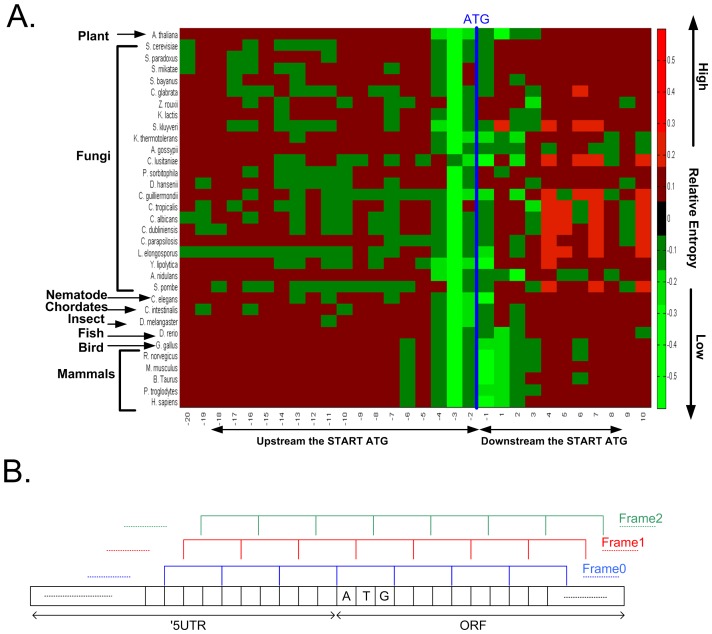
A. The relative entropy of the nucleotide distribution near the start ATG for 33 eukaryotes (see details in the Methods section), organized according to their evolutionary distance. High entropy denotes a non-conserved nucleotide distribution at a particular position. The most conserved position is −3 (three nucleotides upstream the START codon), but positions −2 and −1 are also relatively conserved. As can be seen different phylogenic groups exhibit different nucleotide conservation patterns. B. The definition of the three frames analyzed in this study.

Similar results were obtained in the 33 eukaryotes that were analyzed (Figure 2A). Specifically, we find that the relative entropy (Methods), a measure of conservation of a nucleotide among the organism's genes, of position −3 upstream of the START codon is the lowest; thus, nucleotide −3 is the most conserved site in all the analyzed organisms (Figure 2A), concordant with previous small scale studies [10], [36], [37]. However, as can be seen in Figure 2A, positions −1, −2, and 1 are also relatively conserved in many of the analyzed organisms, also in agreement with previous small scale studies [10], [36], [37]. Specifically, it seems that the patterns of conserved positions vary along the evolutionary tree. For example, position 1 is relatively conserved in all the vertebrates. This result may reflect co-evolution between the ribosome and the region around the start ATG. The results reported in this subsection are in agreement with previous studies and motivated us to search for additional signals that are under selection near beginning of the ORF.

### Universal profile of decreased number of ATGs near the START ATG in all reading frames

In the previous section we demonstrated that there is a relatively weak signal corresponding to the START ATG in eukaryotes. This result raises the hypothesis that ATG codons near the beginning of the ORF contribute to alternative translation initiation events. If such alternative initiation events are deleterious we expect a selection for less ATGs near the beginning of the ORF.

In the rest of the paper, to analyze signals related to such a selection, we considered three reading frames: frame 0 is identical to the reading frame of the gene ORF; frames 1 and 2 represent a frame shift of 1 or 2 nucleotides relative to the main frame (Figure 2B).

**Figure 3:**
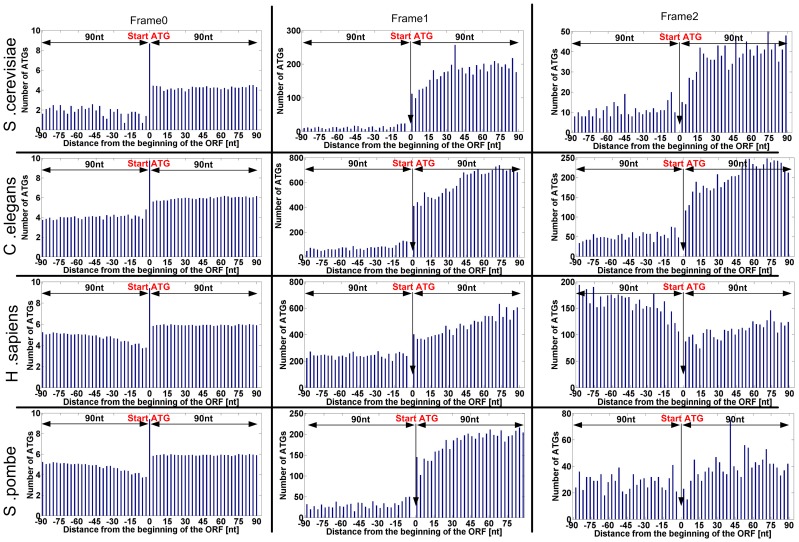
Genomic profiles of number of ATGs in the three frames for four model organisms (*S.* *cerevisiae*, *C. elegans*, *S. pombe*, *Human*). Each x-coordinate includes the total number of ATG codons (when considering all the analyzed genes) in this position; negative values of the x-axis correspond to the 5′UTR while positive values correspond to the ORF (the coordinate of the beginning of the ORF is 0). Each organism is described by three graphs, a graph for each frame; each such graph includes only the positions corresponding to the figure frame. In the case of frame 0, since there are many ATGs at the first position (main START ATG) of the ORFs, the Y-axis is log scaled (natural logarithm; specifically in position 0, the beginning of the ORF, the number is: log(#genes with 5′UTR).).

**Figure 4:**
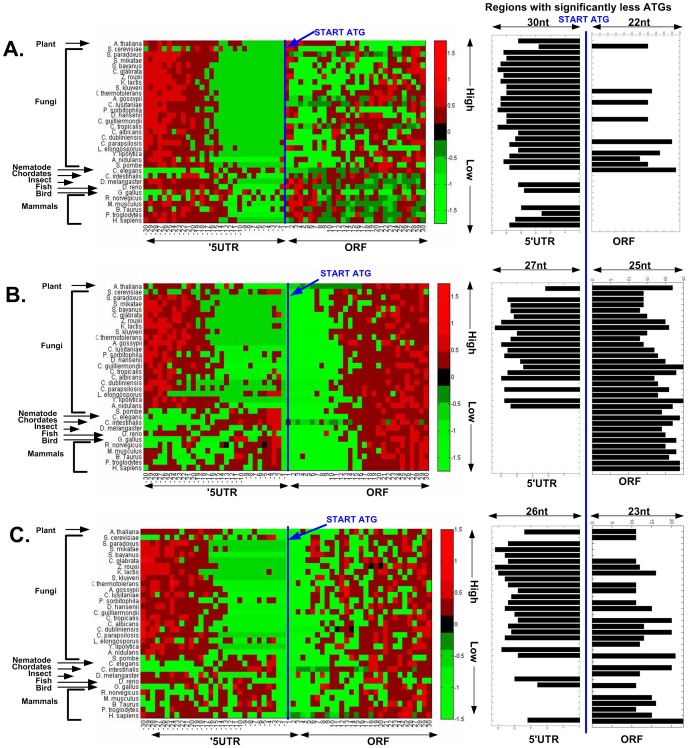
A–C. Genomic profiles of number of ATGs in the three frames of 33 eukaryotes' first thirty codons upstream, and downstream the START ATG (A. Frame 0, B. Frame 1, C. Frame 2). Left part: For each organism, in each nucleotide position, the number of ATGs was scaled according the distance (in terms of the number of STDs) from the mean number of ATGs across all the positions, for the 5′UTR and ORF separately. A position with relatively more ATG codons (positive number of STDs from the mean) was marked in red (more ATGs corresponds to a more reddish color), while positions with relatively less ATG codons (negative number of STDs from the mean) were marked in green (less ATGs corresponds to a more greenish color). Right part: the length of the region with significantly lower number of ATG codons near the START ATG in the 5′UTR and ORF for each organism (Methods). As can be seen, in all frames, there is a significant universal signal of fewer ATGs near the beginning of the ORF.

Indeed, as can be seen in Figure 3 for four model organisms (*S. cerevisiae*, *Caenorhabditis elegans*, *S. pombe*, and *Homo sapiens*), the number of alternative ATGs is lower near the beginning of the ORF. Similar results were obtained for the 33 eukaryotes that were analyzed (Figure 4). Specifically, in almost all the organisms and frame shifts there is a decrease in the number of ATGs near the end of the 5′UTR, but also the beginning of the ORF. For example, we found that the genomic region of significant decreased number of ATGs at the beginning of the ORF (see details in the Methods section regarding the estimation of this region) is 14/14/11 codons (*i.e.* triplets of nt) for 0, 1 and 2 nt frame shifts respectively in *S. cerevisiae* (Figure 3). Similar analysis was performed for the 5′UTR. The average region under selection for all the analysed organisms is 21/13/15 codons (16 across all frames) for the end of the 5′UTR, and 4/19/10 codons (11 across all frames) for the beginning of the ORF, for the three frames respectively (Figure 4).

### The profile of decreased number of ATGs near the START ATG in all reading frames is selected for

In the previous section we showed that there is a universal trend for a significantly lower number of ATGs near the beginning of the ORF. This result raises the question of whether these ATG profiles are selected for. For example, it is possible that the decrease in the number of ATGs near the beginning of the ORF is due to specific amino acid bias. Though the evolutionary selection for the ATG profile can be performed at the amino acid level and by non-synonymous mutations, we performed for the first time, an analysis to show that the signal remains, also when controlling for genomic features such as amino acid bias, codon bias and GC content.

**Figure 5:**
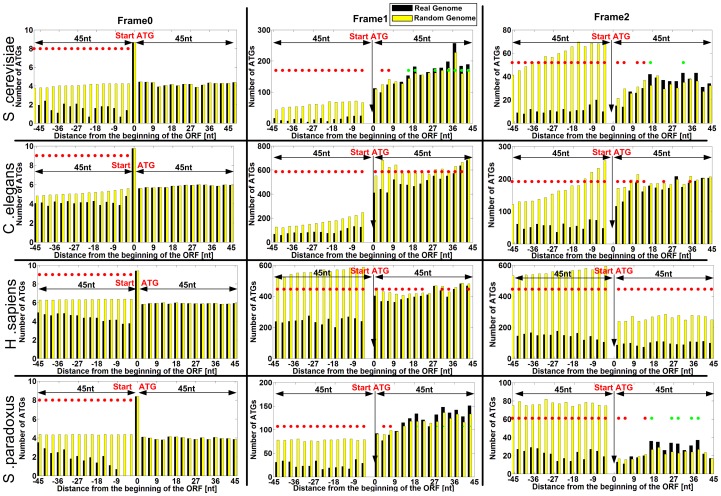
Comparison of the genomic profiles of number of ATGs in the three frames (in black), to the one obtained for randomized genomes (in yellow) with the same proteins, GC content, and codon bias, for four model organisms (*S.* *cerevisiae*, *C. elegans*, *H. sapiens* , *S. paradoxus*). A red/green dot corresponds to a position where the number of alternative ATG codons is significantly lower/higher than expected in the randomized genome model (details in the Methods section). The figure demonstrates that there is selection for less ATGs (red dots) in the 5′UTR and also at the beginning of the ORF. The positions with more ATGs than expected (green dots) after the beginning of the ORF may be related to yet unknown codon bias signals in that region. See supplementary Figures S1, S2, S3, S4, S5, S6, S7, S8, S9, S10, S11, S12, S13, S14, S15, S16, S17, S18, S19, S20, S21, S22, S23, S24, S25, S26, S27, S28, S29, S30, S31, S32, S33 for detailed figures of all 33 analyzed organisms, for two types of genome randomizations (Methods).

**Figure 6:**
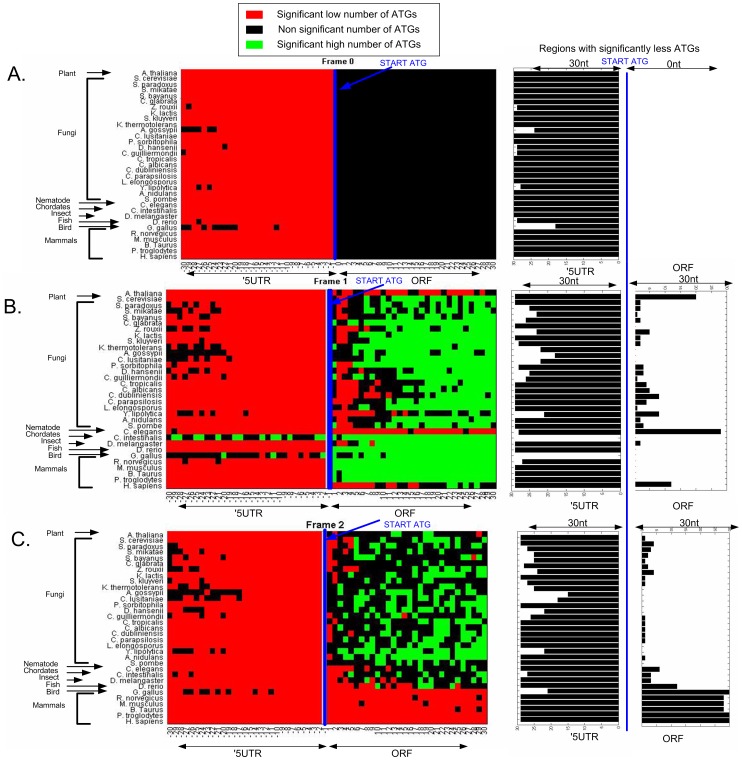
A–C. Genomic profiles of number of ATGs in the first thirty codons prior to, and following the START ATG, in comparison to 20 randomized genomes that maintain the amino acid order of each gene, the genomic GC content and codon usage bias, in the three frames (each sub-figure is related to one frame, A. Frame 0, B. Frame 1, C. Frame 2) for 33 eukaryotes (each row is one organism; each position within a row is related to a specific coordinate of the ATG codon before (5′UTR) or after the beginning of the ORF; Methods). Left part: There are three possible colors: Red – denotes cases where in that position the random genome includes significantly more ATGs than the real genome; green – denotes cases in which in that position the random genome includes significantly less ATGs than the real one; black – denotes that the random and real genomes are not significantly different in that position. Right part: the regions (5′UTR and ORF) of significantly lower number of ATG codons relatively to the randomized genomes for each organism and each frame (Methods). In all frames, the number of ATG codons in the 5′UTRs is significantly lower than expected. In frame 1 and 2 (according to our randomization frame 0 is identical in the random and real genome) the number of ATGs in most of the organisms is significantly lower in the first five codons. See Supplementary Figure S34 for permuted randomization (Methods).

Specifically, we compared the ATG profile obtained in each of the analyzed genomes to the one obtained in randomized genomes with identical proteins, total GC content and codon bias (see more details in the Methods section). As can be seen in Figure 5, the number of ATG codons near the beginning of the ORF indeed tends to be significantly lower than in the randomized genomes, supporting the conjecture that the observed ATG pattern is under selection. Expressly, in the analyzed organisms the 5′UTRs include a lower number of ATG codons than expected, but also fewer ATGs than expected can be found more than five codons downstream of the START codon. Interestingly, there are organisms with positions with more ATGs than expected (green dots in Figure 5) after the beginning of the ORF. This signal may be related (probably indirectly) to yet unknown codon bias signals after the beginning of the ORF. Analysis of the genomes of 33 eukaryotes by comparing them to randomized genomes (Methods) demonstrates that in most of them a region at the beginning of the ORF is under selection for less ATGs (Figure 6). Specifically the mean region under selection in the ORF for the analyzed genomes is 3.6 and 6.8 codons for the first and second frames respectively (frame 0 is the same for real and randomized genomes as we maintain the original proteins in the random genomes, see Methods and Figure 6), and 29.3, 25.3, and 26.8 in the 5′UTR for frame 0, 1 and 2 respectively.

**Figure 7:**
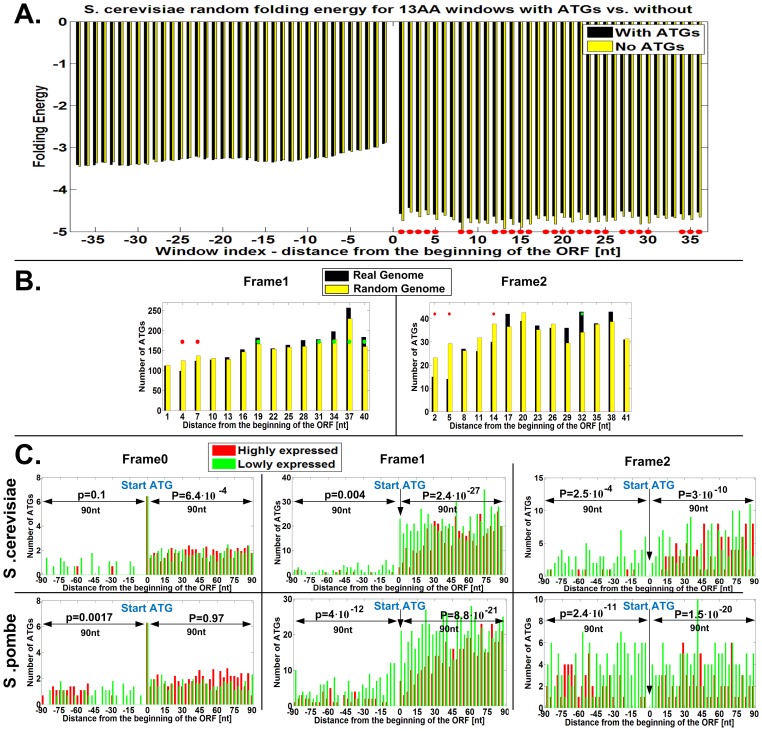
A. Comparison between the mRNA folding energy of randomized windows that maintain the window amino acid content (Methods) with ATGs and without ATGs. The folding energy between the two sets of windows is not significantly different at the 5′UTR; at the beginning of the ORF windows with more ATGs tend to have *weaker* mRNA folding. Positions with a significant p-value (paired t-test) are marked by a red dot. Thus, the fact that there is selection for weak mRNA folding at the beginning of genes cannot explain why there are less ATGs at the beginning of genes. B. Genomic profiles of number of ATGs in the first 14 codons following the START ATG, in comparison to 20 randomized genomes that maintain the *amino acid order of each gene*, *the genomic GC content and codon usage bias at the first 40 codons*, in frame 1 and 2 in *S. cerevisiae* (Methods). Red dots – denotes positions where the random genome includes significantly more ATGs than the real genome; green dots – denotes cases in which the random genome includes significantly less ATGs than the real one. C. The number of ATG profiles in the three frames in *S. cerevisiae* and *S. pombe* for highly and lowly expressed genes (top/bottom 15% ribosomal load). The fact that there are less ATGs at the *beginning of the ORF* is more striking in the case of the highly expressed genes. P-values for comparisons of the number of ATGs in the 5′UTR and ORF, and in each of the frames separately in the highly and lowly expressed genes, appear in the figure and are specifically significant for frame 1 and 2 (Methods; when considering all the frames together the 5′UTR/ORF p-values are 1.07·10^−12^ and 3.34·10^−27^ respectively in *S. cerevisiae*, and 8.75·10^−21^ and 8.6·10^−27^ respectively in *S. pombe*).

It was previously demonstrated that there is selection for lower folding strength at the beginning of the ORF, presumably to improve the efficiency of translation initiation [30], [31]. Thus, it is possible that the observed selection for a lower number of ATGs at the beginning of the ORF is a result of the selection for weak mRNA folding in these regions. To examine the relation between the number of ATGs in a short mRNA sequence and the folding energy, we randomized the sub-windows of mRNA sequences of the *S. cerevisiae* genome maintaining their amino acid content; for each sub-window across all genes we compared the folding energy of variants with at least one ATG codon to variants with no ATG codons (Methods). We found that decreasing the number of ATGs tends to *decrease* the folding energy (*i.e. increase* the folding strength); thus, the decrease in the number of ATG codons at the beginning of the ORF is clearly not a result of selection for weak mRNA folding at the beginning of the ORF (Figure 7A).

It was also demonstrated that there is a pattern of slower codons at the beginning (first 30–50 codons) of the ORF probably to improve the cost and fidelity of translation [38]; thus, it is possible that the observed signal of decreased number of ATGs at the beginning of the ORF is somehow related to this reported pattern. To show that this is not the case, we sampled randomized genomes that *maintain the distribution of codons at the beginning of the ORF* (Methods), and demonstrated that the signal of less ATGs at the beginning of the ORF in these randomized genomes is significantly weaker than in the case of the real genome (see Figure 7B).

In addition, if indeed translation tends to occur also from alternative ATG codons, and assuming that such an alternative translation initiation is usually deleterious, we expect stronger selection for less alternative ATG codons in highly translated genes (*e.g*. genes with higher ribosomal load), which potentially have higher effect on the organism fitness than genes with lower translation rates. Indeed, this is exactly the pattern that was observed when we compared the group of 15% top/bottom genes in terms of the ribosomal load; see p-values in figure 7C). This result is in agreement with the scanning model, according to which upstream ATGs should exert negative translational control (e.g. see [14], [18]); however, we found that such a signal appears also *downstream* of the start ATG.

Finally, our analysis shows that for each of the non-ATG codons there is no region with significantly lower number of codons before/after the beginning of the ORF. Thus, indeed the ATG codon behaves differently from non-ATG codons, supporting the hypothesis that the ATG depletion is related to translation initiation from the alternative ATG codons (see supplementary Figure S35).

### Profile of decreased ATG context scores

In this and the next subsections we report additional signals that are encoded near the beginning of the ORF to prevent alternative initiation of the ribosome, and thus to decrease the cost of translation.

At the first step, we considered the context of the alternative ATGs, which is related to the nucleotides in the vicinity of the main ATG codon. It has been demonstrated that the ribosome commences translation more efficiently from ATG codons with a specific context [10], [11]. Thus, we decided to verify if there is selection for ATG codons with less efficient contexts upstream and downstream the main START ATG. To this end, we developed a context score that is based on the distribution of nucleotides near the main START ATG in genes with a very high ribosomal-load. This score can be computed for every ATG, and higher scores reflect a context that is more similar to the main START ATG context of highly translated genes, and thus it is expected to contribute to a more efficient initiation (See details in the Methods section).

**Figure 8:**
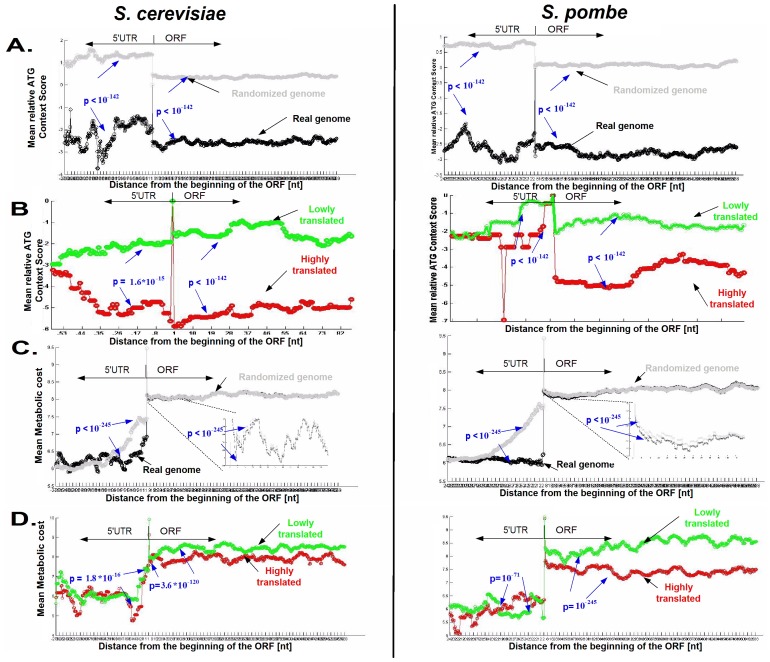
A. ATG context score profiles of real (black) *vs.* randomized (gray) genomes of *S. cerevisiae* and *S. pombe* near the beginning of the ORF. The real ATG context score profile is lower at all positions than the random profile. B. ATG context score profiles of genes with high (red) and low (green) ribosomal load in *S. cerevisiae* and *S. pombe* (top/bottom 10%). If non-main ATG codons appear in highly expressed genes they tend to have low context scores specifically near the beginning of the ORF. C. Metabolic cost of alternative initiation events for real (black) and randomized (gray) genomes of *S. cerevisiae* and *S. pombe*. D. Metabolic cost of translation commencing at alternative ATGs for highly (red) and lowly (green) expressed genes in terms of their ribosomal load for *S. cerevisiae* and *S. pombe* (top/bottom 10%). See supplementary Figure S36 for Distance to closest stop codon from alternative ATGs profiles.

We computed this score for each alternative ATG codon, and plotted the mean observed context score profile as a function of the distance from the beginning of the ORF over the entire genomes of *S. cerevisiae* and *S. pombe* (Figure 8). *S. cerevisiae* and *S. pombe* were selected as model organisms in this study due to the large evolutionary distance between them (they are estimated to have diverged approximately 350–1,000 million years ago [39]). Indeed, as expected, the context score surrounding the ATGs in the vicinity of the main START ATG tends to be lower in the real genomes relatively to the randomized genomes (Figure 8A; *S. cerevisiae*: 5′UTR p-value<10^−142^, ORF p-value<10^−142^; *S. pombe*: 5′UTR p-value<10^−142^, ORF p-value<10^−142^).

In addition, the context scores surrounding the ATGs s in the vicinity of the main START ATG in the case of genes that are highly translated, tend to be lower than in the case of genes that consume less ribosomes; specifically the difference between the two groups is larger around the beginning of genes (*S. cerevisiae*: 5′UTR p-value = 1.6·10^−15^, ORF p-value<10^−142^ ; *S. pombe*: 5′UTR p-value = 4.2·10^−142^, ORF p-value<10^−142^ ; Figure 8B).

### Selection for decreasing the metabolic cost of alternative translation initiation by a close STOP codon

An additional way to decrease the cost of alternative out-of frame undesired initiation events is to introduce a stop codon close to them. In such cases the translated peptide will be shorter and thus its translation and degradation will consume fewer cell resources. To check if indeed there is such a selection, we compared the distances of alternative ATG codons to the closest STOP codon in the same frame obtained for two groups of ATGs: 1) alternative ATG codons that are near the START ATG (less than 6 codons) and thus have higher probability of being involved in alternative translation initiation based on the scanning model; 2) alternative ATGs that are further from the START ATG. Indeed the distances were significantly shorter for the first group when looking across all frames, both when considering ATGs in the 5′UTR (mean 30.8 nt *vs.* 41.2 nt; p-value = 0.0008) and at the beginning of the ORF (mean 42 nt *vs.* 46.7 nt; p-value = 0.05). Similar results were obtained when we considered the metabolic biosynthesis cost (instead of peptide length, Methods) of the peptide potentially translated from alternative ATGs near the START ATG *vs.* further from the START ATG (5′UTR: mean 294.8 *vs.* 404 with p-value = 0.0001; ORF: mean 402.6 *vs.* 452.3 with p-value = 0.04); or if we also added the translation cost (Methods; in the case of the 5′UTR: 366.6 vs. 500.1 with p-value = 0.0002, in the case of the beginning of the ORF: 500.6 vs. 561.1 with p-value = 0.046). In addition, as can be seen in Figure 8C–D - the metabolic cost of translating alternative ATGs is lower than in randomized genomes (Figure 8C; *S. cerevisiae*: 5′UTR p-value<10^−245^, ORF p-value<10^−245^; *S. pombe*: 5′UTR p-value<10^−245^, ORF p-value<10^−245^; the results when considering the translation cost in addition to the metabolic costs were very similar – see the Methods section), and in highly expressed genes relatively to lowly expressed genes (Figure 8D; *S. cerevisiae*: 5′UTR p-value = 1.8·10^−16^, ORF p-value = 3.6·10^−120^; *S. pombe*: 5′UTR p-value = 10^−71^, ORF p-value = 10^−245^). Thus, these results support the conjecture that there is selection for close STOP codons near alternative ATG codons to decrease the metabolic cost of their translation.

### The predictive power of the new set of translation rules

The current study suggests and surveys a list of transcript features that are related to translation initiation; specifically these ‘rules’ include: 1) Fewer ATG codons at the end of the 5′UTR; 2) Fewer ATG codons at the beginning of the ORF ;3) Optimal Kozak/context sequence at the START ATG; 4) Anti-optimal Kozak/context sequence of ATG codons near the START ATG; 5) Close stop codon for alternative ATGs.

Do the new rules related to translation initiation described in the current paper also have predictive power? For instance, can they explain the variance in measured protein levels and ribosomal densities?

**Figure 9:**
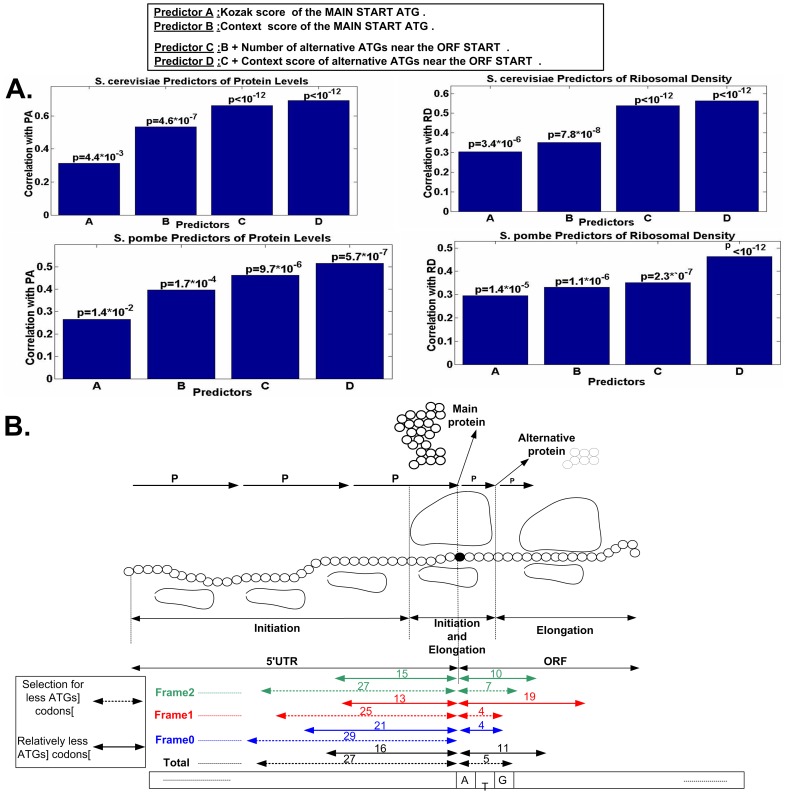
A. The predictive power of the new set of translation initiation rules. The correlation between four predictors based on increasing number of initiation rules described in this study with ribosomal density and protein levels in *S. pombe* and *S. cerevisiae*. B. A possible generalized model of translation initiation. *P* denotes the probability to continue the scanning stage of the pre-initiation complex; larger *P* denotes larger probability. The figure includes for each of the three frames, the mean (over all the analyzed organisms) genomic region at the end of the 5′UTR and the beginning of the ORF that is under selection for less ATGs, and the region that statistically includes less codons than the rest of the 5′UTR/ORF respectively (rounded to the closest integer).

To answer this question, we designed four computational predictors of protein levels and ribosomal density based on features and rules related to translation initiation described in this study; each of the following predictors was based on more features/rules in comparison to the previous ones (Methods). Four rules/features were considered: (1) The Kozak sequence in eukaryotes from [10]; (2) The main START ATG context score; (3) The number of alternative ATGs less than 30 codons downstream from the main START ATG; (4) The mean context scores of alternative ATGs less than 30 codons downstream from the main START ATG. We considered four predictors (*A*, *B*, *C*, and *D*); each predictor was based on an additional feature relatively to the previous one (*i.e. A* is based on feature (1); *B* is based on feature (2); *C* is based on features (2) and (3); *D* is based on features (2), (3), (4); see Figure 9B).

Indeed, as can be seen in Figure 9B, the correlation with binned protein levels and ribosomal density in *S. cerevisiae* (Methods) increases A) < B) <C) < D), *i.e.* this result supports the conjecture that we have designed a better model of initiation: the correlations with protein levels are 0.313/0.534/0.664/0.695 (p-values = 4.4·10^−3^/4.6·10^−7^/<10^−12^/<10^−12^) respectively, and the correlations with ribosomal density are 0.305/0.352/0.538/0.564 (p-values = 3.4·10^−6^/7.8·10^−8^/<10^−12^/<10^−12^) respectively. Similar results were obtained for *S. pombe* (see Figure 9B (, or when considering and controlling for the number of features in each predictor (Methods). Thus, the rules reported in this study can be utilized for designing highly expressed heterologous genes with efficient translation initiation.

## Discussion

In the current study we update and refine the set of rules related to the efficiency and the fidelity of translation initiation in eukaryotes. Specifically, we show that there are ancillary relevant signals upstream as required by the scanning model, but also *downstream* of the START ATG, in addition to a specific context surrounding the start ATG of the ORF reported in previous studies [10], [36]. While previous studies usually mentioned the possible contribution of the 5′UTR structure to translation initiation [4], [10], [14], [18], [37], [40], [41], we are the first to emphasize the selection against alternative ATG codons in the ORF. In addition, we are the first to study translation initiation signals in eukaryotes in a universal genome-wide manner, and the first to show that there is actually selection for the reported signals, while controlling for other possible explanations for these signals. Finally, we are the first to infer the region under selection for the reported signals; as discussed in the Methods section the inferred regions cannot be explained by the 5′UTR lengths.

The additional novel signals reported in this study include selection for less ATG codons before and after the main START ATG, but also more refined signals such as non-optimal nucleotide contexts near the alternative ATGs upstream and downstream of the main START ATG. We found that these signals are universal as they appear in many eukaryotes. In addition, these signals are selected for as they are stronger in highly expressed genes, and weaker in randomized genomes with the same proteins, GC-content, and codon bias as the original genome (even when considering the unusual distribution at the beginning of the ORF [38], see Methods). Thus, although there are cases where alternative ATG codons produce functional proteins [42]–[44], the results reported in this study demonstrate that specifically in the vicinity of the beginning of the ORF these ATG codons are usually deleterious and decrease the fitness of the organism. The mean estimated region under selection for less ATGs is the last 16.1--27.1 codons of the 5′UTR, and the first 5.2--11.2 codons of the beginning of the ORF; per-frame estimations appear in Figure 9B.

As we discuss in this section, the new initiation rules should improve the biophysical models of translation, the understanding of how translation efficiency is encoded in transcripts, and the constraints on the evolution of transcripts.

### An updated biophysical model of initiation

From a biophysical point of view, the results reported in this study can be used to develop more detailed models of translation initiation. For example, a possible initiation model that has been suggested [45] includes a diffusion/random-walk-type motion of the pre-initiation complex (note that other possible models may include energy-assisted directional scanning [45]). Under this model, the pre-initiation complex at each step has the probability to move forwards but also backwards along the transcript [45]. When the pre-initiation complex approaches an ATG codon it may start translating it with a probability that is related to the context of the ATG, but also to the *distance* of the ATG from the main START ATG [14]. Specifically, the probability of translating the main START ATG is lower than one, and the pre-initiation complex may continue scanning downstream of the START ATG. However, our analyses suggest that the probability to continue scanning after more than 5--11 codons downstream of the main START codon is negligible (illustration in Figure 9B) in terms of its effect on the organismal fitness – the selection signals after this region are not significant. The length of the region under selection is probably related to the biophysical properties of the pre-initiation complex such as its vibrations [46], its geometry, and the biophysical features of the mRNA molecules.

### Coupling between initiation and elongation

It was previously suggested that the translation elongation step is coupled in various ways with the initiation step via various signals encoded in the codons at the beginning of the coding sequences. For example, it was shown that the first codons of the coding sequences are under selection to induce weak local mRNA folding to improve the efficiency of translation initiation [30], [31], [47]. It was also shown that the codons at the beginning of the coding sequences have lower adaptation to the tRNA pool than the codons afterwards [38], and that the codons ∼10–25 downstream from the main ATG are under selection for strong mRNA folding [29], probably to improve ribosomal allocation by decreasing the initiation rate and increasing the distances between ribosomes.

The current study further demonstrates the coupling between the translation initiation and elongation steps. We suggest that additional signals for efficient and low-cost initiation appear not only at the 5′UTR but also at the beginning of the ORF (see also Figure 9B). These signals include amongst others lower numbers of ATGs and non-efficient ATG context scores in all reading frames. Thus, we suggest that the accepted paradigm that divides the translation process into three stages: initiation (occurs at the 5′UTR), elongation (at the coding sequence), and termination, is inaccurate. We propose an additional step that can be named late-initiation, which occurs at the region near the beginning of the ORF (Figure 9B). This step is part of the elongation stage and its efficiency is affected by signals encoded in the ORF, but is also related to the initiation step.

### The effect of alternative initiation events on organism fitness and evolution

The results reported in this study are also related to the ‘cost’ of translation. It is known that translation is the metabolic process that consumes the largest amount of cellular energy [2]. Here we suggest that to accurately estimate the translation cost we should also take into account the alternative initiation events near the beginning of the ORF. The various signals related to selection against such alternative initiation events suggest, that in the case of genes that do have ATG codons near the beginning of the ORF, there may be a non-negligible number of alternative initiation events. These events presumably consume significant amounts of energy both at the synthesis stage of these short peptides and at their degradation stage.

A related result in this context is the fact that the decrease in the in-frame codon frequency at the beginning of the ORF is weaker than in the case of the out-of-frame codons (see, for example, Figure 4; the effect of in-frame codons is significant in 9 organisms). However, *we actually do not expect that the signal in the in-frame codons will be as strong as in the case of out-of-frame or the 5′UTR signals* due to the following reasons:

1. The ATG codons that are in-frame after the beginning of the ORF constitute a special case in comparison to all the other regions/frames, as these codons are actually translated to the amino acid (Met) in the canonical condition; selection against such codons actually affects the amino-acid content, and thus the structure and function of the protein which is expected to be more ‘important’ and to have higher phenotypic effect on the organism fitness than preventing alternative initiation events.
2. When considering the region under strong selection at the beginning of the ORF (around the first 5 codons), initiation from in-frame alternative codons downstream and near the main START ATG should result in a protein with 1–5 less amino acids; on the other hand, initiation from alternative out-of-frame codons near the beginning of the ORF generate on average a 42 nt long new and probably undesired peptide. It is possible that the deleterious phenotypic effect of the first case is lower than the second case; thus, these ‘in-frame’ AUG codons near the beginning of the ORF are expected to be under lower purifying evolutionary selection.

### Functionality of the alternative proteins

The analyses described in this study suggest that evolution tends to eliminate events of alternative translation initiation. However, many alternative ATG codons still appear near the beginning of the ORFs in all the analyzed eukaryotes. It is possible that these ATGs still exist since the fitness advantage of eliminating these ATG codons is not high enough. An additional possibility is that at least some of the resultant alternative proteins from these alternative short peptides are functional. Indeed, recently it was demonstrated that many ATGs in the 5′UTR probably initiate translation in two model organisms, *Mus musculus* [48] and *S. cerevisiae* [49]. Cases where alternative translation initiation events affect the localization of the produced protein have also been reported in recent years (see, for example [50]–[56]). Our analyses suggest that this phenomenon may be much more widespread and appears in many other eukaryotes.

Finally, in agreement with the above paragraph, the observed selection for less ATG codons near the beginning of the ORF reported here does not contradict the fact that the alternative ATG codons that do appear near the beginning of the ORF are relatively conserved [57]. Actually, these two results support each other – the fact that alternative ATG codons may trigger alternative initiation events means that the alternative ATG codons that do appear near the beginning of the ORF probably tend to initiate translation; if they appear there (and haven't been selected for) it may mean that they have a (relatively important) functional role related to such alternative initiation, and thus should be more conserved relatively to other codons in this region.

### Co-evolution between the structure of the ribosome and the nucleotide region surrounding the START ATG

Analysis of the conservation of the nucleotides near the START ATG for 33 eukaryotes emphasized the importance and conservation of nucleotide position −3 [10], [36], [37] (Figure 2A). However, here we clearly show that the pattern of conservation of additional nucleotides varies along the evolutionary tree – different groups of eukaryotes have different conservation patterns. This result may be related to co-evolution between changes in the eukaryotic ribosomes along the evolutionary tree, and the nucleotide sequences that have the optimal interaction with them. Further future studies in this direction may teach us about the evolution of structural changes in the eukaryotic ribosome.

### Alternative transcription initiation vs. alternative translation initiation

We would like to conclude the discussion with a comparison between the transcription and the translation processes. Specifically, do we expect to see similar/analogous signals related to the transcription initiation fidelity as the ones we reported regarding translation initiation? We believe that the answer to this question is negative – these signals will be weaker in the case of transcription. There are several major dissimilarities making the two processes significantly different in this context. First, only in the case of translation are there three different frames that usually correspond to very different proteins; in the case of transcription there are no out-of frame initiation events, and a small shift should not affect the resultant protein. Second, alternative transcription initiation is also expected to affect the length of the 5′UTR and not the ORF, again not altering the produced proteins. Thus, we believe that the effect of alternative transcription on the organism fitness should be significantly lower than the effect of alternative translation.

## Methods

### Data sources

#### Ribosomal densities

For *S. cerevisiae* we consider two large scale Ribosomal Density (RD) measurements (the number of ribosomes occupying the transcript divided by its length); each generated by a different technology. The first dataset was generated more recently by Ingolia *et al*. [3], [58], and the second by [58]. We average across the two RD datasets (after normalizing each dataset by its mean), in order to minimize experimental noise. Similar results were obtained when we analyzed each dataset separately. *S. pombe* ribosomal densities were obtained from [59].

#### mRNA levels

For *S. cerevisiae* we considered two large scale measurements of mRNA levels from [3] and [60]. Similarly to RD, we averaged across the two datasets of mRNA levels. *S. pombe* mRNA levels were obtained from [59].

#### Protein Abundance

For *S. cerevisiae* we considered four quantitative large scale measurements of Protein Abundance (PA): [61], two large scale measurements in two conditions from [62], [63], and large scale protein abundance from [63]. Similarly to the RD, we averaged across the four datasets to reduce experimental noise. Similar results were obtained when we analyzed each dataset separately. *S. pombe*_protein abundance measurements were obtained from [64].

#### Metabolic costs of amino acids

Data for metabolic cost of amino acids for *S. cerevisiae* came from [65]. This was used as an approximation for *S. pombe* as no data was available.

#### Ribosomal-load, (mRNA levels)·(ribosomal density)

The number of ribosomes consumed by a gene is proportional to its mRNA levels and the ribosomal density on the mRNA molecules transcripted from the gene. Thus, we used ribosomal-load as a measure of the number of translation events of a gene.

#### UTRS and coding sequences

*S. cerevisiae* (EF4), *S. pombe* (EF1), *Arabidopsis thaliana* (TAIR10), *C. elegans* (WBcel215), *H. sapiens* (GRCh37.p2), *Bos taurus* (UMD3.1), *Ciona intestinalis* (KH), *Drosophila melangaster* (BDGP5), *Danio rerio* (Zv9), *Gallus gallus* (WASHUC2), *M. musculus* (NCBIM37), *Pan troglodytes* (CHIMP2.1.4) and *Rattus norvegicus* (RGSC3.4) genomes were downloaded from the Biomart Ensembl database; *Candida glabrata*, *Kluyveromyces lactis*, *Saccharomyces Kluyveri*, *Debaryomyces hansenii*, *Yarrowia lipolytica*, *Ashbya gossypii*, *Kluyveromyces thermotolerans*, *Pichia sorbitophila* and *Zygosaccharomyces rouxii* genomes were downloaded from [66]; *Saccharomyces paradoxus*, *Saccharomyces mikatae* and *Saccharomyces bayanus* genomes were downloaded from FGI (http://www.broadinstitute.org/scientific-community/science/projects/fungal-genome-initiative/fungal-genome-initiative); *Candida guilliermondii*, *Candida albicans*, *Candida lusitaniae*, *Candida parapsilosis*, *Lodderomyces elongisporus*, *Candida tropicalis* and *Candida dubliniensis* genomes were downloaded from *Candida* Genome Database (http://www.candidagenome.org/), *A. nidulans* genome was downloaded from [67].

#### Yeast 5′UTR reconstruction

*S. cerevisiae* 5′UTRs and 3′UTRs were obtained from [27]. The lengths of 5′UTRs and 3′UTRs are known for 4367 and 4972 genes respectively, for the 5861 *S. cerevisiae* genes we obtained. The mean length of known 5′UTRs is 82.33, while for 3′UTRs it is 133.62. The other 32 organisms mean UTR lengths can be found in supplementary Table S1.

### Computational Analysis

#### The ATG Context Score

Here we describe how this score is computed:

The PSSM Entropy was computed as follows: A position-specific scoring matrix (PSSM), is a commonly used representation of motifs in biological sequences. For each position (spanning 6 nts upstream and 3 nts downstream the main ATG) the entropy based on the empirical probabilities of the four possible nucleotides was computed based on the following formula:
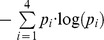
. Specifically, Figure 2 was generated in the following manner: For each of the 33 organisms we calculated a PSSM according to the nucleotide (C, A, T, G) appearance probability (frequency) in the entire genome, for 6 nts before and 3 nts after the first ATG (start) codon, based on the length of the potential optimal ATG context proposed by Hamilton *et al.* [68] . We then per organism PSSM calculated its entropy, such that for each nucleotide position the entropy is: -p(A)·log2(p(A)) -p(C)·log2(p(C)) -p(G)·log2(p(G)) -p(T)·log2(p(T)). Each organism's entropy values were then scaled according to their number of STDs distance from the mean.

ATG PSSM context score was computed as follows: We calculate the context score according to the following algorithm (scheme):

1. Select percentage of highly expressed/translated genes. Results are robust to versions of this procedure.
2. Calculate a position specific scoring matrix (PSSM) based on the nucleotide context around the start codon of the selected highly expressed genes. This PSSM represents the nucleotides necessary for highly efficient translation (Figure 1A).
3. Calculate the relative context score per ATG position according to the PSSM:
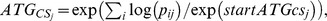
where j is the gene index, i the nucleotide position, 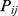 the probability that the i*th* nucleotide of the j*th* gene appears in the i*th* position, and 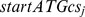 is the context score of the first ATG in the ORF (start codon).

We did not consider genes without reconstructed 5′UTRs or with 5′UTRs shorter than 6 nt.

The following include more details about stages 1–2.

The training set was selected as follows: The training set for the PSSM calculation is based on the selection of the top 2% of highly expressed genes, according to the ribosomal load [3], [58], which represents the total flux of ribosomes over a gene. However, similar results were achieved when selecting the highly expressed group of genes based solely on ribosomal density or mRNA levels, and also when based on other measures of expression, such as protein abundance etc. In order to avoid over fitting, we select the top 4% of the highly expressed genes and then randomly select half of that group as the training set. Different percentages of training set sizes (up to 50%) were also tested in the same manner, and the results are robust to training set size variance.

The PSSM was calculated as followed: The PSSM was calculated according to the nucleotide (C, A, T, G) appearance probability (frequency), of the training set (2% selected at (1)), for 6 nts before and 3 nts after the first ATG (start) codon, based on the length of the potential optimal ATG context proposed by Hamilton *et al.* [68]. We achieved similar results when considering various context lengths. Interval values between 3 to 48 nts around the start codon were examined, for symmetric and asymmetric combinations, in jumps of 3 nts, and additionally in jumps of 1 nt from 1 nt up to 10 nts, and the results are robust to interval variance. These intervals were tested for all the aforementioned training set sizes in (1), and the results remain robust. Indeed, as can be seen in Figure 2, the nts further from the START ATG are less relevant.

The ATG context score profile was calculated as follows: Utilizing the context score measure, we calculated a position specific ATG context score profile spanning the position specific ATG appearance in the 5′UTR, the start codon, and the ATGs up to 200 codons downstream from the start codon, where for each gene, for each in-frame/out-of-frame ATG with the start codon, a context score was calculated. A profile was then generated, where the genes' ATG context scores were averaged across sliding windows of 30 nts (similarly to the ribosomal footprint [3]), with a slide of 1 nt). A context score profile of a subset of genes was computed in a similar way while considering only the genes in the subset. For example, Figure 8B depicts the ATG context score profile as described above for the top 10% highly expressed genes, and bottom 10% lowly expressed genes, for *S. cerevisiae* and *S. pombe*. Training set genes were excluded in this stage to avoid over-fitting.

#### ATG analysis of the different frames

There are three possible frames relative to the main ATG start codon. Specifically, an alternative codon can be in: frame 0, the same frame as the main ATG, frame 1, a 1 nt shift from the main ATG's frame, frame 2, a 2 nt shift from the main ATG's frame.

#### Comparison to previous correlations

The correlations between measures of ATG contexts and expression levels reported in previous papers were based on much fewer numbers of genes and thus slightly differ from the ones reported here. The context score used in this study yielded similar but higher correlations with protein levels in *S. cerevisiae*. Thus, similar results could be obtained with alternative context scores (*e.g.* when considering the proteome of *S. cerevisiae* and *S. pombe*, the correlations between the AugCAI [69] and protein levels is 0.186 [p-value = 1.7·10^−11^] and 0.082 [p-value = 2.56·10^−3^], respectively; while the correlation between our context score and protein levels is 0.231 [p-value = 3.6·10^−17^] and 0.173 [p-value = 1.2·10^−10^], respectively). Here, we briefly describe the alternative context scores suggested in previous papers.

We start with the Kozak score: Around 30 years ago Kozak [10] suggested that there is a specific optimal context surrounding the ATG start codon. Specifically, she identified ACCATGG as the optimal sequence for initiation by eukaryotic ribosomes. In this case the context score is the hamming distance (or the number of mutations) from this optimal context. In vivo, this site is often not matched exactly on different mRNAs and the amount of protein synthesized from a given mRNA is dependent on the strength of the Kozak sequence. We found that few (0.0048% in *S. cerevisiae* and 0.0068% in *S. pombe*) of the sequences satisfy the Kozak rule precisely (with an average hamming distance of 0.67 in *S. cerevisiae* and *S. pombe*). Similar conclusions have been reported in previous studies [19], [21].

We continue with the index of Miyasaka: Miyasaka suggested an index for the measure of optimal ATG context called the AugCAI, which is also based on the nt distribution near the start ATG of highly expressed genes in *S. cerevisiae* [69].

#### The predictors of protein levels and ribosomal density

We designed four computational predictors of protein levels (PA) and ribosomal density (RD) based on features and rules related to translation initiation described in this study. Four rules/features were considered: 1) The Kozak sequence in eukaryotes from [10]; 2) The START ATG context score; 3) The number of alternative ATG codons less than 30 codons downstream from the START ATG; 4) The mean context scores of alternative ATGs less than 30 codons downstream from the START ATG. From these features we built predictors *A)*, *B)*, *C)*, *D)*, such that they were based on features/rules, 1), 2), 2) and 3), 2) and 3) and 4) respectively, as follows. First, in order to filter noise we binned the data according to descending order of PA and RD respectively, with each bin containing 15 elements. Similar results were achieved for various bin sizes ranging from 1–50. Then each of the 8 predictors, 4 for PA, and 4 for RD, were constructed for *S. cerevisiae* and *S. pombe* respectively as described above. We performed 100 2-fold cross validations, showing the predictors results are robust.

When calculating the adjusted correlations that also take into account the number of features (see, for example, [70]), we achieved similar results to the ones obtained for regular correlation (main text) for predictors A/B/C/D respectively: in *S. cerevisiae*, the adjusted correlations with protein levels are 0.287/0.525/0.661/0.687, and with ribosomal density are 0.268/0.346/0.532/0.559. Similar results were obtained for *S. pombe*, with adjusted correlations with protein levels of 0.264/0.385/0.446/0.474, and with ribosomal density of 0.31/0.325/0.352/0.458.

We would like to emphasize that we removed the train test used to infer the ATG PSSM/‘ATG context score’ from this analysis. Thus, the reported results are not due to over-fitting.

#### Generating random genomes

We performed two kinds of randomizations of the genomes of 33 eukaryotes. In the first randomization, each random version of the genome (we generated a total of 20 random genomes) was generated in the following manner: for each amino acid in each gene, we sampled a codon while considering the genomic codon frequency/codon-bias in that genome (that is, more frequent codons in the genome have a higher probability of being sampled). Thus, the randomized genomes maintained both the amino acid content of each coding sequence, and the codon frequencies of the original genome. Randomization of the UTRs was achieved by permuting their nucleotides.

In the second randomization (we generated a total of 20 random genomes) the codons of the coding sequence were permuted, with the UTRs randomized similarly to the first randomization.

See supplementary Figure S34 for Genomic profiles of number of ATGs in the three frames of 33 eukaryotes for the second randomization, and Figure 6 for the first randomization.

Finally, Tuller *et al.* showed that there is a ramp of slower codons at the beginning of the ORF, spanning a region of around 40 codons [38]. We show that even if we sample randomized genomes that maintain the distribution of codons at the beginning of the ORF (similarly to the methodology described above, only now we sample codons for the first 40 codons of the ORF according to the codon distribution only in this region), the signal of less ATGs at the beginning of the ORF is weaker than in the case of the real genome (Figure 7B).

#### Randomized folding energy analysis

We employed the Matlab rnafold function (Matlab Bioinformatics toolbox), which predicts the folding energy of the secondary structure associated with the minimum free energy for an RNA sequence (or subsequence).

The *S. cerevisiae* genome we obtained includes 5861 genes. For each gene, we considered sliding windows of length 13 codons (slide of 1 nt; 13 AA is the approximated length of the ribosomes' footprint [3]; similar results were obtained with small changes to this parameter), that are up to 37 nt upstream and downstream of the main start ATG.

For each window, in each gene, we performed 20 randomizations – in the case of the ORF we performed a randomization that maintains the amino acid content, in the case of the UTR we maintained the GC content, as described above. Thus for each of the 74·5861 windows we created 20 versions that induce 20 predictions of folding energy, and 20 counts of ATGs (we count the number of ATGs in all the reading frames).

For each of the 74·5861 windows we computed 2 values:

1. The mean folding energy related to all the versions that do not include any ATGs (in all the frames).
2. The mean folding energy related to all the versions that include at least one ATG (in any of the reading frames).

We ignore cases where one of the groups above is empty.

Considering each of the 74 positions separately we devised two vectors (of length 5861) representing the aforementioned groups, on which we performed a paired t-test. The results are depicted in Figure 7A. This statistic test checks if randomized windows that include ATGs tend to have lower/higher folding energy than windows that do not include ATGs, when controlling for amino acid content and the position along the transcript.

#### Metabolic cost and peptide length

The total energy cost of amino acids in *S. cerevisiae* under respiratory conditions were taken from [65] , and the metabolic cost of a peptide was calculated as the sum of the energy cost of the amino acids composing it. Peptide length was measured as the number of nucleotides/codons (depending on the application) composing it.

#### The metabolic cost of translating alternative ATGs is lower than in randomized genomes

In order to ascertain that there is selection for a lower metabolic cost of alternative initiation events, we compared the genomes of *S. cerevisiae* and *S. pombe* to randomized genomes which were generated similarly to the first randomization in section ‘Generating random genomes’, generating 20 randomized genomes. The difference being that here we wanted to estimate the distance to the closest stop codon while controlling for the number of ATGs (that may be different in the randomized and real genomes). Therefore, in each randomized gene, we planted the ATGs of the original genome, and measured the metabolic cost of the peptide defined by the closest stop codon to each alternative ATG in the randomized version (see Figure 8C).

#### There are less ATGs in highly expressed genes near the START ATG

We compared the group of 15% top/bottom genes with respect to the ribosomal-load, in terms of number of ATGs in the first 30 codons upstream and downstream of the main ATG per frame. We performed a ks-test in the following manner. For each gene per frame, and for all frames together, we counted the number of ATGs in the 30 codons upstream and 30 codons downstream of the main ATG separately, for the group of highly and lowly expressed genes respectively. This resulted in 8 ‘number of ATGs’ vectors, where for each region, for each frame and for all frames together, we performed the ks-test to determine the significance of the difference between the highly and lowly expressed genes (see Figure 7C).

#### Calculating region of decreased number of ATGs near the START ATG in all reading frames

In order to test the significance of the lower number of ATGs in the beginning of the ORF we performed the following statistic test. Looking at sliding windows of length 11 codons (the approximate size of the ribosome, with a slide of 1 codon), for each of the frames separately in the first 90 nts of the ORF, we perform a ks-test between the mean number of ATGs per nucleotide in each such window and the remainder of the region. If the first window of the ORF was not significant we determined that there is no region of lower number of codons at the beginning of the ORF. Otherwise, the region corresponding to lower number of ATGs at the beginning of the ORF was defined as: (the size of the window) −1 + (number of consecutive windows in the beginning of the ORF which achieved a significant p-value [<0.05]). To calculate the mean significant region across the 33 eukaryotes analyzed in this study, we averaged the length of the significant region across all frames per organism, and then averaged the resultant values across all the organisms. The mean region found to be significant in the ORF is 11.2. We performed the same analysis for the 5′UTR and the mean region found to be significant is 16.1.

#### Calculating significant mean number of codons in the vicinity of the START ATG which exhibit universal trend of selection for low numbers of ATG codons

Utilizing the 20 randomized genomes generated as explained in the first part of the ‘Generating random genomes’ section, we calculated an empirical p-value showing that there is selection for a lower number of ATG codons in the beginning of the ORF, and end of the 5′UTR, as follows. Looking at every nucleotide position in the 90 nt prior to and following the START ATG, for frames 1 and 2 (as frame 0 for the ORF is identical in the real and random genomes in regard to ATGs), a nucleotide position was found to have significantly less ATGs than the random, if 95% or more of the random genomes had more ATGs than the real genome for that position. A nucleotide position was found to have significantly more ATGs than the random, if 95% or more of the random genomes had less ATGs than the real genome for that position. Similarly to the analysis above, to calculate the mean significant region across the 33 eukaryotes analyzed in this study we averaged the length of the significant region across frames 1 and 2 per organism, and then averaged the resultant values across all the organisms. Indeed we found that both at the end of the 5′UTR, region of length 27.1, and the beginning of the ORF, region of length 5.2, there is selection for fewer ATGs.

#### The effect of the 5′UTR lengths on the reported results

The reported results are robust to the length of the 5′UTR. Specifically, the fact that there are less ATGs before and after the beginning of the ORF (Figure 4) can't be explained by the fact that the 5′UTR lengths vary among the different genes: considering the length of the 5′UTR should actually increase (or not change) the signal as we expect relatively *more* ATGs at the 5′UTR regions that are closer to the beginning of the ORF (if *i > j* there are more 5′UTRs longer than *j* than number of 5′UTRs longer than *i*; thus under uniform distribution of ATGs we expect to see more ATGs at distance *j* from the beginning of the ORF than at distance *i* from the beginning of the ORF). In addition, the comparison to randomized genomes (Figure 6) should not be affected by the different lengths of the 5′UTRs as we maintained the same 5′UTR lengths in the randomized genomes. Finally, the upper and lower bounds on the region ‘under selection’ that we report in the study (*e.g.* Figure 9B) are also tight and conservative: in the case of the 5′UTR, the upper bound is related to comparison to randomized genomes (that should not be affected by genome size), while the lower bound is based on the relative number of codons which can only be larger if we consider the length of the 5′UTR.

#### Alternative ATGs distance to a stop codon and the induced metabolic cost of alternative peptides

Looking at out of frame ATG codons 300 nt prior to and following the main Start ATG, we calculated the average distance to the closest stop codon for two groups of ATGs, in the 5′UTR and ORF respectively, those under 6 codons upstream (in the 5′UTR), and under 6 codons downstream (in the ORF), from the main start ATG, and the remainder of the respective 300 nt region (the first 6 codons is the region found to be under selection for less ATGs in *S. cerevisiae*). We then calculated the metabolic cost (based on [65]) of synthesizing the peptide induced by the closest stop codon. We checked the significance of the shorter (and metabolically cheaper) potential peptides of the first group (≤6 codons), as compared to the second (remaining 300 nt region), in the 5′UTR and ORF respectively, by performing a Kolmogorov–Smirnov test. The results remained similar when adding the translation cost of 7ATPs to the metabolic synthesis cost of each AA (according to the canonical model the translation cost of an amino acid includes: 2ATP for tRNA charging, 1 for codon decoding, 1 for translocation, 1 for E-site tRNA release; but due to futile cycles is more like 7 ATP per incorporated amino acid in vivo).

#### Organization of the analyzed organisms based on their evolutionary distance and taxonomy

The organisms in Figures 2A, 4, 6 were organized based on phylogenetic distance and taxonomic information. To this end, we employed phylogenetic information from [71]–[76] and taxonomic information from Pubmed.

## Supporting Information

Figure S1 A–C. *S. cerevisiae* comparison of the genomic profiles of number of ATGs in the three frames to the ones obtained for randomized genomes with the same proteins, GC content, and codon bias (Methods). D–F. *S. cerevisiae* comparison of the genomic profiles of number of ATGs in the three frames to the ones obtained for randomized genomes that were generated by permuting the codons of each gene (Methods).
(TIF)

Figure S2 A–C. *S. pombe* comparison of the genomic profiles of number of ATGs in the three frames to the ones obtained for randomized genomes with the same proteins, GC content, and codon bias (Methods). D–F. *S. pombe* comparison of the genomic profiles of number of ATGs in the three frames to the ones obtained for randomized genomes that were generated by permuting the codons of each gene (Methods).
(TIF)

Figure S3 A–C. *A. thaliana* comparison of the genomic profiles of number of ATGs in the three frames to the ones obtained for randomized genomes with the same proteins, GC content, and codon bias (Methods). D–F. *A. thaliana* comparison of the genomic profiles of number of ATGs in the three frames to the ones obtained for randomized genomes that were generated by permuting the codons of each gene (Methods).
(TIF)

Figure S4 A–C. *C. elegans* comparison of the genomic profiles of number of ATGs in the three frames to the ones obtained for randomized genomes with the same proteins, GC content, and codon bias (Methods). D–F. *C. elegans* comparison of the genomic profiles of number of ATGs in the three frames to the ones obtained for randomized genomes that were generated by permuting the codons of each gene (Methods).
(TIF)

Figure S5 A–C. *H. sapiens* comparison of the genomic profiles of number of ATGs in the three frames to the ones obtained for randomized genomes with the same proteins, GC content, and codon bias (Methods). D–F. *H. sapiens* comparison of the genomic profiles of number of ATGs in the three frames to the ones obtained for randomized genomes that were generated by permuting the codons of each gene (Methods).
(TIF)

Figure S6 A–C. *A. gossypii* comparison of the genomic profiles of number of ATGs in the three frames to the ones obtained for randomized genomes with the same proteins, GC content, and codon bias (Methods). D–F. *A. gossypii* comparison of the genomic profiles of number of ATGs in the three frames to the ones obtained for randomized genomes that were generated by permuting the codons of each gene (Methods).
(TIF)

Figure S7 A–C. *A. nidulans* comparison of the genomic profiles of number of ATGs in the three frames to the ones obtained for randomized genomes with the same proteins, GC content, and codon bias (Methods). D–F. *A. nidulans* comparison of the genomic profiles of number of ATGs in the three frames to the ones obtained for randomized genomes that were generated by permuting the codons of each gene (Methods).
(TIF)

Figure S8 A–C. *C. albicans* comparison of the genomic profiles of number of ATGs in the three frames to the ones obtained for randomized genomes with the same proteins, GC content, and codon bias (Methods). D–F. *C. albicans* comparison of the genomic profiles of number of ATGs in the three frames to the ones obtained for randomized genomes that were generated by permuting the codons of each gene (Methods).
(TIF)

Figure S9 A–C. *C. dubliniensis* comparison of the genomic profiles of number of ATGs in the three frames to the ones obtained for randomized genomes with the same proteins, GC content, and codon bias (Methods). D–F. *C. dubliniensis* comparison of the genomic profiles of number of ATGs in the three frames to the ones obtained for randomized genomes that were generated by permuting the codons of each gene (Methods).
(TIF)

Figure S10 A–C. *C. glabrata* comparison of the genomic profiles of number of ATGs in the three frames to the ones obtained for randomized genomes with the same proteins, GC content, and codon bias (Methods). D–F. *C. glabrata* comparison of the genomic profiles of number of ATGs in the three frames to the ones obtained for randomized genomes that were generated by permuting the codons of each gene (Methods).
(TIF)

Figure S11 A–C. *C. guilliermondii* comparison of the genomic profiles of number of ATGs in the three frames to the ones obtained for randomized genomes with the same proteins, GC content, and codon bias (Methods). D–F. *C. guilliermondii* comparison of the genomic profiles of number of ATGs in the three frames to the ones obtained for randomized genomes that were generated by permuting the codons of each gene (Methods).
(TIF)

Figure S12 A–C. *C. lusitaniae* comparison of the genomic profiles of number of ATGs in the three frames to the ones obtained for randomized genomes with the same proteins, GC content, and codon bias (Methods). D–F. *C. lusitaniae* comparison of the genomic profiles of number of ATGs in the three frames to the ones obtained for randomized genomes that were generated by permuting the codons of each gene (Methods).
(TIF)

Figure S13 A–C. *C. parapsilosis* comparison of the genomic profiles of number of ATGs in the three frames to the ones obtained for randomized genomes with the same proteins, GC content, and codon bias (Methods). D–F. *C. parapsilosis* comparison of the genomic profiles of number of ATGs in the three frames to the ones obtained for randomized genomes that were generated by permuting the codons of each gene (Methods).
(TIF)

Figure S14 A–C. *C. tropicalis* comparison of the genomic profiles of number of ATGs in the three frames to the ones obtained for randomized genomes with the same proteins, GC content, and codon bias (Methods). D–F. *C. tropicalis* comparison of the genomic profiles of number of ATGs in the three frames to the ones obtained for randomized genomes that were generated by permuting the codons of each gene (Methods).
(TIF)

Figure S15 A–C. *D. hansenii* comparison of the genomic profiles of number of ATGs in the three frames to the ones obtained for randomized genomes with the same proteins, GC content, and codon bias (Methods). D–F. *D. hansenii* comparison of the genomic profiles of number of ATGs in the three frames to the ones obtained for randomized genomes that were generated by permuting the codons of each gene (Methods).
(TIF)

Figure S16 A–C. *K. lactis* comparison of the genomic profiles of number of ATGs in the three frames to the ones obtained for randomized genomes with the same proteins, GC content, and codon bias (Methods). D–F. *K. lactis* comparison of the genomic profiles of number of ATGs in the three frames to the ones obtained for randomized genomes that were generated by permuting the codons of each gene (Methods).
(TIF)

Figure S17 A–C. *K. thermotolerans* comparison of the genomic profiles of number of ATGs in the three frames to the ones obtained for randomized genomes with the same proteins, GC content, and codon bias (Methods). D–F. *K. thermotolerans* comparison of the genomic profiles of number of ATGs in the three frames to the ones obtained for randomized genomes that were generated by permuting the codons of each gene (Methods).
(TIF)

Figure S18 A–C. *L. elongosporus* comparison of the genomic profiles of number of ATGs in the three frames to the ones obtained for randomized genomes with the same proteins, GC content, and codon bias (Methods). D–F. *L. elongosporus* comparison of the genomic profiles of number of ATGs in the three frames to the ones obtained for randomized genomes that were generated by permuting the codons of each gene (Methods).
(TIF)

Figure S19 A–C. *P. sorbitophila* comparison of the genomic profiles of number of ATGs in the three frames to the ones obtained for randomized genomes with the same proteins, GC content, and codon bias (Methods). D–F. *P. sorbitophila* comparison of the genomic profiles of number of ATGs in the three frames to the ones obtained for randomized genomes that were generated by permuting the codons of each gene (Methods).
(TIF)

Figure S20 A–C. *S. bayanus* comparison of the genomic profiles of number of ATGs in the three frames to the ones obtained for randomized genomes with the same proteins, GC content, and codon bias (Methods). D–F. *S. bayanus* comparison of the genomic profiles of number of ATGs in the three frames to the ones obtained for randomized genomes that were generated by permuting the codons of each gene (Methods).
(TIF)

Figure S21 A–C. *S. Kluyveri* comparison of the genomic profiles of number of ATGs in the three frames to the ones obtained for randomized genomes with the same proteins, GC content, and codon bias (Methods). D–F. *S. Kluyveri* comparison of the genomic profiles of number of ATGs in the three frames to the ones obtained for randomized genomes that were generated by permuting the codons of each gene (Methods).
(TIF)

Figure S22 A–C. *S. mikatae* comparison of the genomic profiles of number of ATGs in the three frames to the ones obtained for randomized genomes with the same proteins, GC content, and codon bias (Methods). D–F. *S. mikatae* comparison of the genomic profiles of number of ATGs in the three frames to the ones obtained for randomized genomes that were generated by permuting the codons of each gene (Methods).
(TIF)

Figure S23 A–C. *S. paradoxus* comparison of the genomic profiles of number of ATGs in the three frames to the ones obtained for randomized genomes with the same proteins, GC content, and codon bias (Methods). D–F. *S. paradoxus* comparison of the genomic profiles of number of ATGs in the three frames to the ones obtained for randomized genomes that were generated by permuting the codons of each gene (Methods).
(TIF)

Figure S24 A–C. *Y. lipolytica* comparison of the genomic profiles of number of ATGs in the three frames to the ones obtained for randomized genomes with the same proteins, GC content, and codon bias (Methods). D–F. *Y. lipolytica* comparison of the genomic profiles of number of ATGs in the three frames to the ones obtained for randomized genomes that were generated by permuting the codons of each gene (Methods).
(TIF)

Figure S25 A–C. *Z. rouxii* comparison of the genomic profiles of number of ATGs in the three frames to the ones obtained for randomized genomes with the same proteins, GC content, and codon bias (Methods). D–F. *Z. rouxii* comparison of the genomic profiles of number of ATGs in the three frames to the ones obtained for randomized genomes that were generated by permuting the codons of each gene (Methods).
(TIF)

Figure S26 A–C. *B. Taurus* comparison of the genomic profiles of number of ATGs in the three frames to the ones obtained for randomized genomes with the same proteins, GC content, and codon bias (Methods). D–F. *B. Taurus* comparison of the genomic profiles of number of ATGs in the three frames to the ones obtained for randomized genomes that were generated by permuting the codons of each gene (Methods).
(TIF)

Figure S27 A–C. *C. intestinalis* comparison of the genomic profiles of number of ATGs in the three frames to the ones obtained for randomized genomes with the same proteins, GC content, and codon bias (Methods). D–F. *C. intestinalis* comparison of the genomic profiles of number of ATGs in the three frames to the ones obtained for randomized genomes that were generated by permuting the codons of each gene (Methods).
(TIF)

Figure S28 A–C. *D. melangaster* comparison of the genomic profiles of number of ATGs in the three frames to the ones obtained for randomized genomes with the same proteins, GC content, and codon bias (Methods). D–F. *D. melangaster* comparison of the genomic profiles of number of ATGs in the three frames to the ones obtained for randomized genomes that were generated by permuting the codons of each gene (Methods).
(TIF)

Figure S29 A–C. *D. rerio* comparison of the genomic profiles of number of ATGs in the three frames to the ones obtained for randomized genomes with the same proteins, GC content, and codon bias (Methods). D–F. *D. rerio* comparison of the genomic profiles of number of ATGs in the three frames to the ones obtained for randomized genomes that were generated by permuting the codons of each gene (Methods).
(TIF)

Figure S30 A–C. *G. gallus* comparison of the genomic profiles of number of ATGs in the three frames to the ones obtained for randomized genomes with the same proteins, GC content, and codon bias (Methods). D–F. *G. gallus* comparison of the genomic profiles of number of ATGs in the three frames to the ones obtained for randomized genomes that were generated by permuting the codons of each gene (Methods).
(TIF)

Figure S31 A–C. *M. musculus* comparison of the genomic profiles of number of ATGs in the three frames to the ones obtained for randomized genomes with the same proteins, GC content, and codon bias (Methods). D–F. *M. musculus* comparison of the genomic profiles of number of ATGs in the three frames to the ones obtained for randomized genomes that were generated by permuting the codons of each gene (Methods).
(TIF)

Figure S32 A–C. *P. troglodytes* comparison of the genomic profiles of number of ATGs in the three frames to the ones obtained for randomized genomes with the same proteins, GC content, and codon bias (Methods). D–F. *P. troglodytes* comparison of the genomic profiles of number of ATGs in the three frames to the ones obtained for randomized genomes that were generated by permuting the codons of each gene (Methods).
(TIF)

Figure S33 A–C. *R. norvegicus* comparison of the genomic profiles of number of ATGs in the three frames to the ones obtained for randomized genomes with the same proteins, GC content, and codon bias (Methods). D–F. *R. norvegicus* comparison of the genomic profiles of number of ATGs in the three frames to the ones obtained for randomized genomes that were generated by permuting the codons of each gene (Methods).
(TIF)

Figure S34 A–C. Genomic profiles of number of ATGs in the three frames of 33 eukaryotes' permuted randomized genomes' (Methods) first thirty codons upstream, and downstream the START ATG (A.- Frame 0 which is the reading frame of the protein, B. – Frame 1: 1 nt frame shift from the reading frame, C. – Frame 2: 2 nt frame shift from the reading frame). Each organism's number of ATGs were scaled according to the number of STDs distance from the mean, for the 5′UTR and ORF separately. As can be seen, in all frames, there is a universal signal of fewer ATGs near the beginning of the ORF.
(TIF)

Figure S35 Genomic profiles of number of *non-ATG* codons in the three frames in *S. cerevisiae'*s first forty codons upstream, and downstream the START ATG (A. Frame 0, B. Frame 1, C. Frame 2). For each organism, in each nucleotide position, the number of appearances of a codon was scaled according to the distance (in terms of the number of STDs, after normalizing by the maximal possible number of appearances of the codon in the position) from the mean number of appearances of the codon across all the positions, for the 5′UTR and ORF separately. A position with relatively more appearances of a codon (positive number of STDs from the mean) was marked in red (more appearances corresponds to a more reddish color), while positions with relatively less appearances of a codon (negative number of STDs from the mean) were marked in green (less appearances corresponds to a more greenish color). For each codon, for both the 5′UTR and the non-main frame shifts of the ORF, the length of the region with significantly lower number of appearances of the codon (Figure 4) was zero (*i.e.* there is no such region); this result demonstrates that the ATG codon behaves differently from non-ATG codons, supporting the hypothesis that the ATG depletion is related to translation initiation from the alternative ATG codons.
(TIF)

Figure S36 A. Distance to closest stop codon from alternative ATGs for real (black) and randomized (gray) genomes of *S. cerevisiae* and *S. pombe*. B. Distance to closest stop codon from alternative ATGs for highly (red) and lowly (green) expressed genes in terms of their (ribosomal density)·(mRNA levels) for *S. cerevisiae* and *S. pombe* (top/bottom 10%).
(TIF)

Figure S37 The PSSM describing the distribution of nucleotides near the main START ATG in *S. pombe* for all genes (A.), and for genes with high ribosomal-load (B.).
(TIF)

Table S1 Statistics about the genome of the analyzed organisms.
(XLSX)
