# Supplementary figures and images for: New Universal Rules of Eukaryotic Translation Initiation Fidelity
Source: PLoS Comput Biol. 2013 Jul 11;9(7):e1003136. doi: 10.1371/journal.pcbi.1003136 (PMC3708879; doi:10.1371/journal.pcbi.1003136)

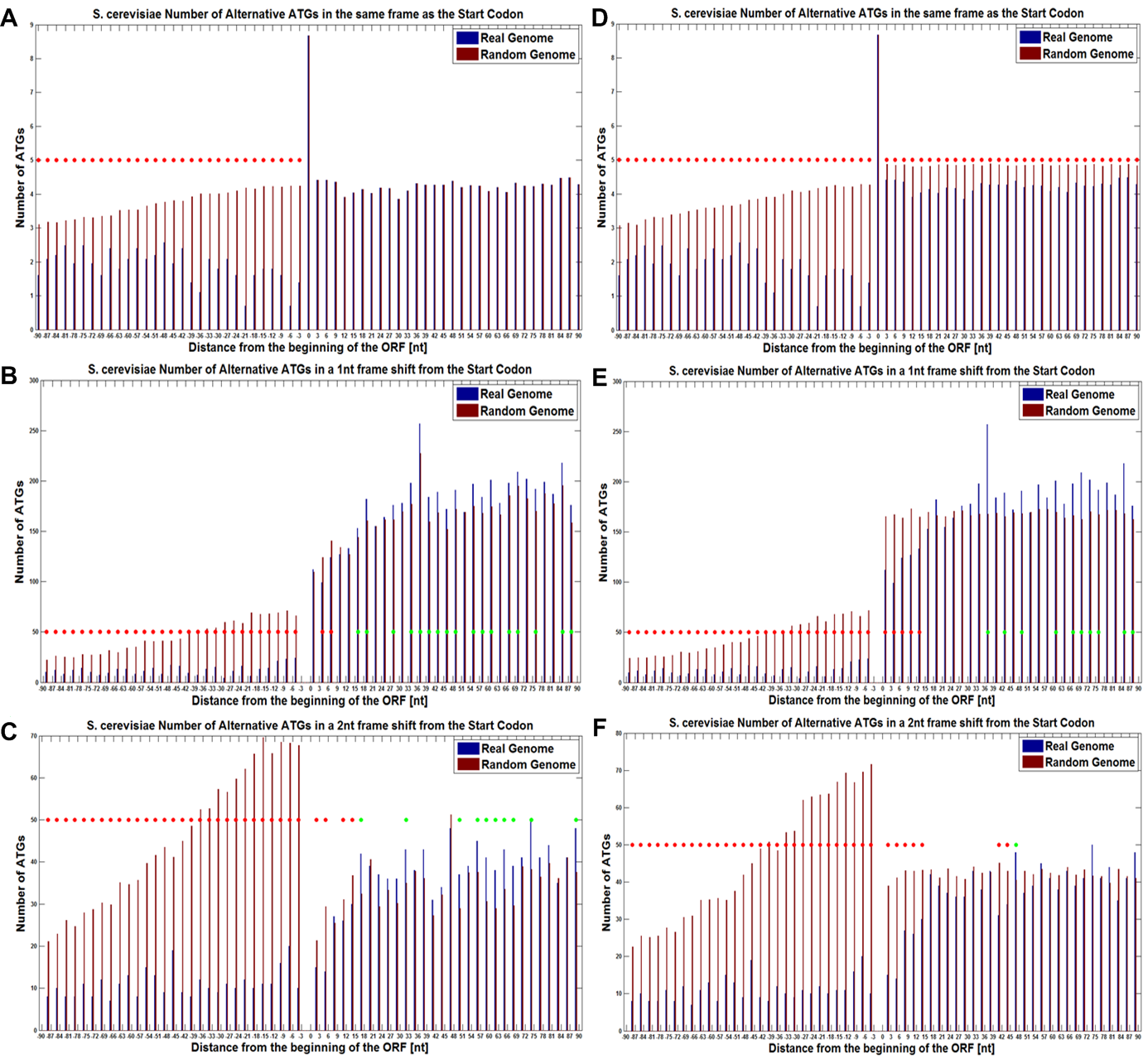

Supplement: Figure S1 — A–C. S. cerevisiae comparison of the genomic profiles of number of ATGs in the three frames to the ones obtained for randomized genomes with the same proteins, GC content, and codon bias (Methods). D–F. S. cerevisiae comparison of the genomic profiles of number of ATGs in the three frames to the ones obtained for randomized genomes that were generated by permuting the codons of each gene (Methods). (TIF) [file pcbi.1003136.s001.tif]

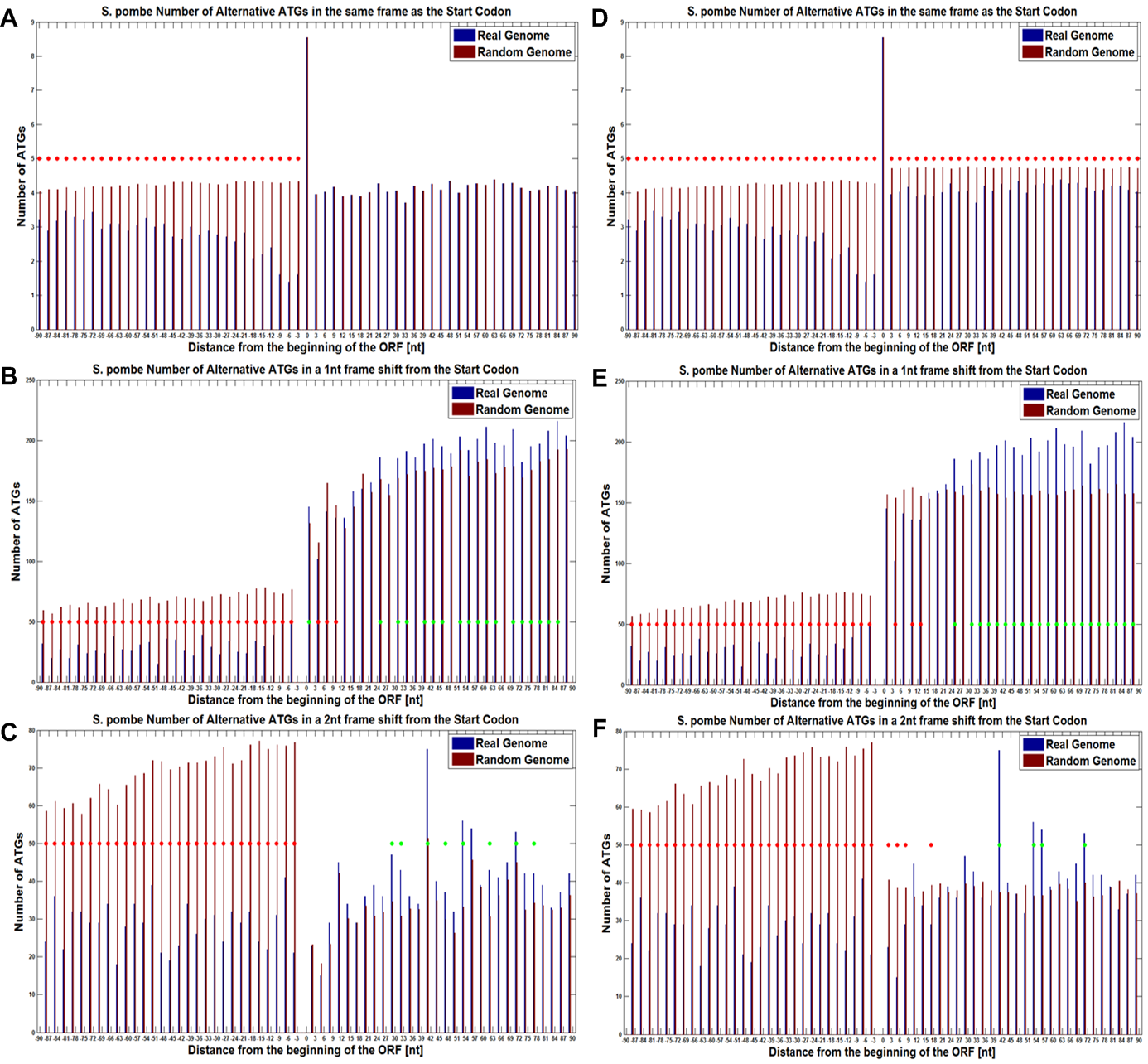

Supplement: Figure S2 — A–C. S. pombe comparison of the genomic profiles of number of ATGs in the three frames to the ones obtained for randomized genomes with the same proteins, GC content, and codon bias (Methods). D–F. S. pombe comparison of the genomic profiles of number of ATGs in the three frames to the ones obtained for randomized genomes that were generated by permuting the codons of each gene (Methods). (TIF) [file pcbi.1003136.s002.tif]

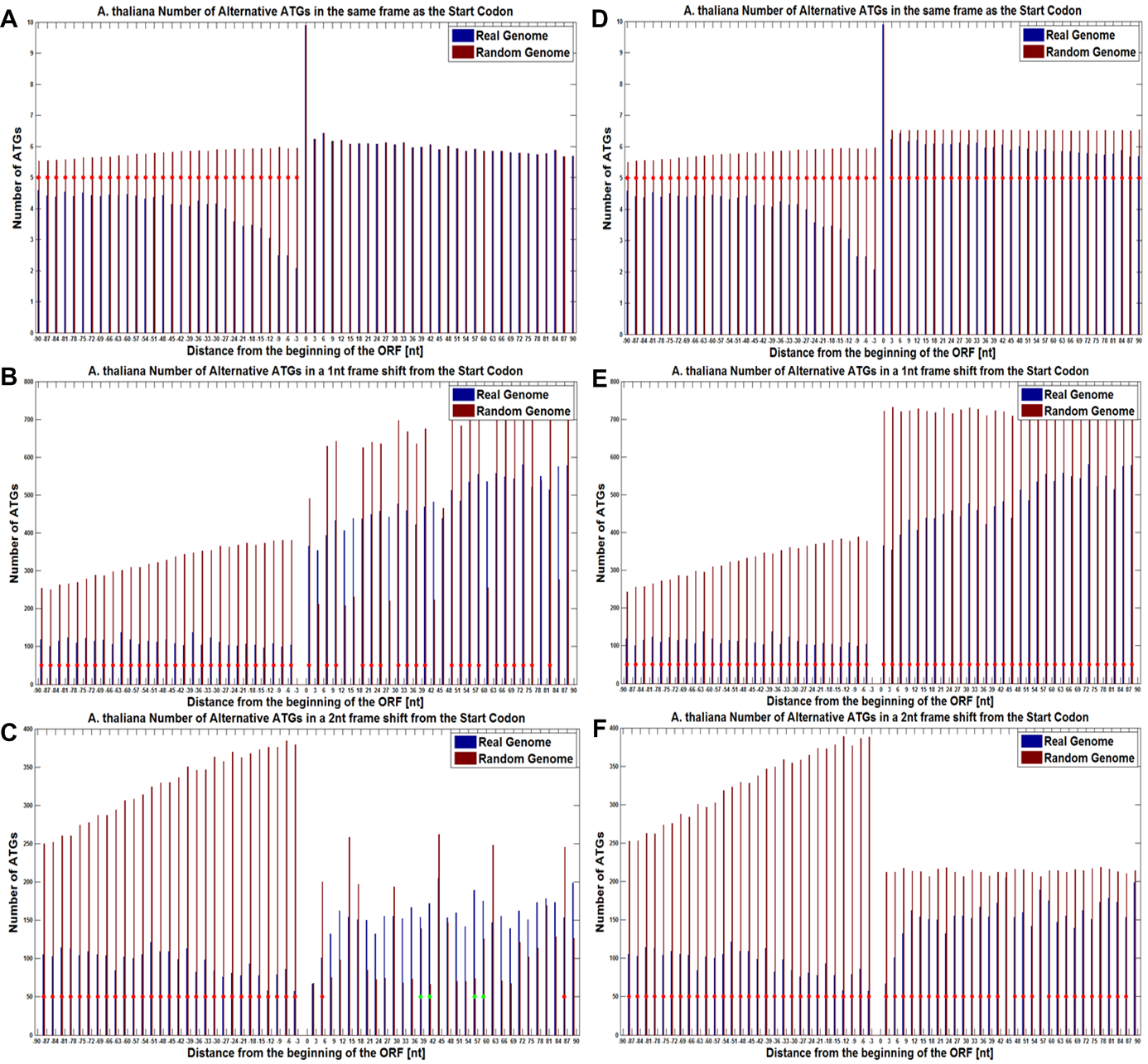

Supplement: Figure S3 — A–C. A. thaliana comparison of the genomic profiles of number of ATGs in the three frames to the ones obtained for randomized genomes with the same proteins, GC content, and codon bias (Methods). D–F. A. thaliana comparison of the genomic profiles of number of ATGs in the three frames to the ones obtained for randomized genomes that were generated by permuting the codons of each gene (Methods). (TIF) [file pcbi.1003136.s003.tif]

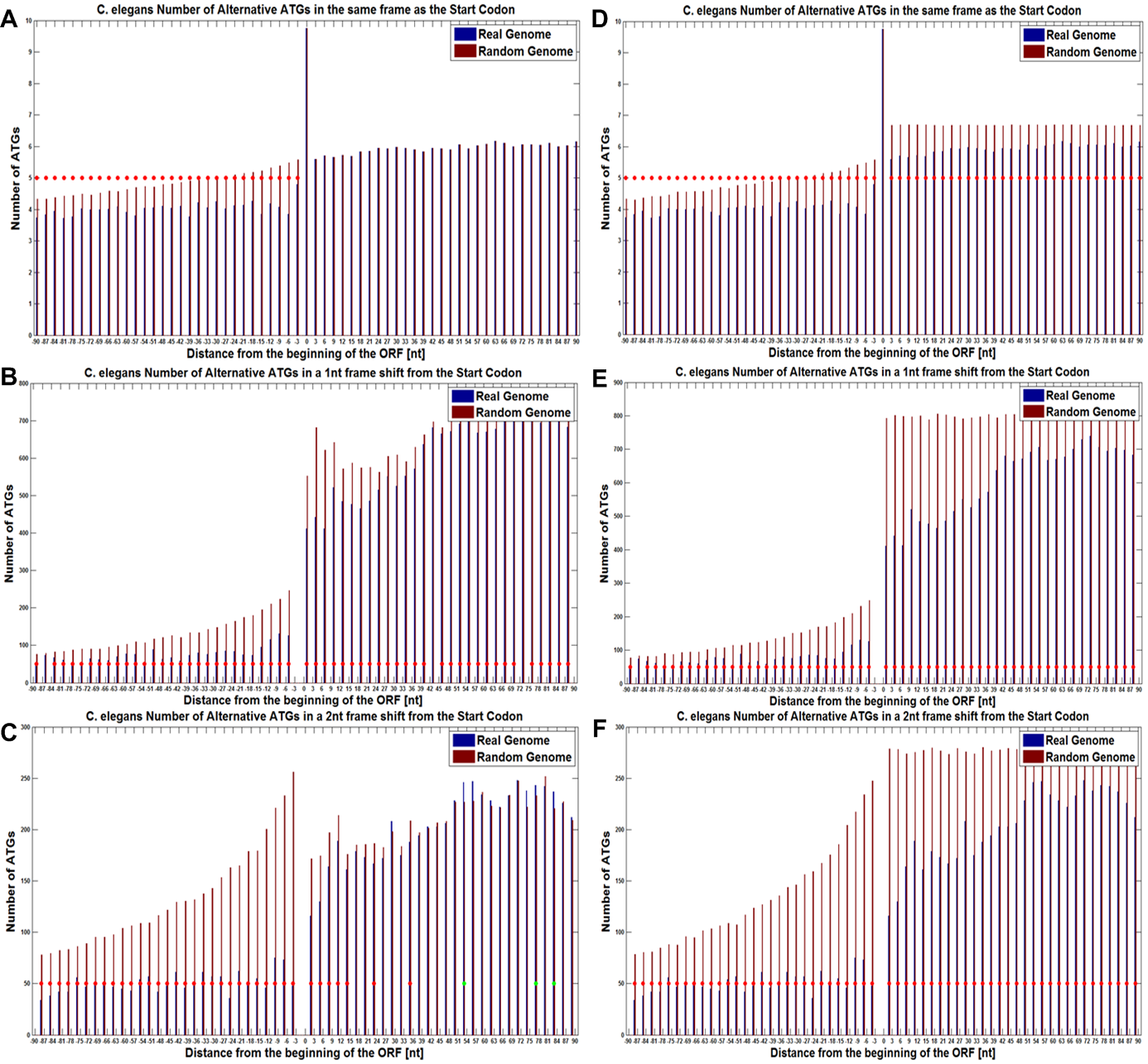

Supplement: Figure S4 — A–C. C. elegans comparison of the genomic profiles of number of ATGs in the three frames to the ones obtained for randomized genomes with the same proteins, GC content, and codon bias (Methods). D–F. C. elegans comparison of the genomic profiles of number of ATGs in the three frames to the ones obtained for randomized genomes that were generated by permuting the codons of each gene (Methods). (TIF) [file pcbi.1003136.s004.tif]

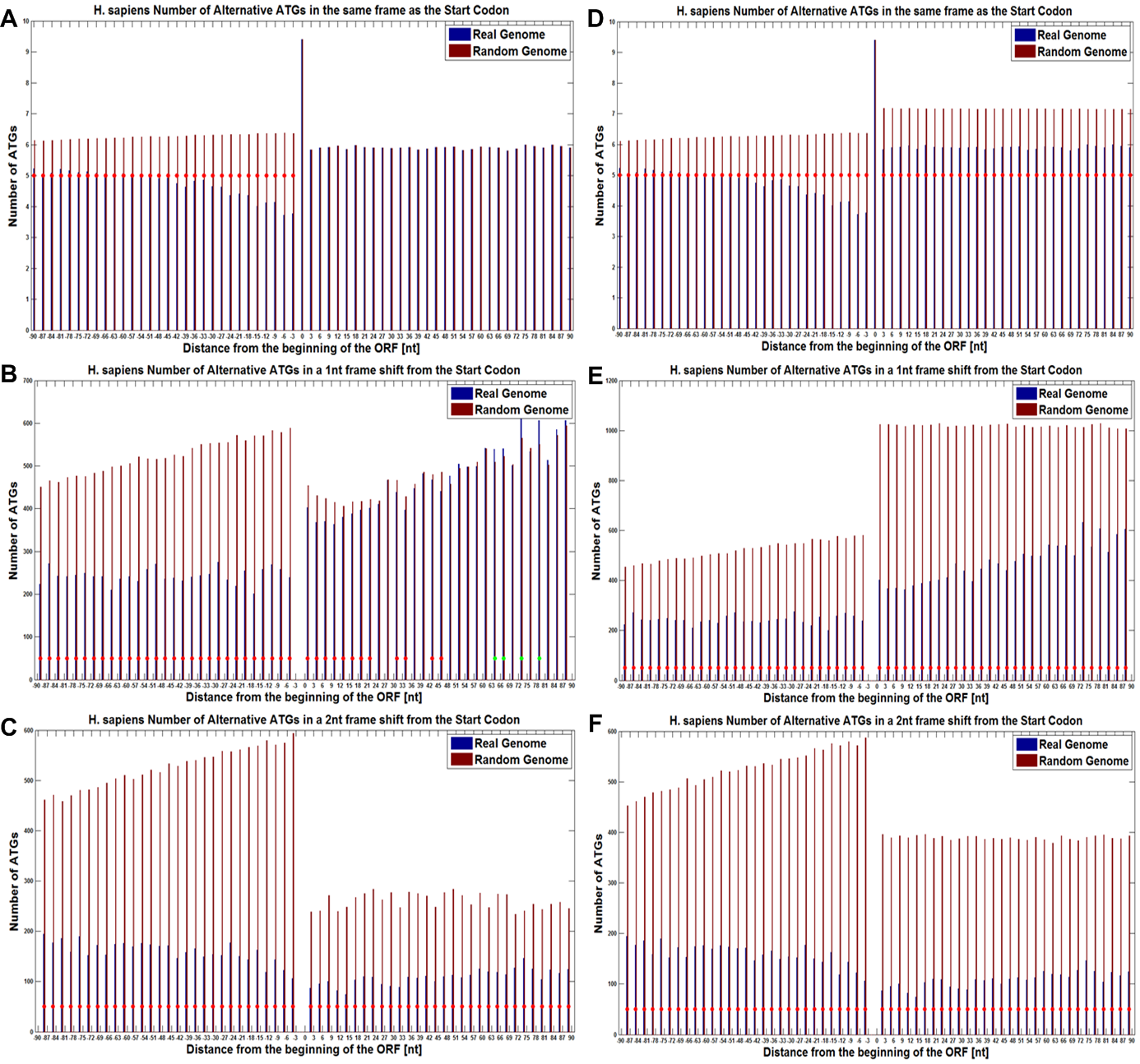

Supplement: Figure S5 — A–C. H. sapiens comparison of the genomic profiles of number of ATGs in the three frames to the ones obtained for randomized genomes with the same proteins, GC content, and codon bias (Methods). D–F. H. sapiens comparison of the genomic profiles of number of ATGs in the three frames to the ones obtained for randomized genomes that were generated by permuting the codons of each gene (Methods). (TIF) [file pcbi.1003136.s005.tif]

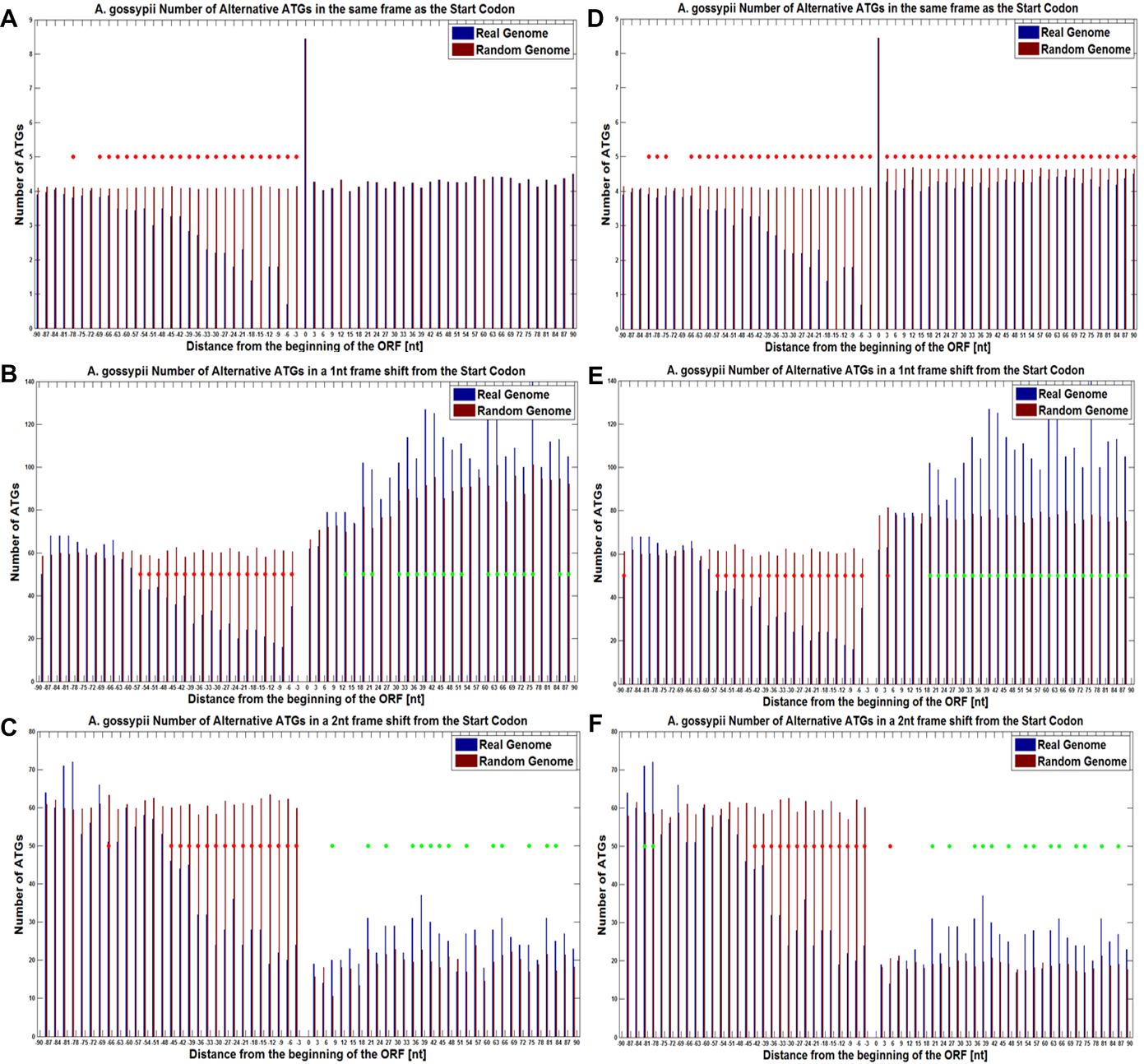

Supplement: Figure S6 — A–C. A. gossypii comparison of the genomic profiles of number of ATGs in the three frames to the ones obtained for randomized genomes with the same proteins, GC content, and codon bias (Methods). D–F. A. gossypii comparison of the genomic profiles of number of ATGs in the three frames to the ones obtained for randomized genomes that were generated by permuting the codons of each gene (Methods). (TIF) [file pcbi.1003136.s006.tif]

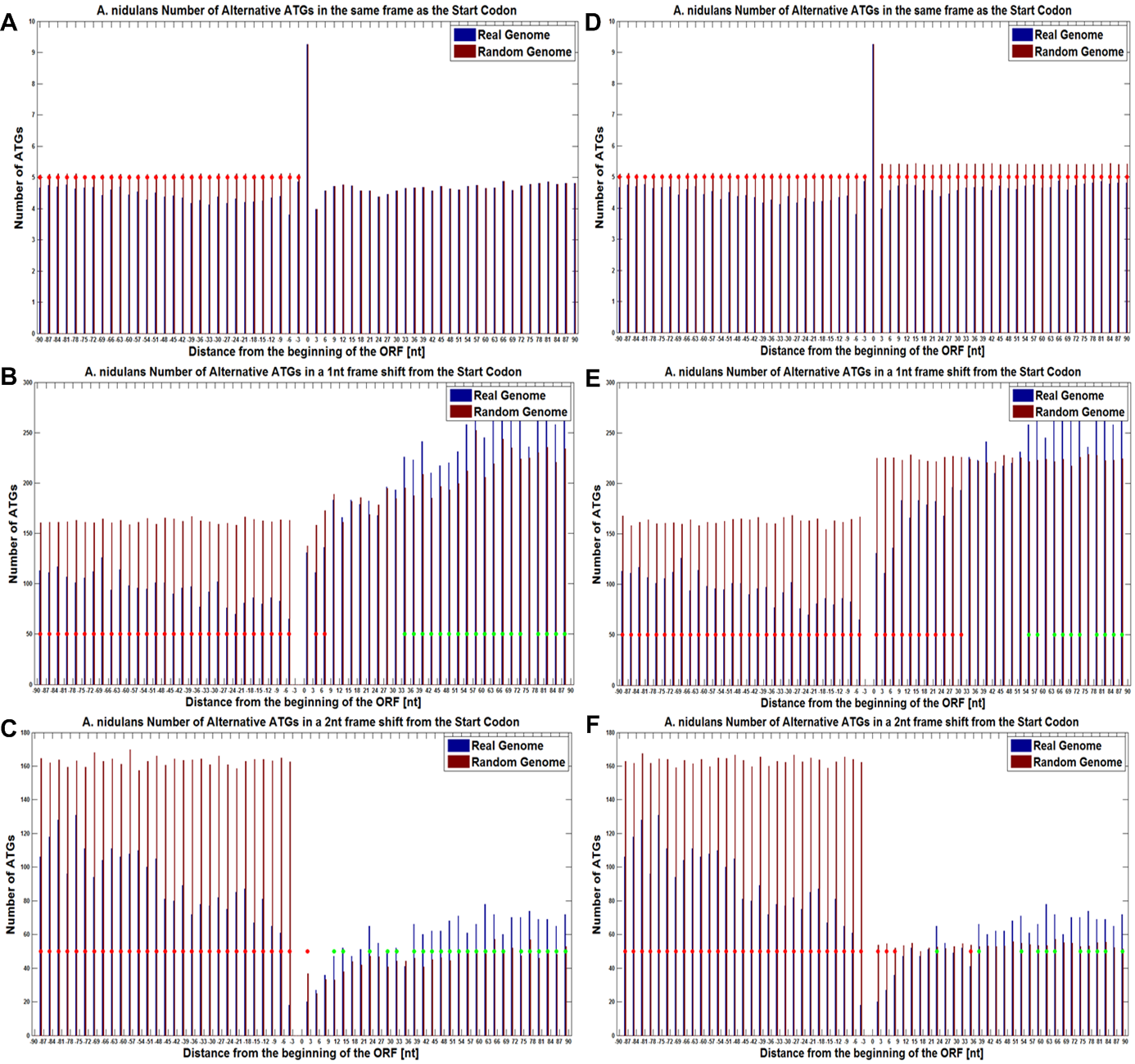

Supplement: Figure S7 — A–C. A. nidulans comparison of the genomic profiles of number of ATGs in the three frames to the ones obtained for randomized genomes with the same proteins, GC content, and codon bias (Methods). D–F. A. nidulans comparison of the genomic profiles of number of ATGs in the three frames to the ones obtained for randomized genomes that were generated by permuting the codons of each gene (Methods). (TIF) [file pcbi.1003136.s007.tif]

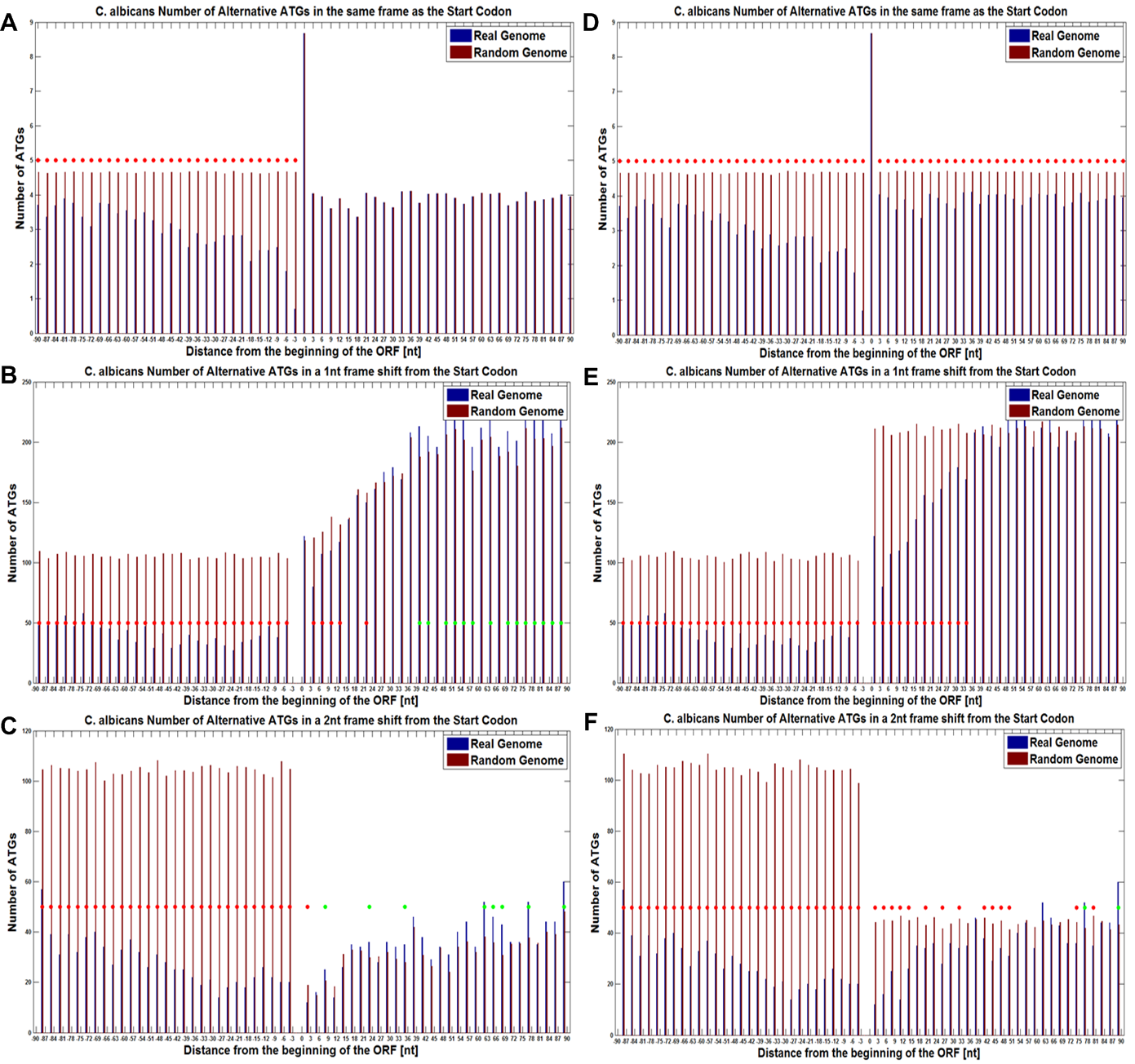

Supplement: Figure S8 — A–C. C. albicans comparison of the genomic profiles of number of ATGs in the three frames to the ones obtained for randomized genomes with the same proteins, GC content, and codon bias (Methods). D–F. C. albicans comparison of the genomic profiles of number of ATGs in the three frames to the ones obtained for randomized genomes that were generated by permuting the codons of each gene (Methods). (TIF) [file pcbi.1003136.s008.tif]

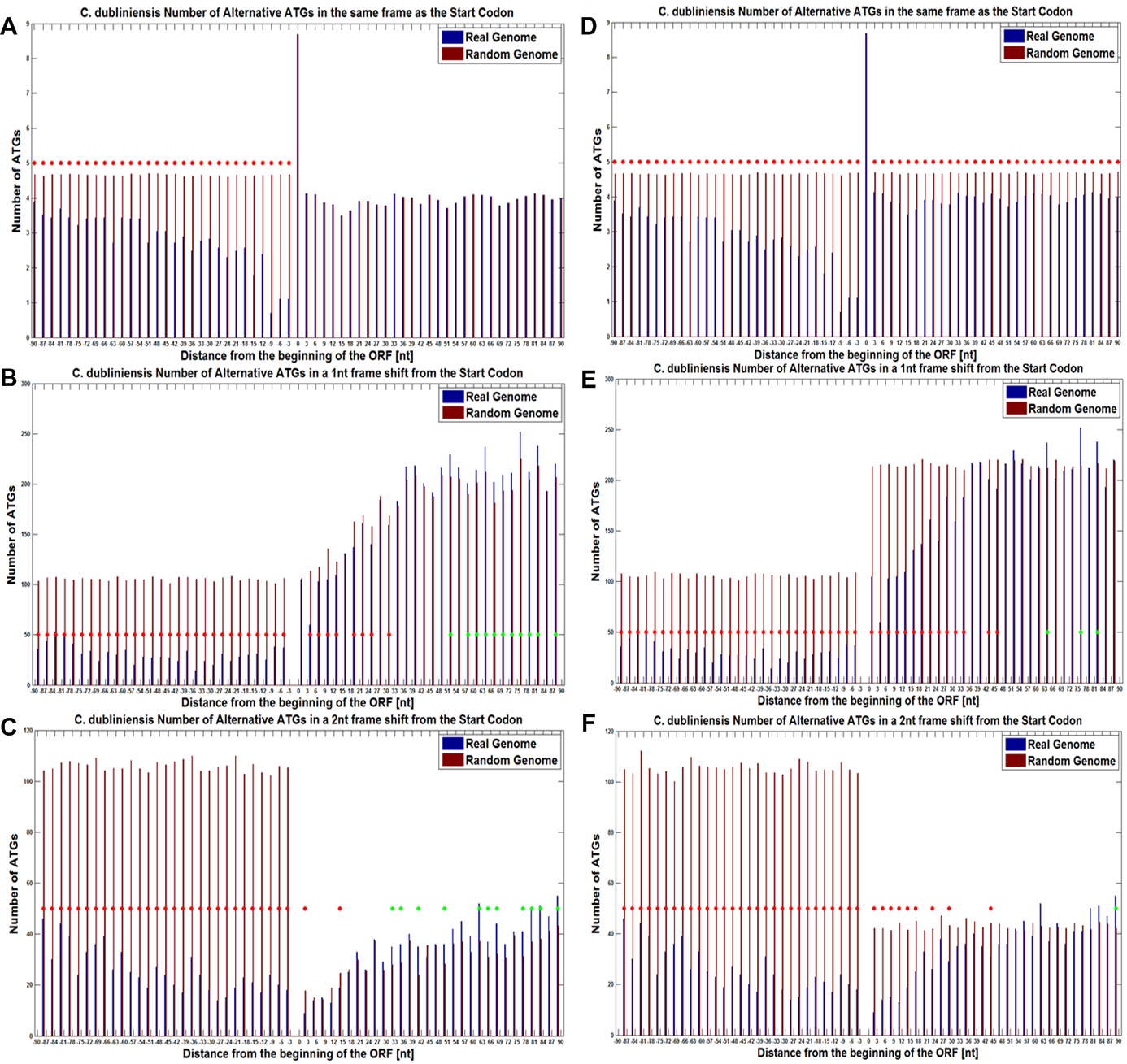

Supplement: Figure S9 — A–C. C. dubliniensis comparison of the genomic profiles of number of ATGs in the three frames to the ones obtained for randomized genomes with the same proteins, GC content, and codon bias (Methods). D–F. C. dubliniensis comparison of the genomic profiles of number of ATGs in the three frames to the ones obtained for randomized genomes that were generated by permuting the codons of each gene (Methods). (TIF) [file pcbi.1003136.s009.tif]

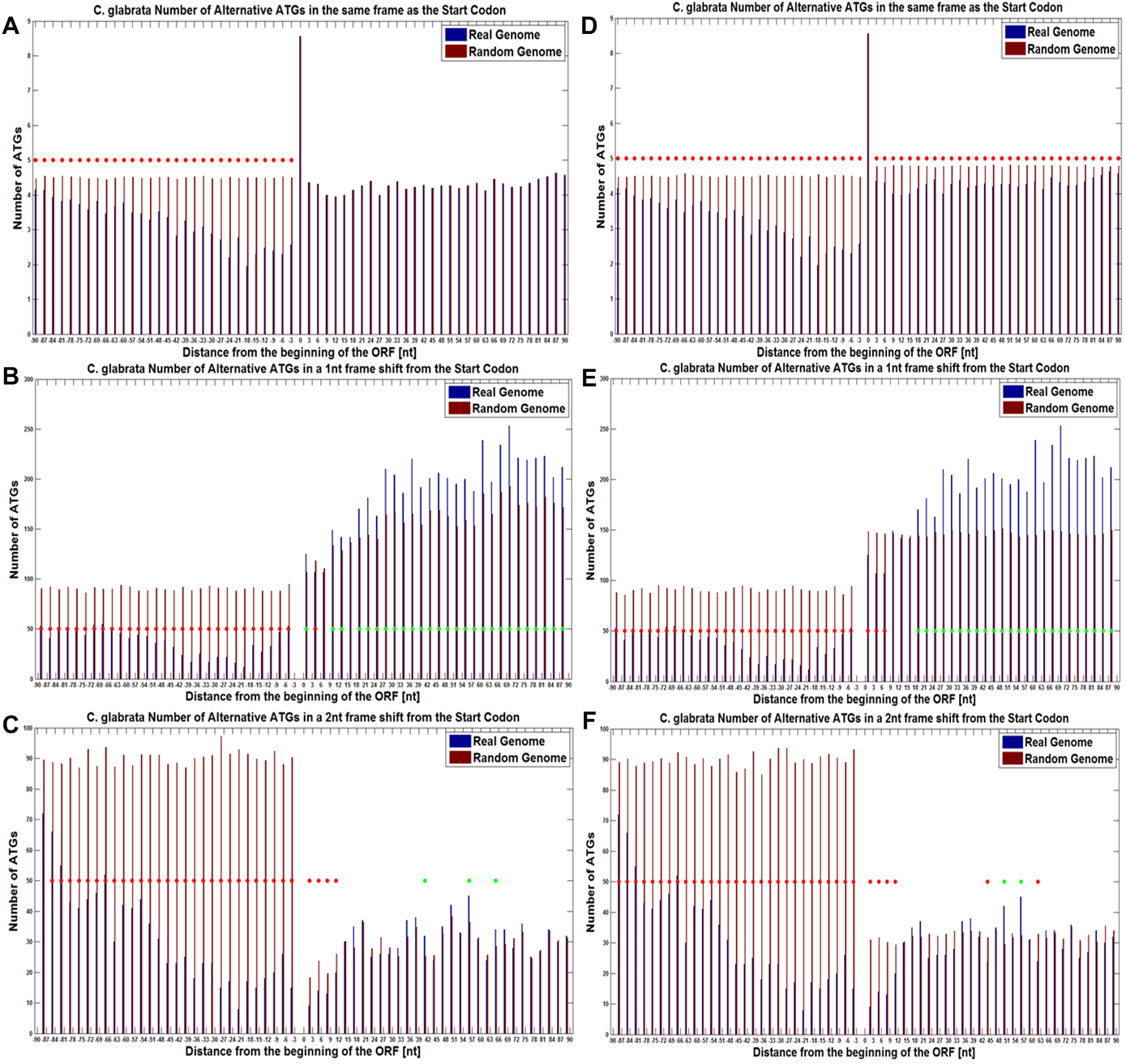

Supplement: Figure S10 — A–C. C. glabrata comparison of the genomic profiles of number of ATGs in the three frames to the ones obtained for randomized genomes with the same proteins, GC content, and codon bias (Methods). D–F. C. glabrata comparison of the genomic profiles of number of ATGs in the three frames to the ones obtained for randomized genomes that were generated by permuting the codons of each gene (Methods). (TIF) [file pcbi.1003136.s010.tif]

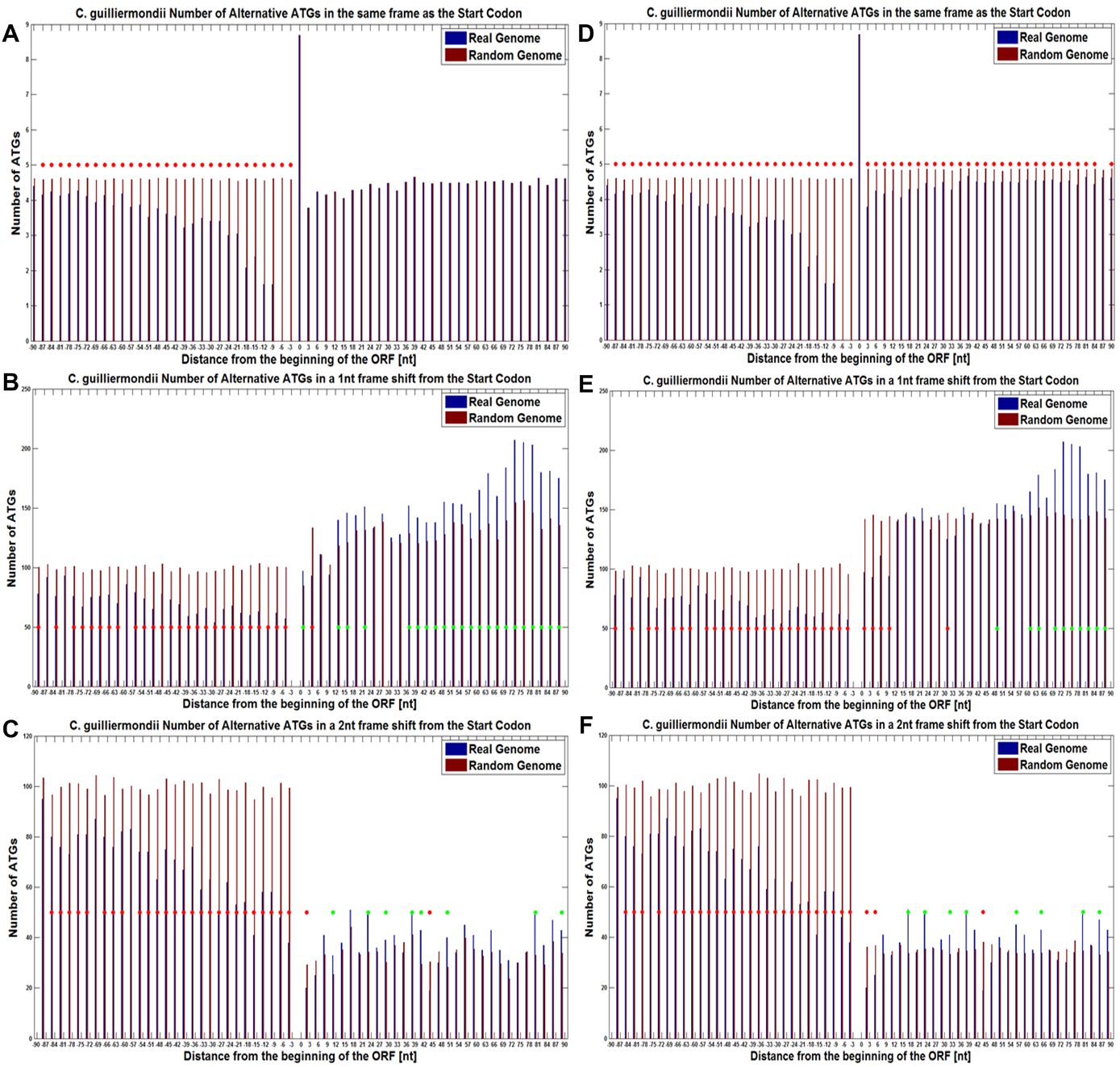

Supplement: Figure S11 — A–C. C. guilliermondii comparison of the genomic profiles of number of ATGs in the three frames to the ones obtained for randomized genomes with the same proteins, GC content, and codon bias (Methods). D–F. C. guilliermondii comparison of the genomic profiles of number of ATGs in the three frames to the ones obtained for randomized genomes that were generated by permuting the codons of each gene (Methods). (TIF) [file pcbi.1003136.s011.tif]

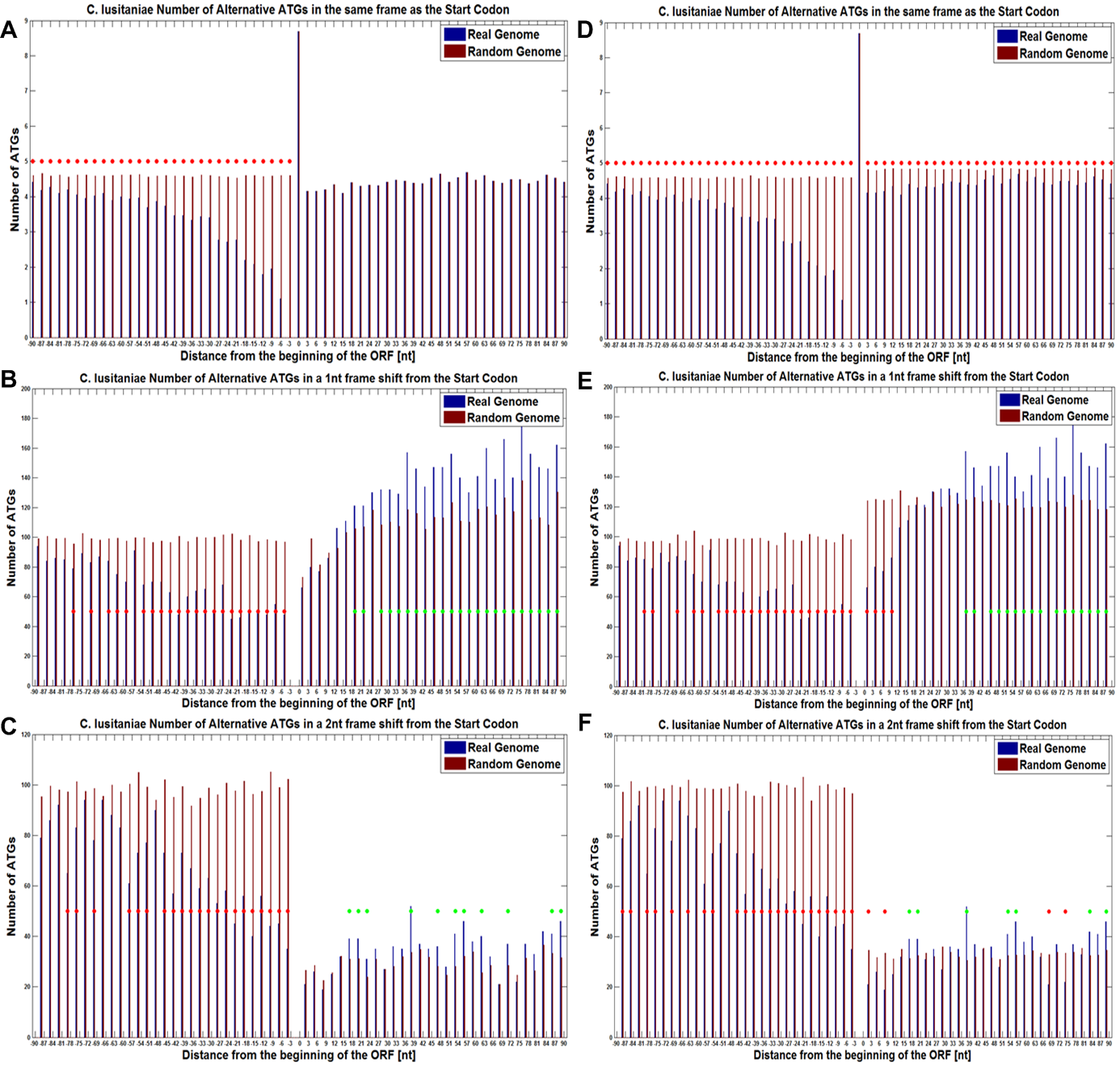

Supplement: Figure S12 — A–C. C. lusitaniae comparison of the genomic profiles of number of ATGs in the three frames to the ones obtained for randomized genomes with the same proteins, GC content, and codon bias (Methods). D–F. C. lusitaniae comparison of the genomic profiles of number of ATGs in the three frames to the ones obtained for randomized genomes that were generated by permuting the codons of each gene (Methods). (TIF) [file pcbi.1003136.s012.tif]

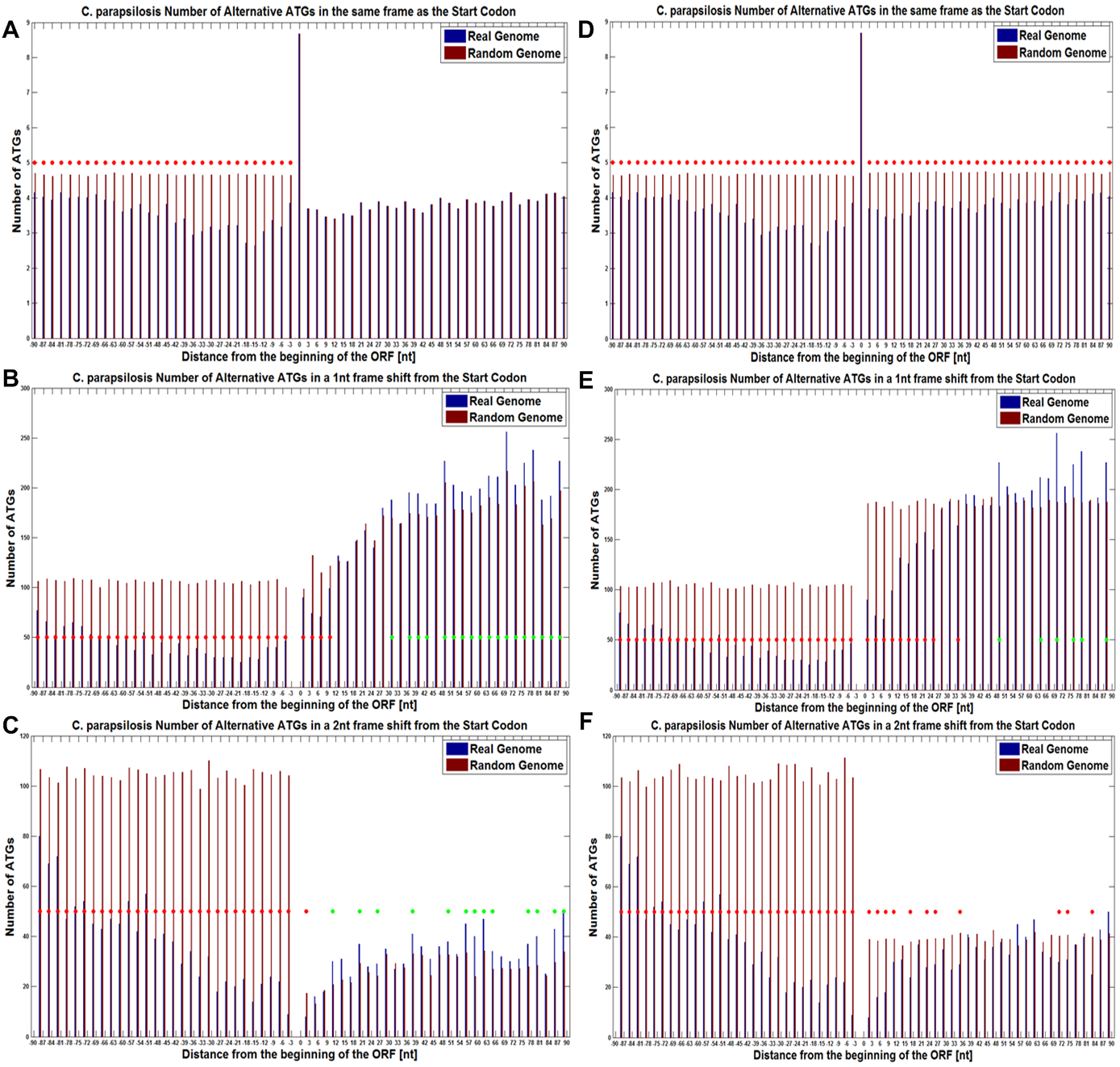

Supplement: Figure S13 — A–C. C. parapsilosis comparison of the genomic profiles of number of ATGs in the three frames to the ones obtained for randomized genomes with the same proteins, GC content, and codon bias (Methods). D–F. C. parapsilosis comparison of the genomic profiles of number of ATGs in the three frames to the ones obtained for randomized genomes that were generated by permuting the codons of each gene (Methods). (TIF) [file pcbi.1003136.s013.tif]

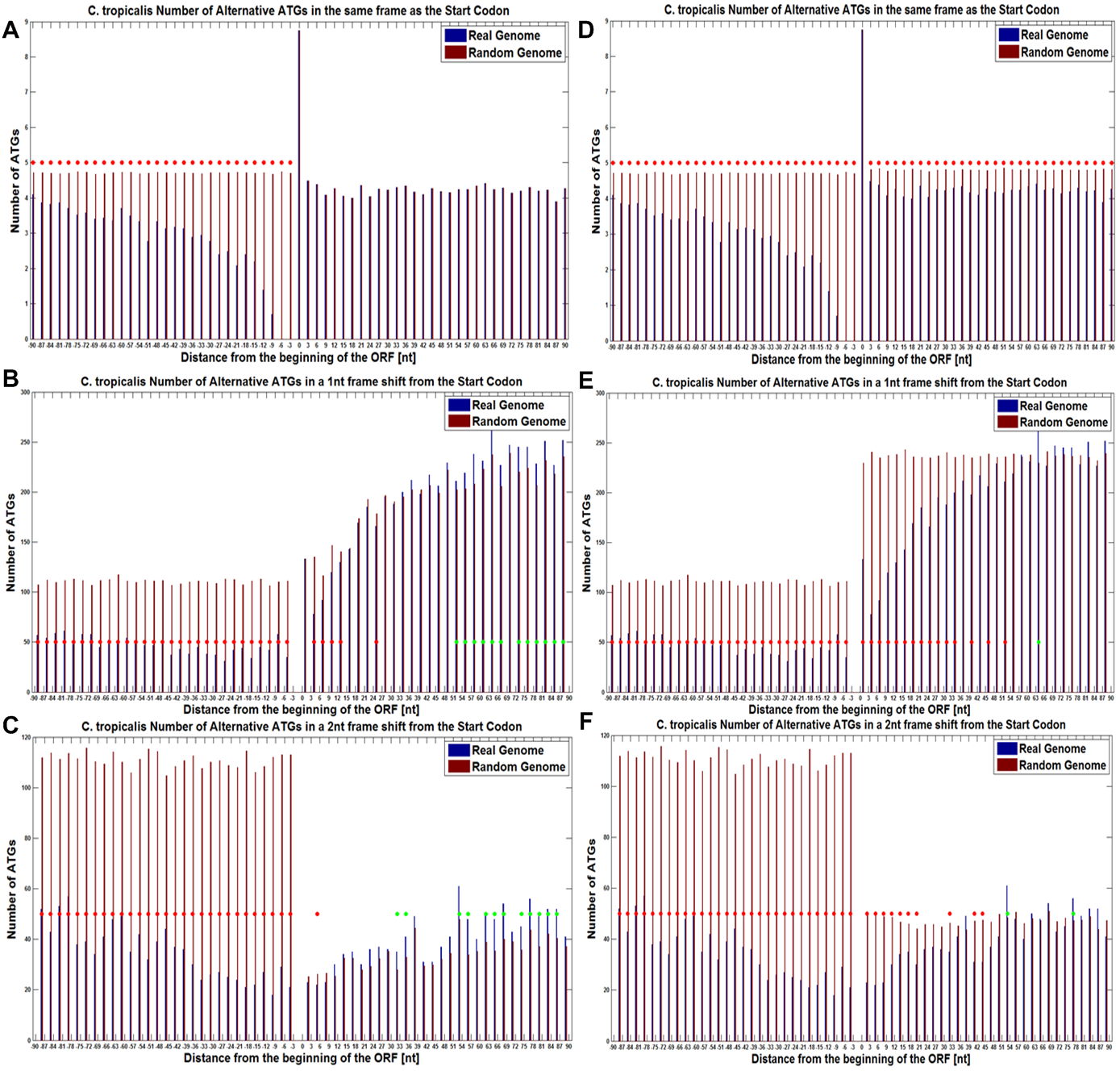

Supplement: Figure S14 — A–C. C. tropicalis comparison of the genomic profiles of number of ATGs in the three frames to the ones obtained for randomized genomes with the same proteins, GC content, and codon bias (Methods). D–F. C. tropicalis comparison of the genomic profiles of number of ATGs in the three frames to the ones obtained for randomized genomes that were generated by permuting the codons of each gene (Methods). (TIF) [file pcbi.1003136.s014.tif]

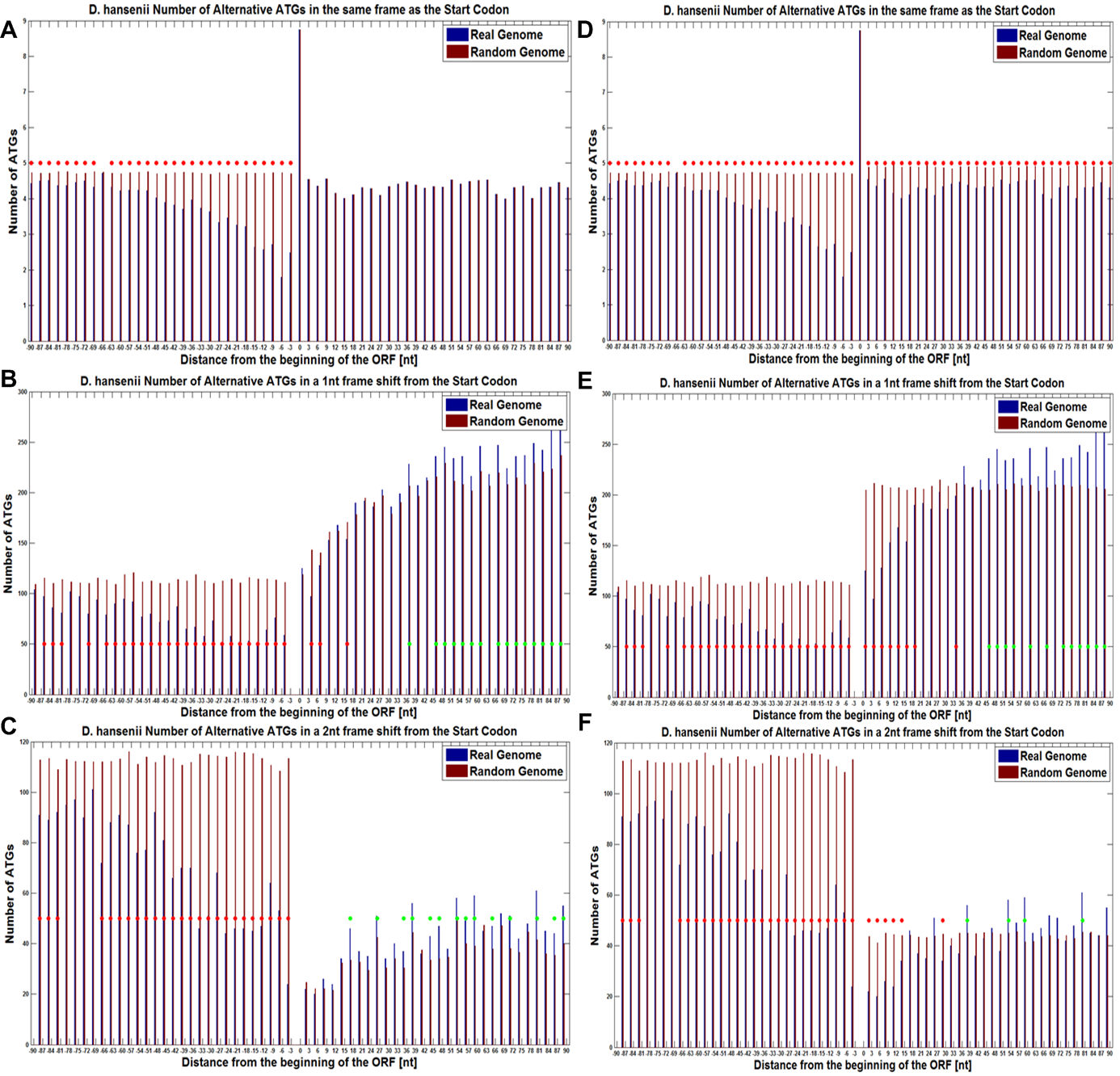

Supplement: Figure S15 — A–C. D. hansenii comparison of the genomic profiles of number of ATGs in the three frames to the ones obtained for randomized genomes with the same proteins, GC content, and codon bias (Methods). D–F. D. hansenii comparison of the genomic profiles of number of ATGs in the three frames to the ones obtained for randomized genomes that were generated by permuting the codons of each gene (Methods). (TIF) [file pcbi.1003136.s015.tif]

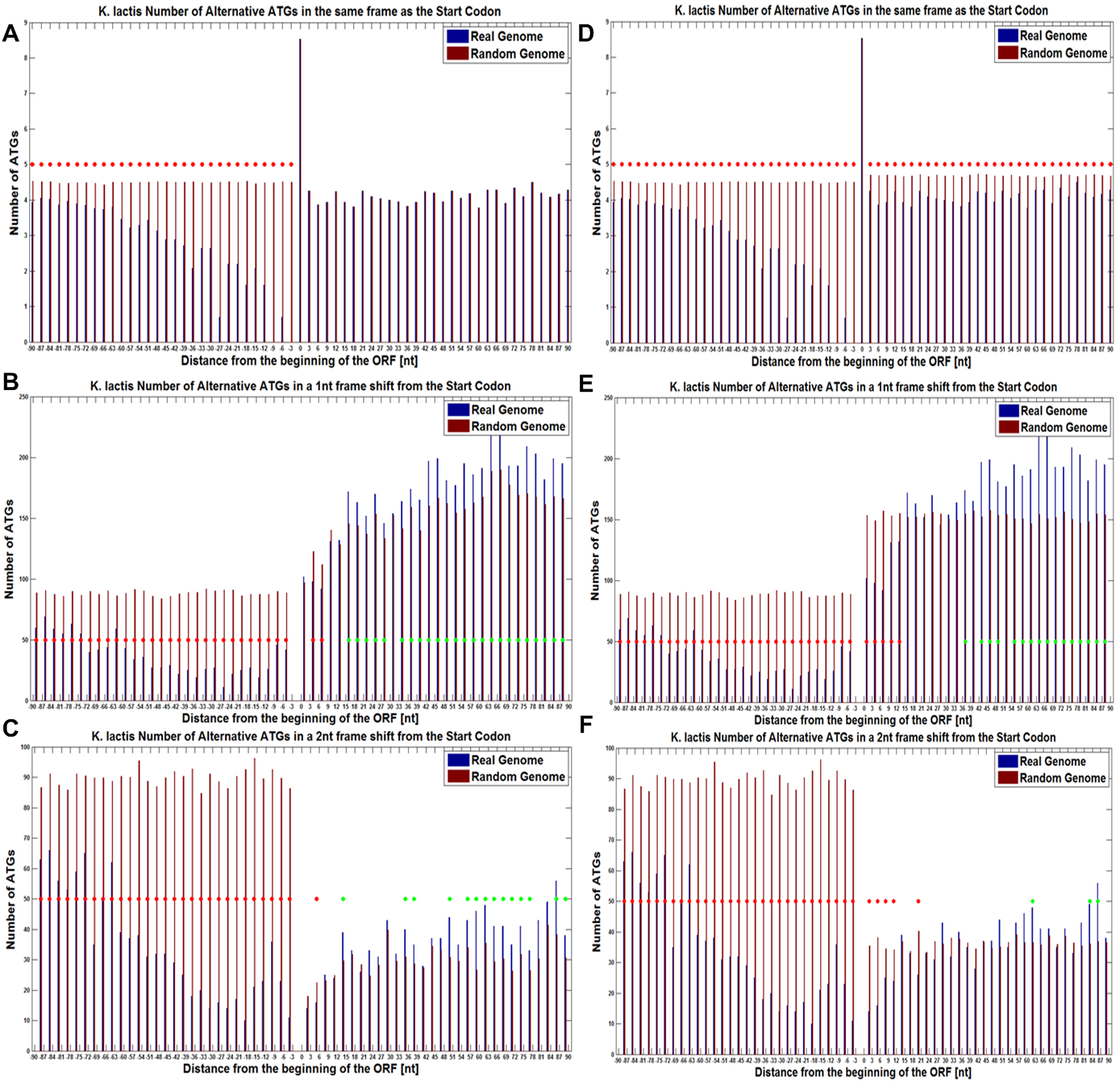

Supplement: Figure S16 — A–C. K. lactis comparison of the genomic profiles of number of ATGs in the three frames to the ones obtained for randomized genomes with the same proteins, GC content, and codon bias (Methods). D–F. K. lactis comparison of the genomic profiles of number of ATGs in the three frames to the ones obtained for randomized genomes that were generated by permuting the codons of each gene (Methods). (TIF) [file pcbi.1003136.s016.tif]

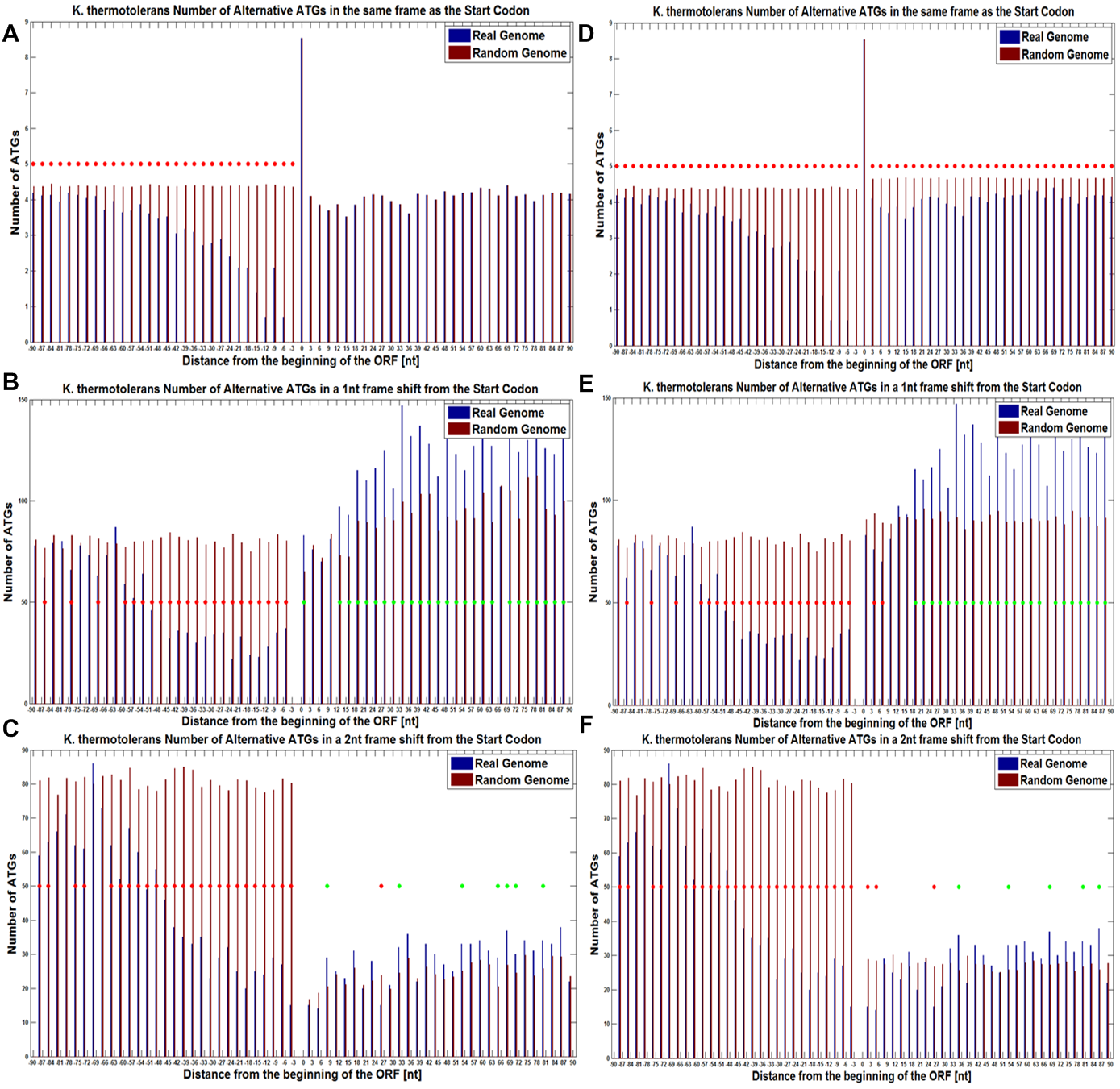

Supplement: Figure S17 — A–C. K. thermotolerans comparison of the genomic profiles of number of ATGs in the three frames to the ones obtained for randomized genomes with the same proteins, GC content, and codon bias (Methods). D–F. K. thermotolerans comparison of the genomic profiles of number of ATGs in the three frames to the ones obtained for randomized genomes that were generated by permuting the codons of each gene (Methods). (TIF) [file pcbi.1003136.s017.tif]

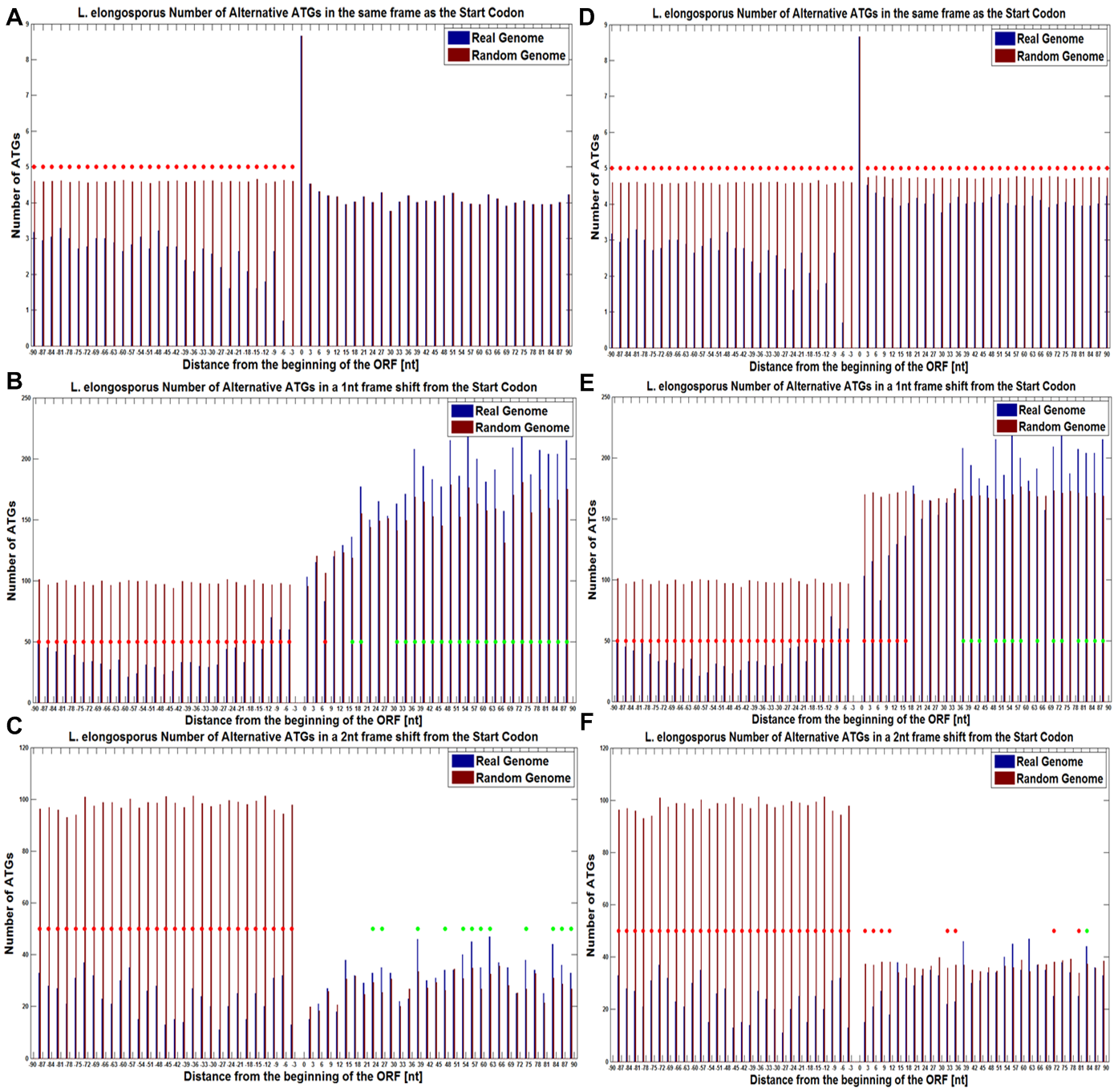

Supplement: Figure S18 — A–C. L. elongosporus comparison of the genomic profiles of number of ATGs in the three frames to the ones obtained for randomized genomes with the same proteins, GC content, and codon bias (Methods). D–F. L. elongosporus comparison of the genomic profiles of number of ATGs in the three frames to the ones obtained for randomized genomes that were generated by permuting the codons of each gene (Methods). (TIF) [file pcbi.1003136.s018.tif]

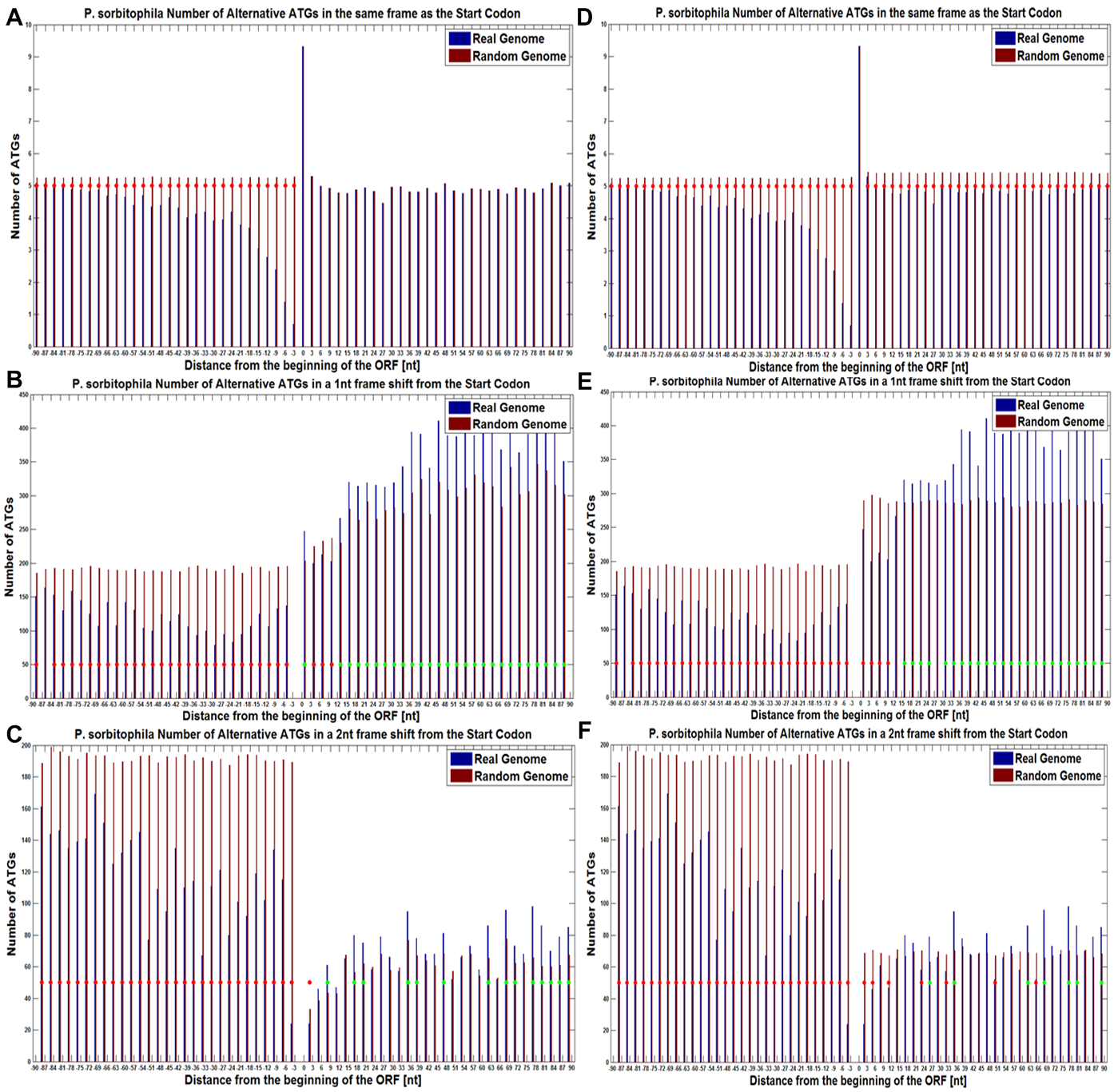

Supplement: Figure S19 — A–C. P. sorbitophila comparison of the genomic profiles of number of ATGs in the three frames to the ones obtained for randomized genomes with the same proteins, GC content, and codon bias (Methods). D–F. P. sorbitophila comparison of the genomic profiles of number of ATGs in the three frames to the ones obtained for randomized genomes that were generated by permuting the codons of each gene (Methods). (TIF) [file pcbi.1003136.s019.tif]

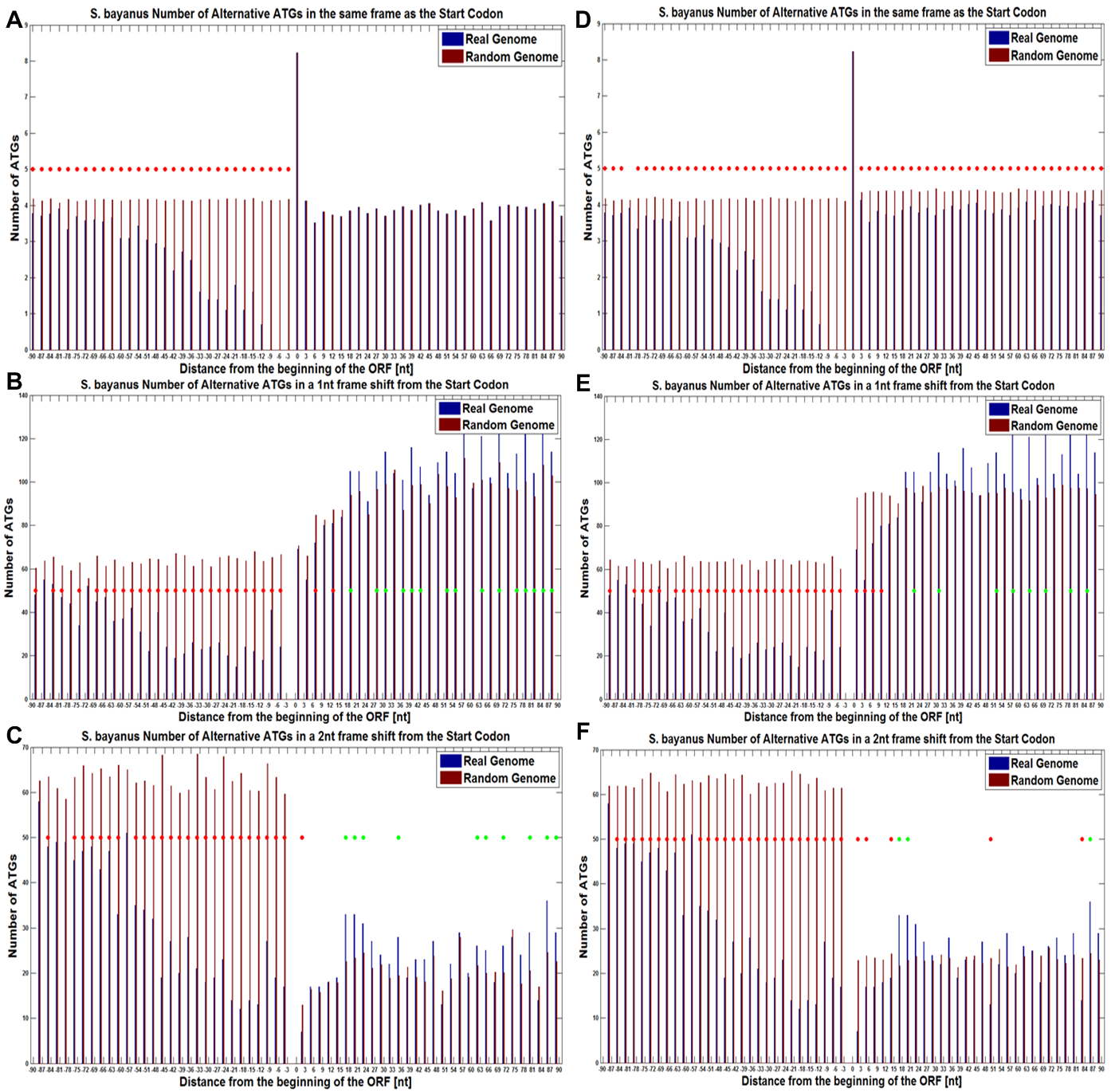

Supplement: Figure S20 — A–C. S. bayanus comparison of the genomic profiles of number of ATGs in the three frames to the ones obtained for randomized genomes with the same proteins, GC content, and codon bias (Methods). D–F. S. bayanus comparison of the genomic profiles of number of ATGs in the three frames to the ones obtained for randomized genomes that were generated by permuting the codons of each gene (Methods). (TIF) [file pcbi.1003136.s020.tif]

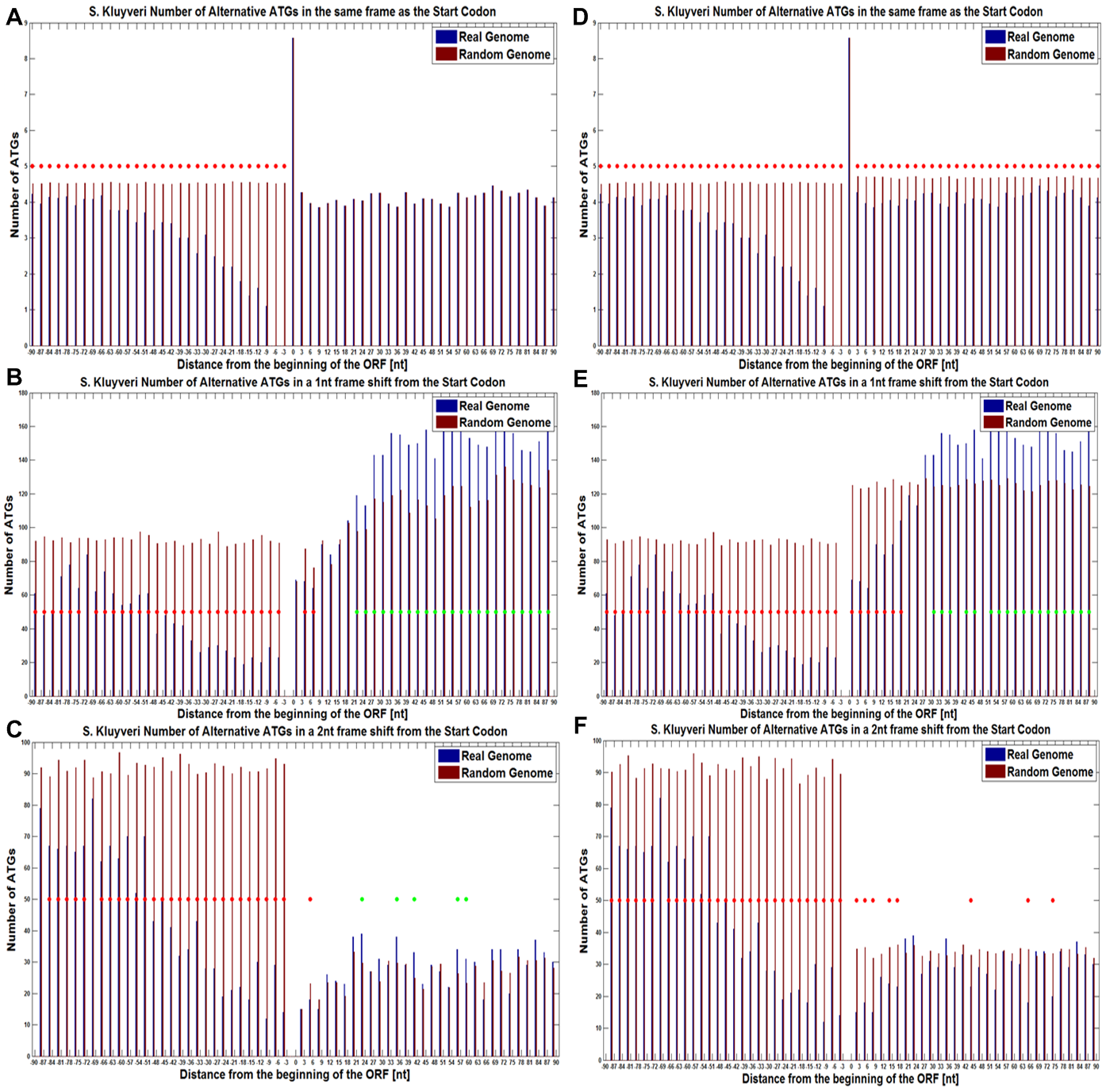

Supplement: Figure S21 — A–C. S. Kluyveri comparison of the genomic profiles of number of ATGs in the three frames to the ones obtained for randomized genomes with the same proteins, GC content, and codon bias (Methods). D–F. S. Kluyveri comparison of the genomic profiles of number of ATGs in the three frames to the ones obtained for randomized genomes that were generated by permuting the codons of each gene (Methods). (TIF) [file pcbi.1003136.s021.tif]

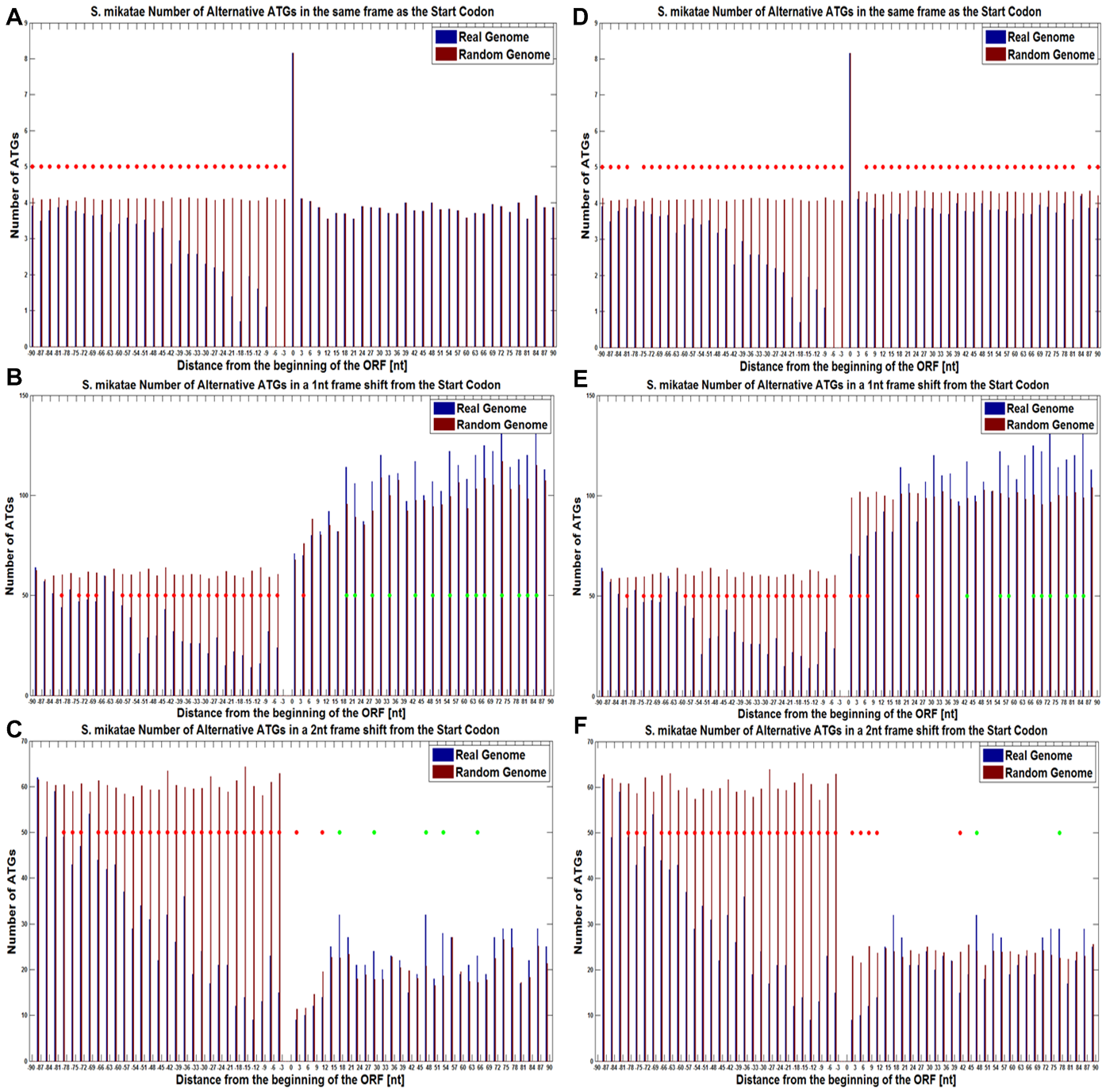

Supplement: Figure S22 — A–C. S. mikatae comparison of the genomic profiles of number of ATGs in the three frames to the ones obtained for randomized genomes with the same proteins, GC content, and codon bias (Methods). D–F. S. mikatae comparison of the genomic profiles of number of ATGs in the three frames to the ones obtained for randomized genomes that were generated by permuting the codons of each gene (Methods). (TIF) [file pcbi.1003136.s022.tif]

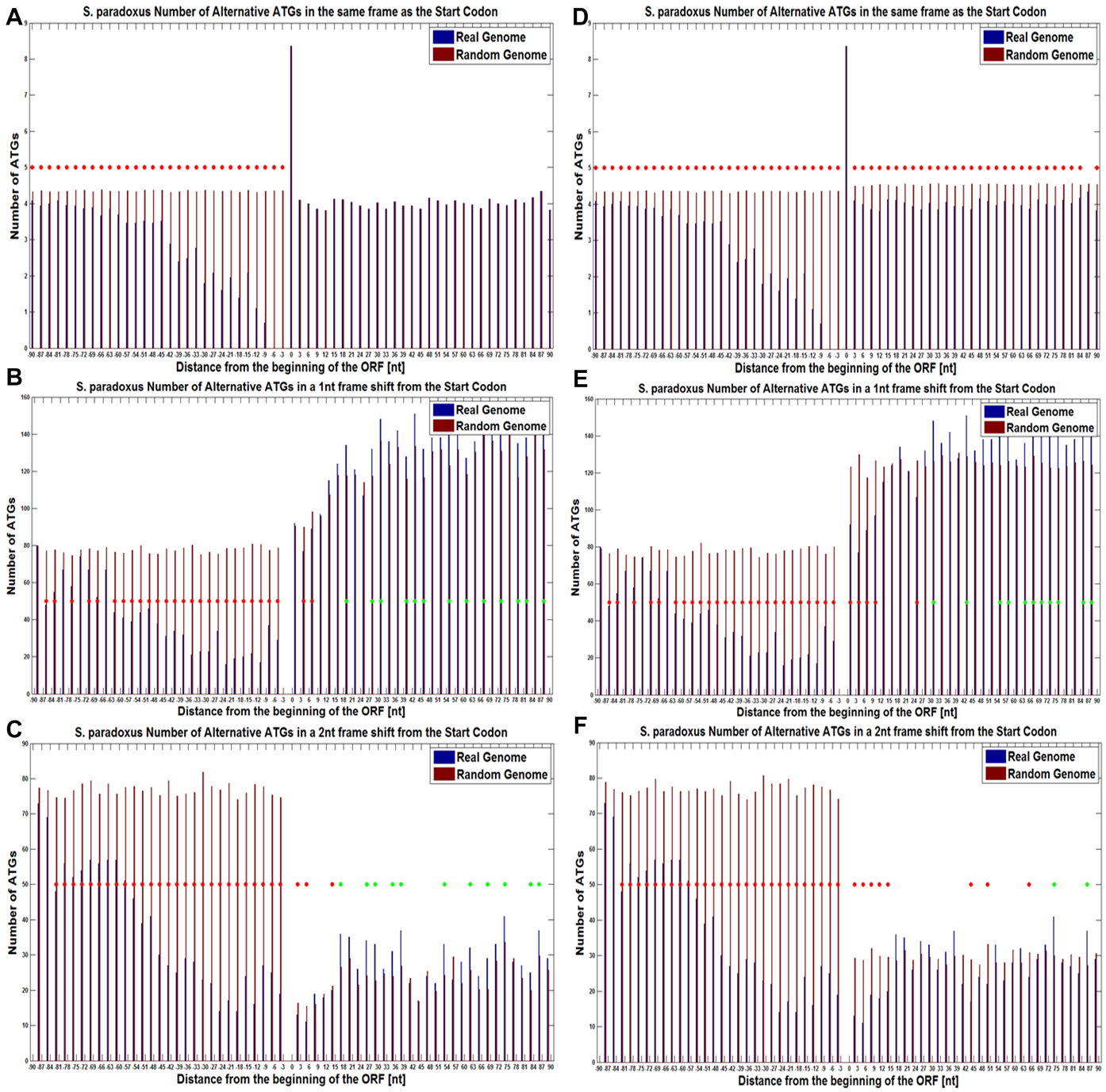

Supplement: Figure S23 — A–C. S. paradoxus comparison of the genomic profiles of number of ATGs in the three frames to the ones obtained for randomized genomes with the same proteins, GC content, and codon bias (Methods). D–F. S. paradoxus comparison of the genomic profiles of number of ATGs in the three frames to the ones obtained for randomized genomes that were generated by permuting the codons of each gene (Methods). (TIF) [file pcbi.1003136.s023.tif]

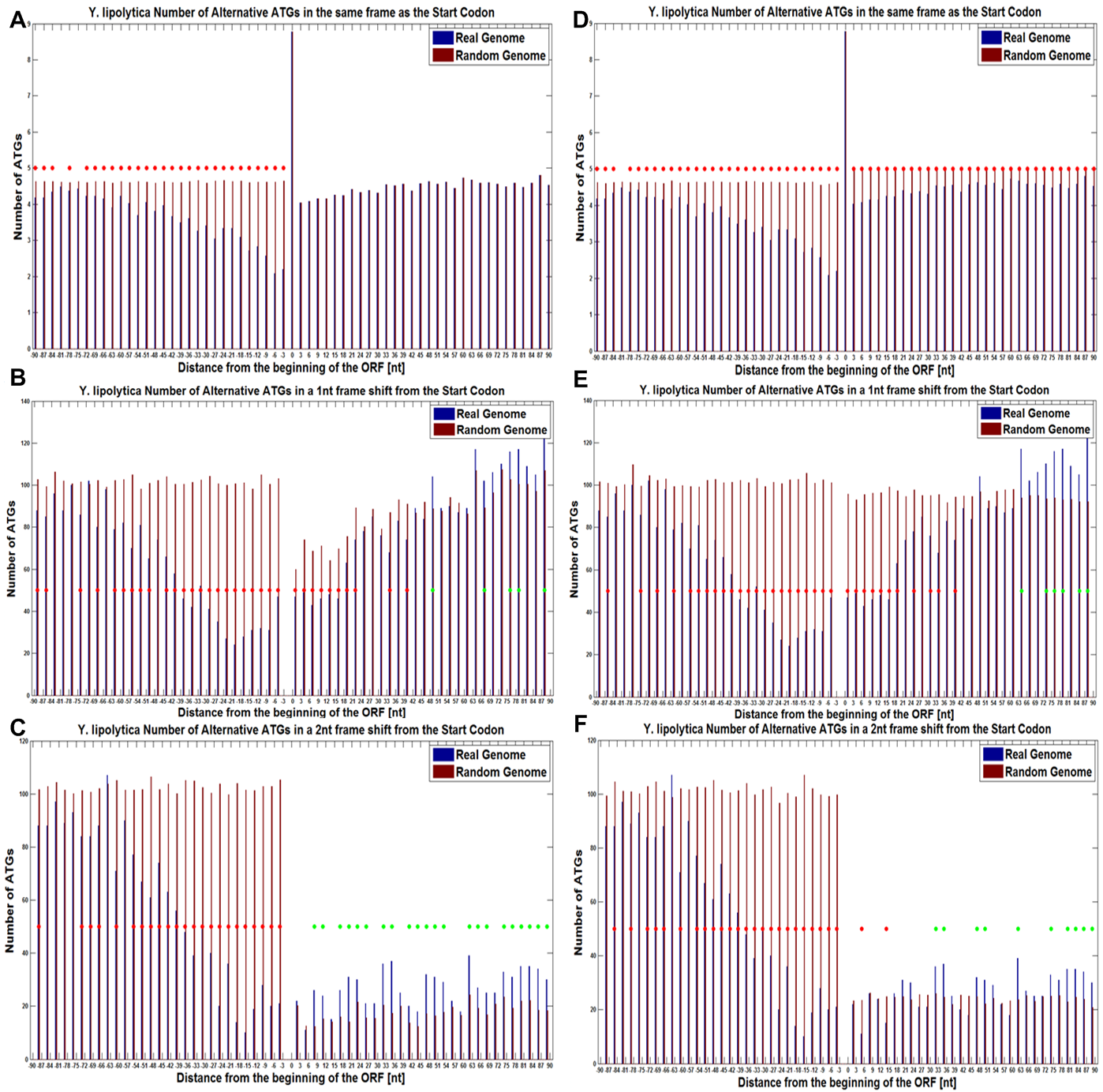

Supplement: Figure S24 — A–C. Y. lipolytica comparison of the genomic profiles of number of ATGs in the three frames to the ones obtained for randomized genomes with the same proteins, GC content, and codon bias (Methods). D–F. Y. lipolytica comparison of the genomic profiles of number of ATGs in the three frames to the ones obtained for randomized genomes that were generated by permuting the codons of each gene (Methods). (TIF) [file pcbi.1003136.s024.tif]

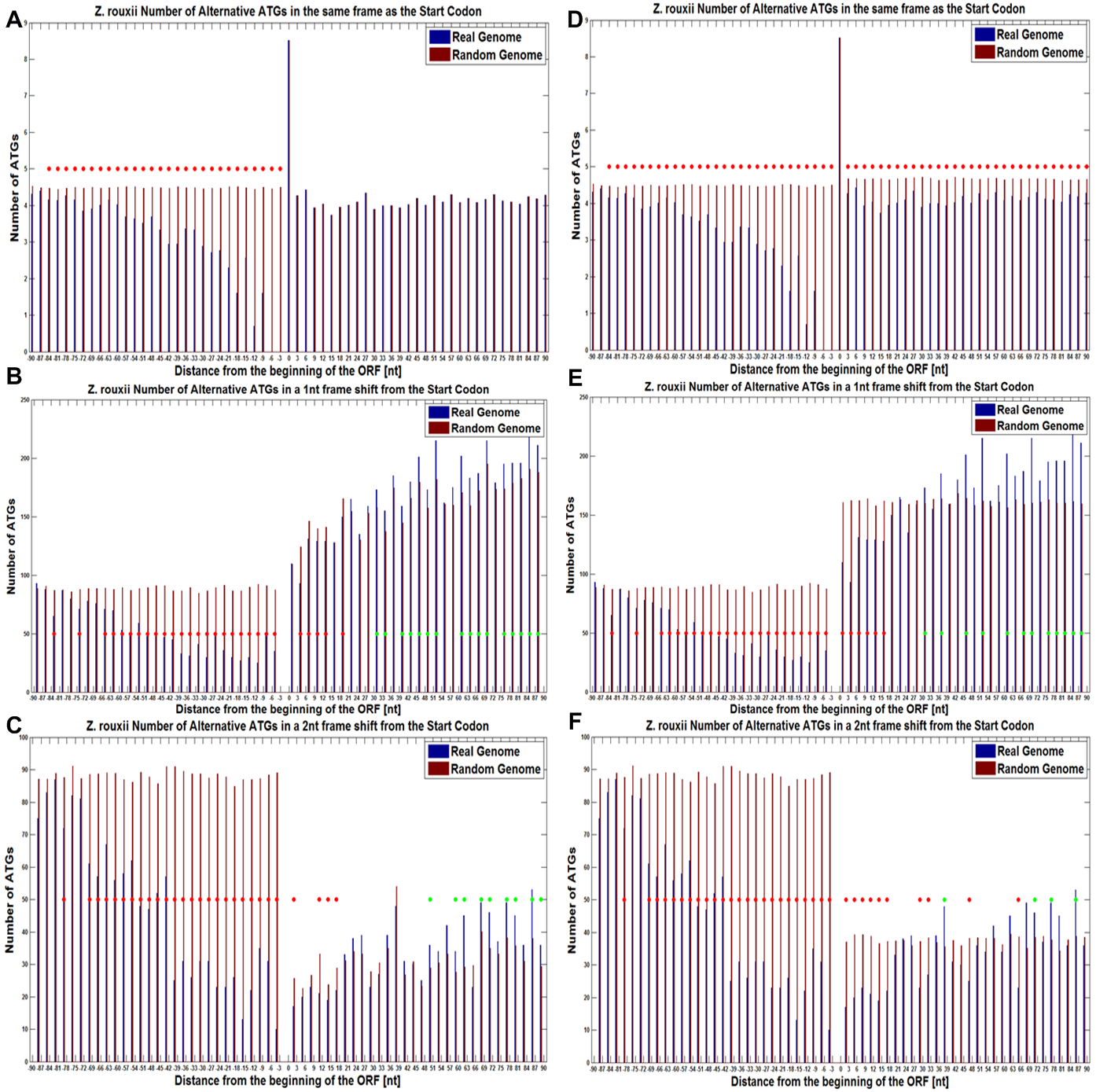

Supplement: Figure S25 — A–C. Z. rouxii comparison of the genomic profiles of number of ATGs in the three frames to the ones obtained for randomized genomes with the same proteins, GC content, and codon bias (Methods). D–F. Z. rouxii comparison of the genomic profiles of number of ATGs in the three frames to the ones obtained for randomized genomes that were generated by permuting the codons of each gene (Methods). (TIF) [file pcbi.1003136.s025.tif]

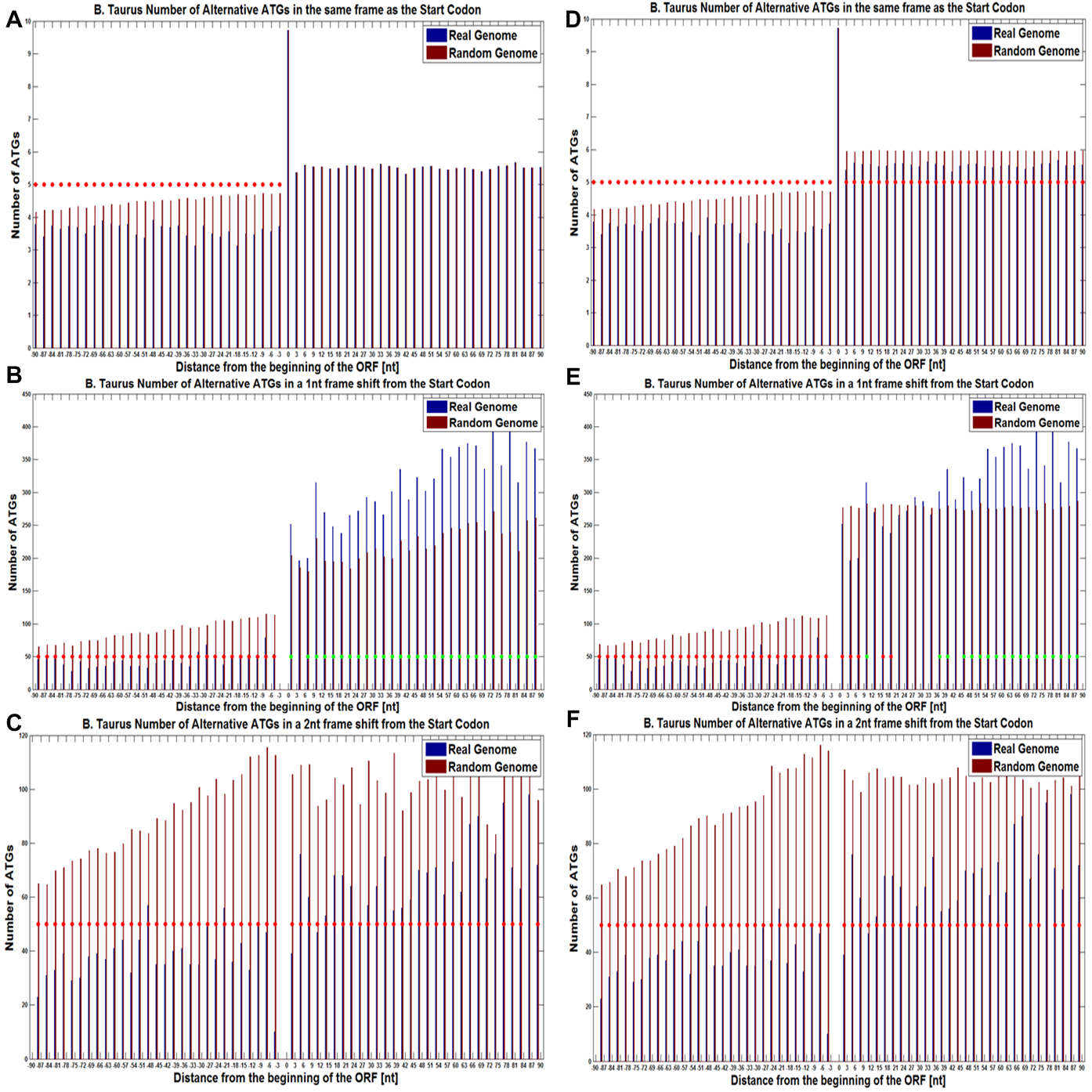

Supplement: Figure S26 — A–C. B. Taurus comparison of the genomic profiles of number of ATGs in the three frames to the ones obtained for randomized genomes with the same proteins, GC content, and codon bias (Methods). D–F. B. Taurus comparison of the genomic profiles of number of ATGs in the three frames to the ones obtained for randomized genomes that were generated by permuting the codons of each gene (Methods). (TIF) [file pcbi.1003136.s026.tif]

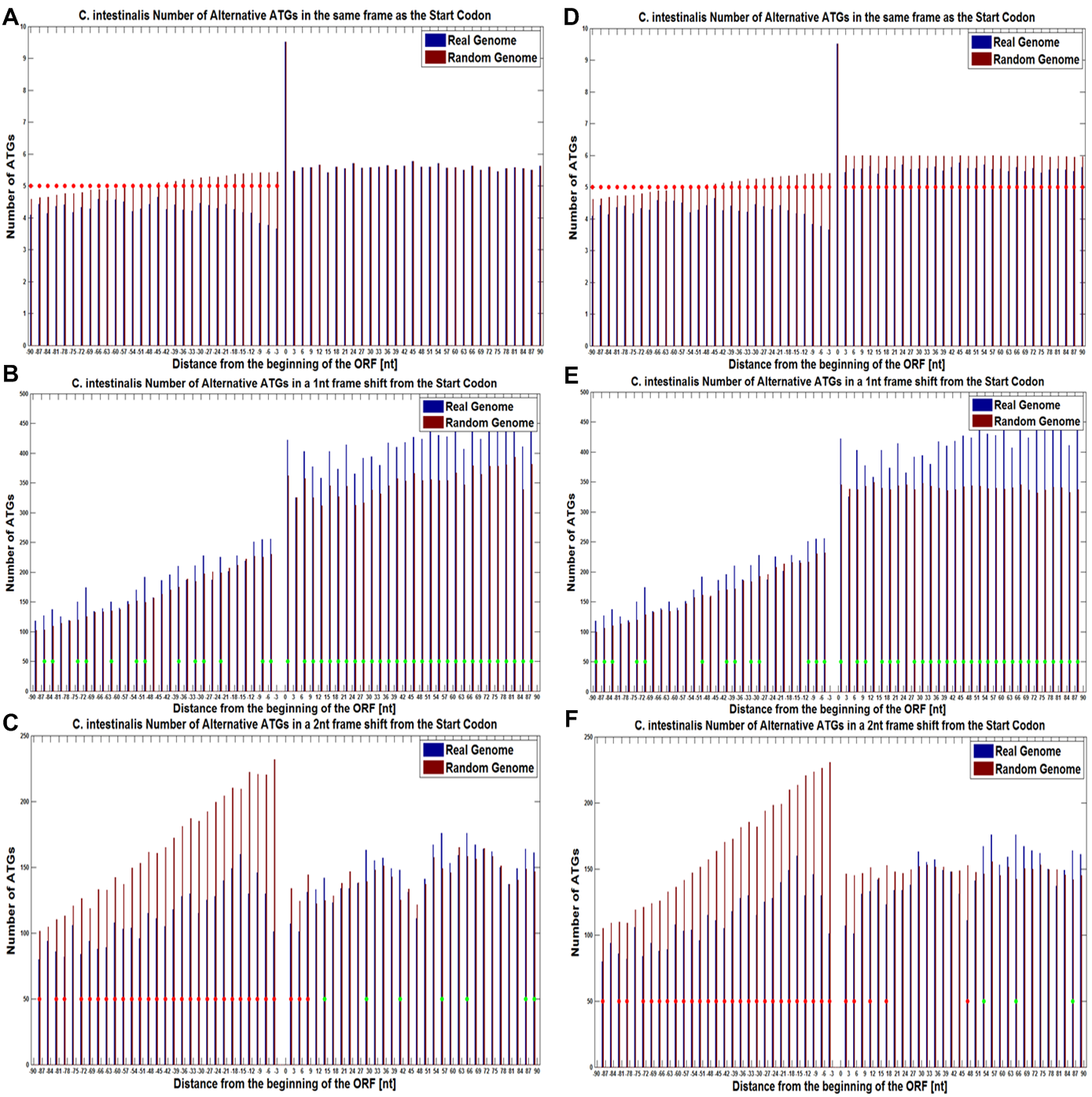

Supplement: Figure S27 — A–C. C. intestinalis comparison of the genomic profiles of number of ATGs in the three frames to the ones obtained for randomized genomes with the same proteins, GC content, and codon bias (Methods). D–F. C. intestinalis comparison of the genomic profiles of number of ATGs in the three frames to the ones obtained for randomized genomes that were generated by permuting the codons of each gene (Methods). (TIF) [file pcbi.1003136.s027.tif]

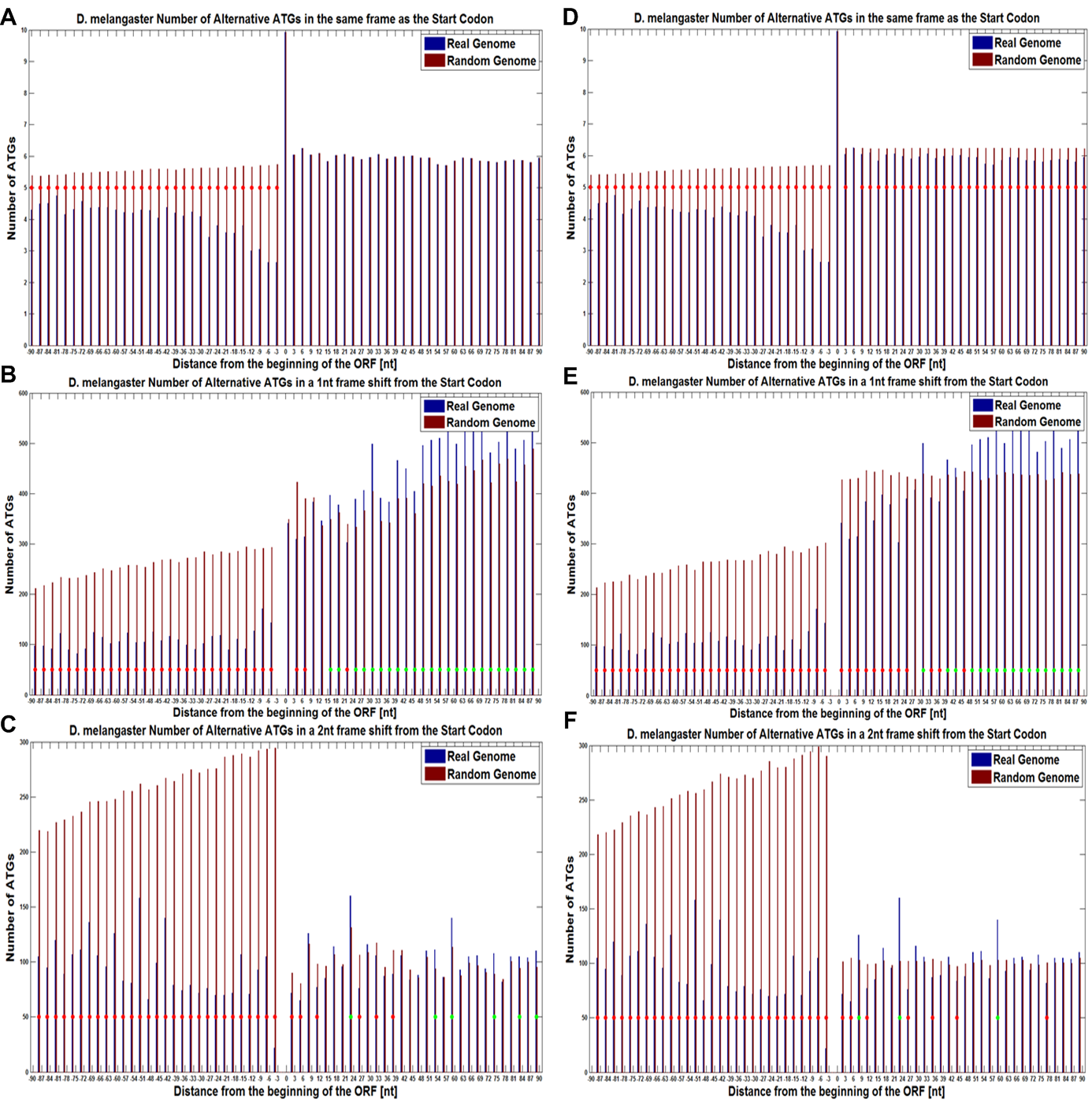

Supplement: Figure S28 — A–C. D. melangaster comparison of the genomic profiles of number of ATGs in the three frames to the ones obtained for randomized genomes with the same proteins, GC content, and codon bias (Methods). D–F. D. melangaster comparison of the genomic profiles of number of ATGs in the three frames to the ones obtained for randomized genomes that were generated by permuting the codons of each gene (Methods). (TIF) [file pcbi.1003136.s028.tif]

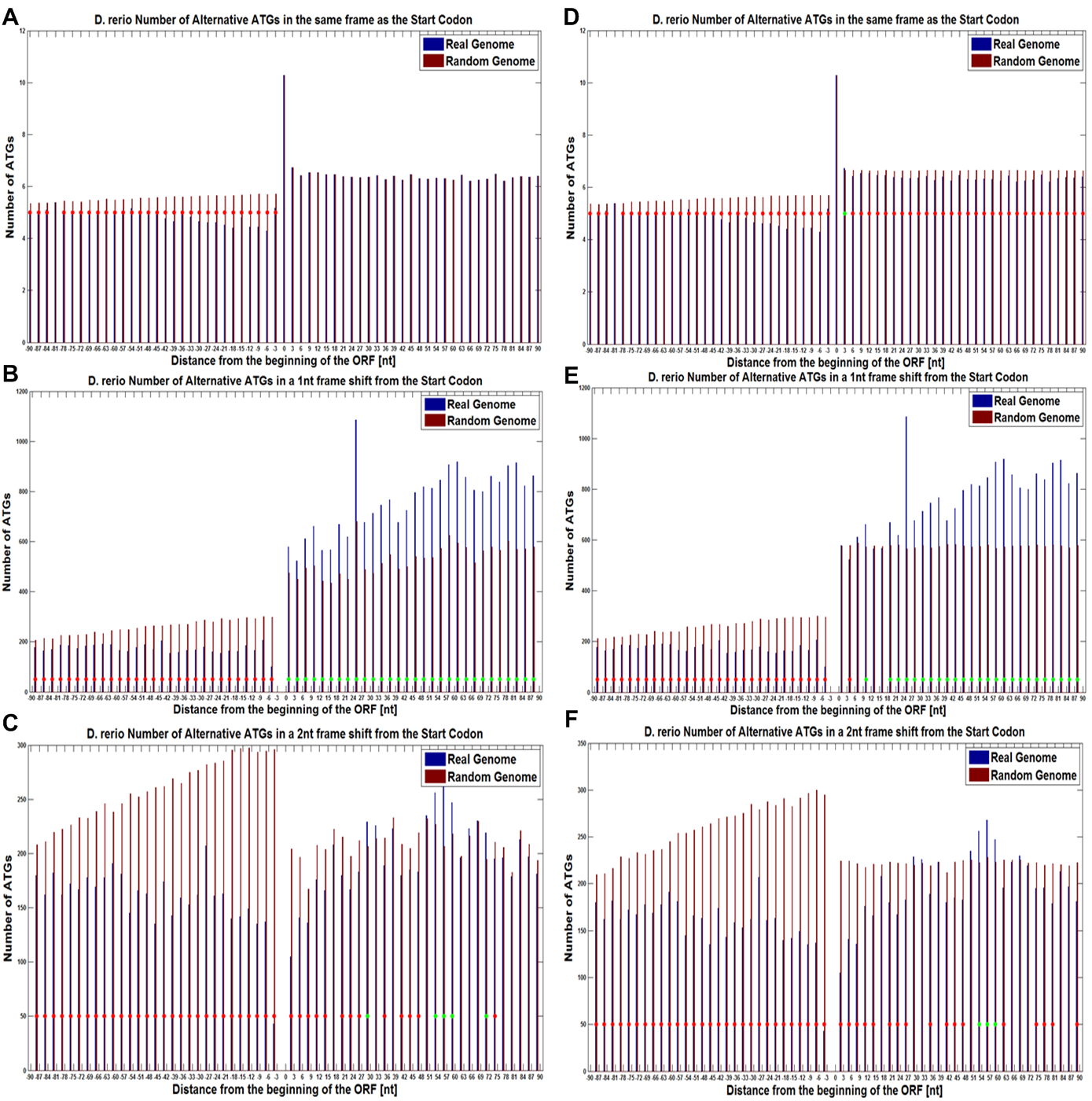

Supplement: Figure S29 — A–C. D. rerio comparison of the genomic profiles of number of ATGs in the three frames to the ones obtained for randomized genomes with the same proteins, GC content, and codon bias (Methods). D–F. D. rerio comparison of the genomic profiles of number of ATGs in the three frames to the ones obtained for randomized genomes that were generated by permuting the codons of each gene (Methods). (TIF) [file pcbi.1003136.s029.tif]

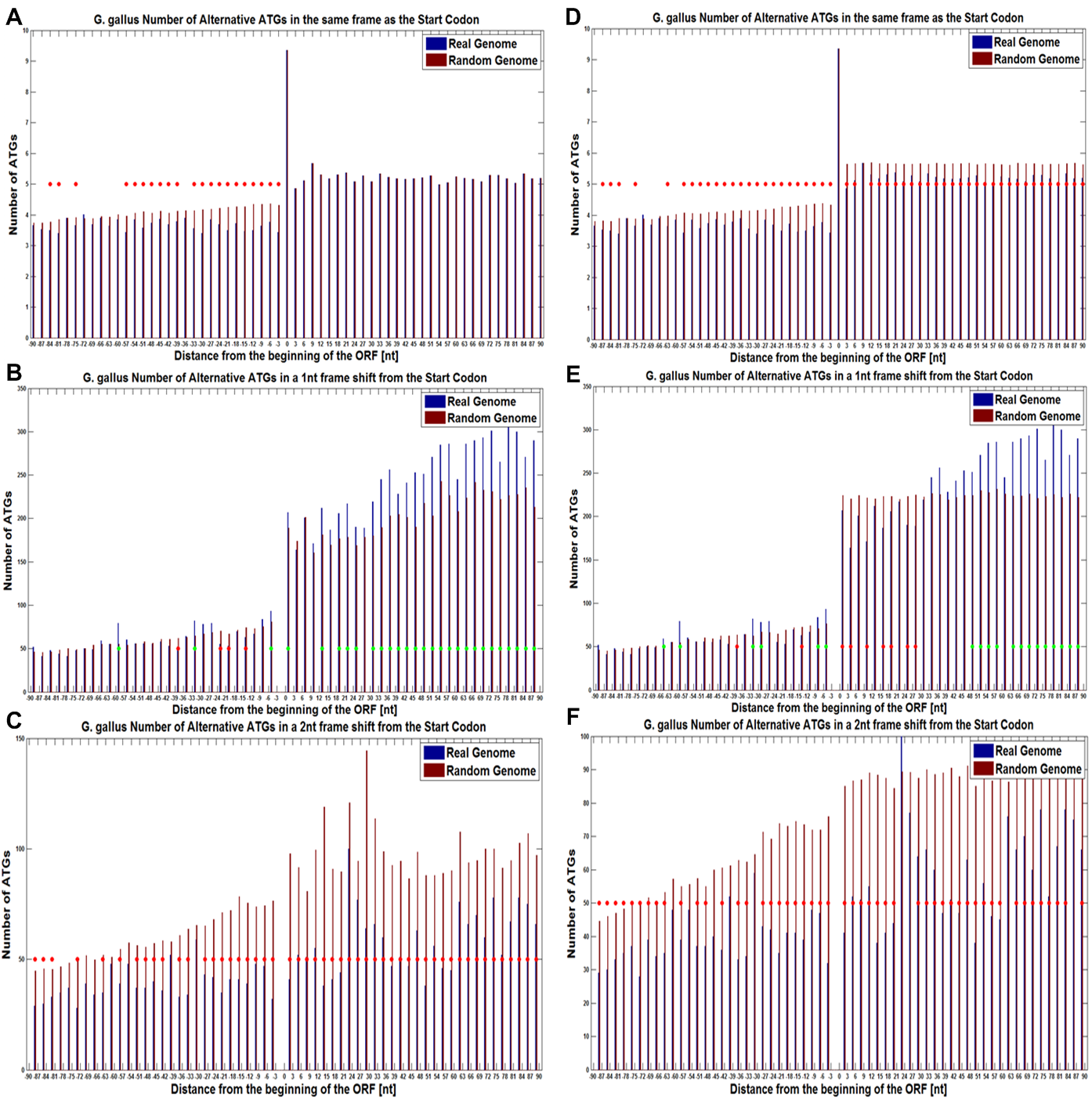

Supplement: Figure S30 — A–C. G. gallus comparison of the genomic profiles of number of ATGs in the three frames to the ones obtained for randomized genomes with the same proteins, GC content, and codon bias (Methods). D–F. G. gallus comparison of the genomic profiles of number of ATGs in the three frames to the ones obtained for randomized genomes that were generated by permuting the codons of each gene (Methods). (TIF) [file pcbi.1003136.s030.tif]

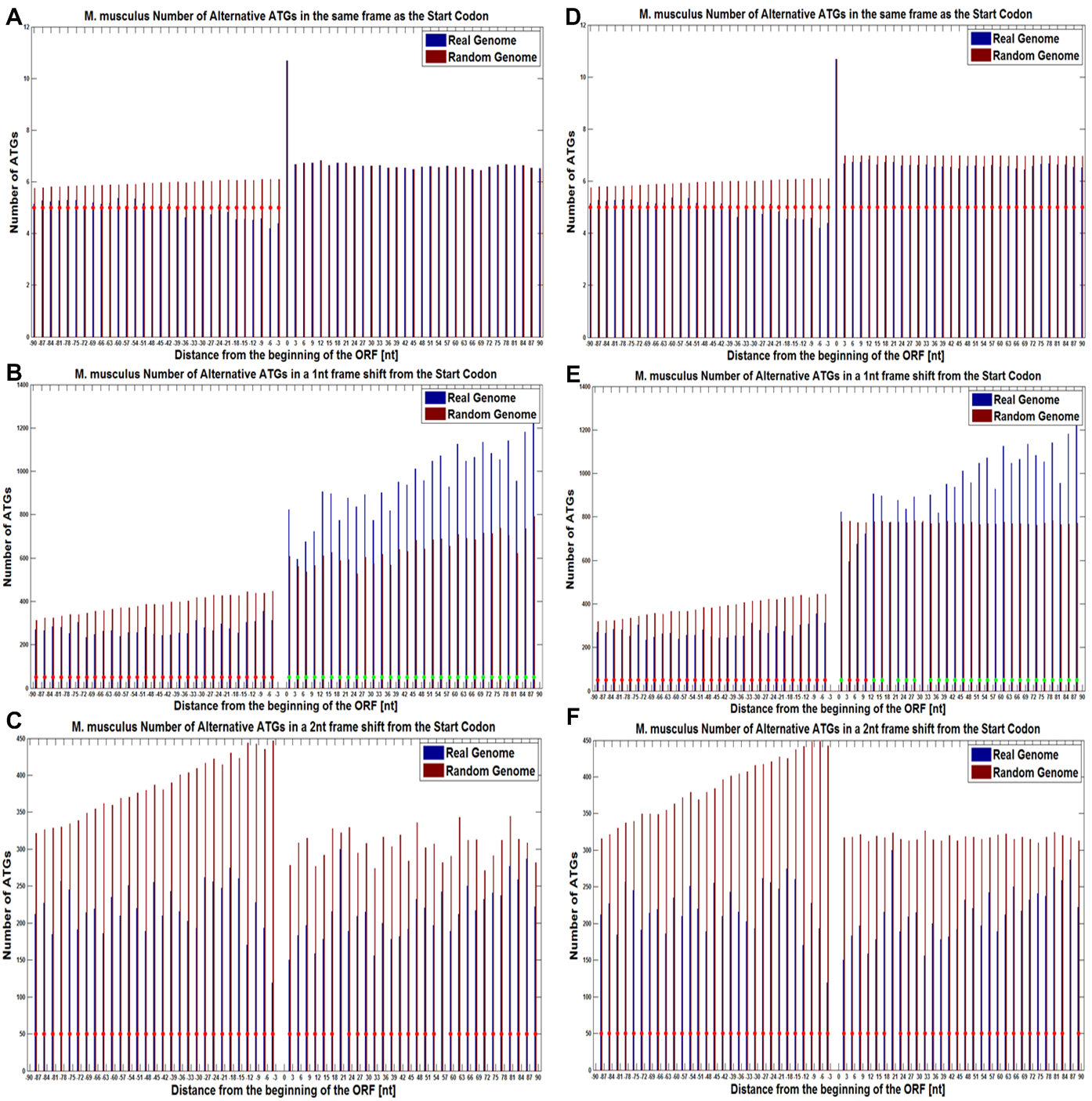

Supplement: Figure S31 — A–C. M. musculus comparison of the genomic profiles of number of ATGs in the three frames to the ones obtained for randomized genomes with the same proteins, GC content, and codon bias (Methods). D–F. M. musculus comparison of the genomic profiles of number of ATGs in the three frames to the ones obtained for randomized genomes that were generated by permuting the codons of each gene (Methods). (TIF) [file pcbi.1003136.s031.tif]

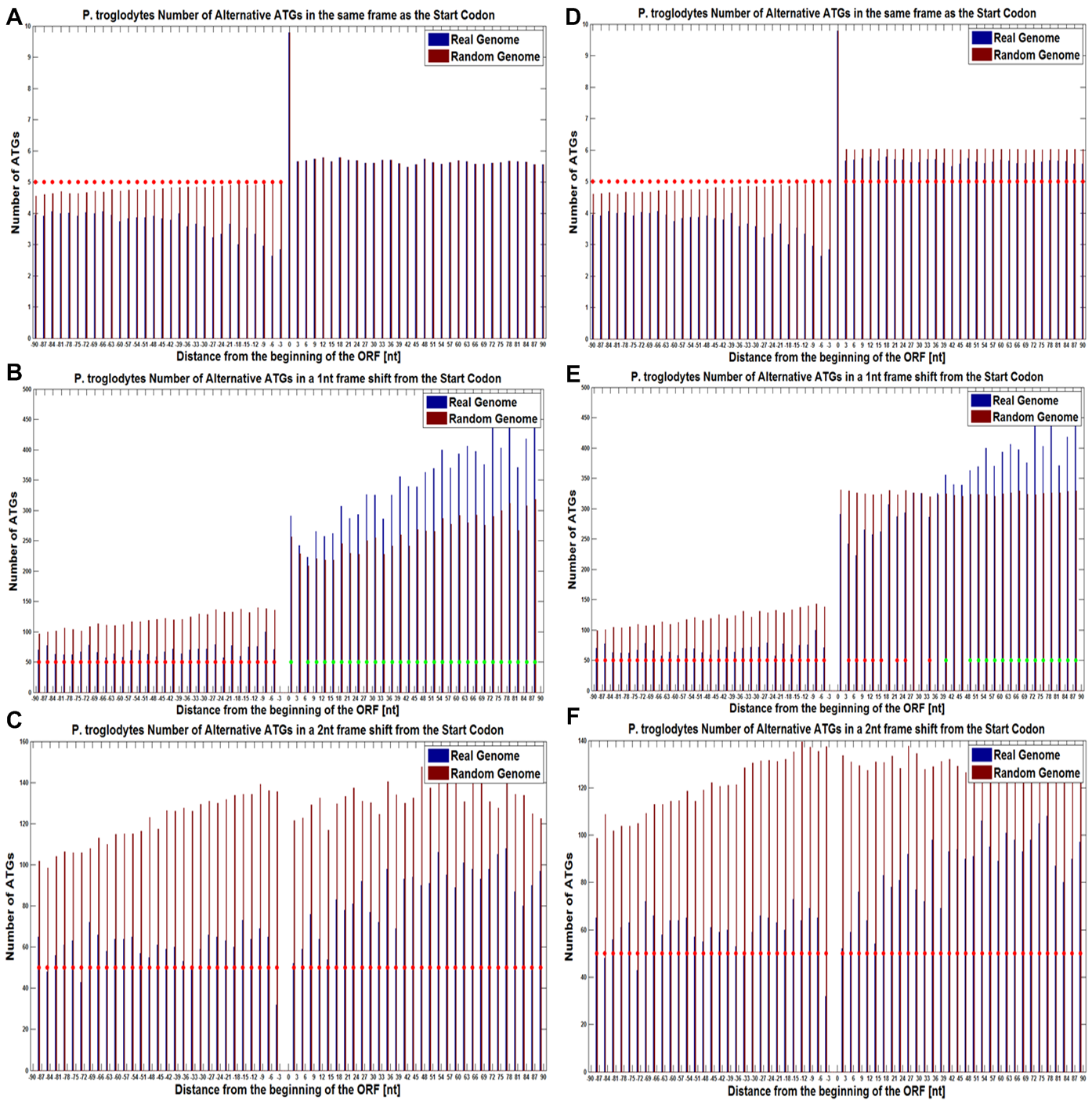

Supplement: Figure S32 — A–C. P. troglodytes comparison of the genomic profiles of number of ATGs in the three frames to the ones obtained for randomized genomes with the same proteins, GC content, and codon bias (Methods). D–F. P. troglodytes comparison of the genomic profiles of number of ATGs in the three frames to the ones obtained for randomized genomes that were generated by permuting the codons of each gene (Methods). (TIF) [file pcbi.1003136.s032.tif]

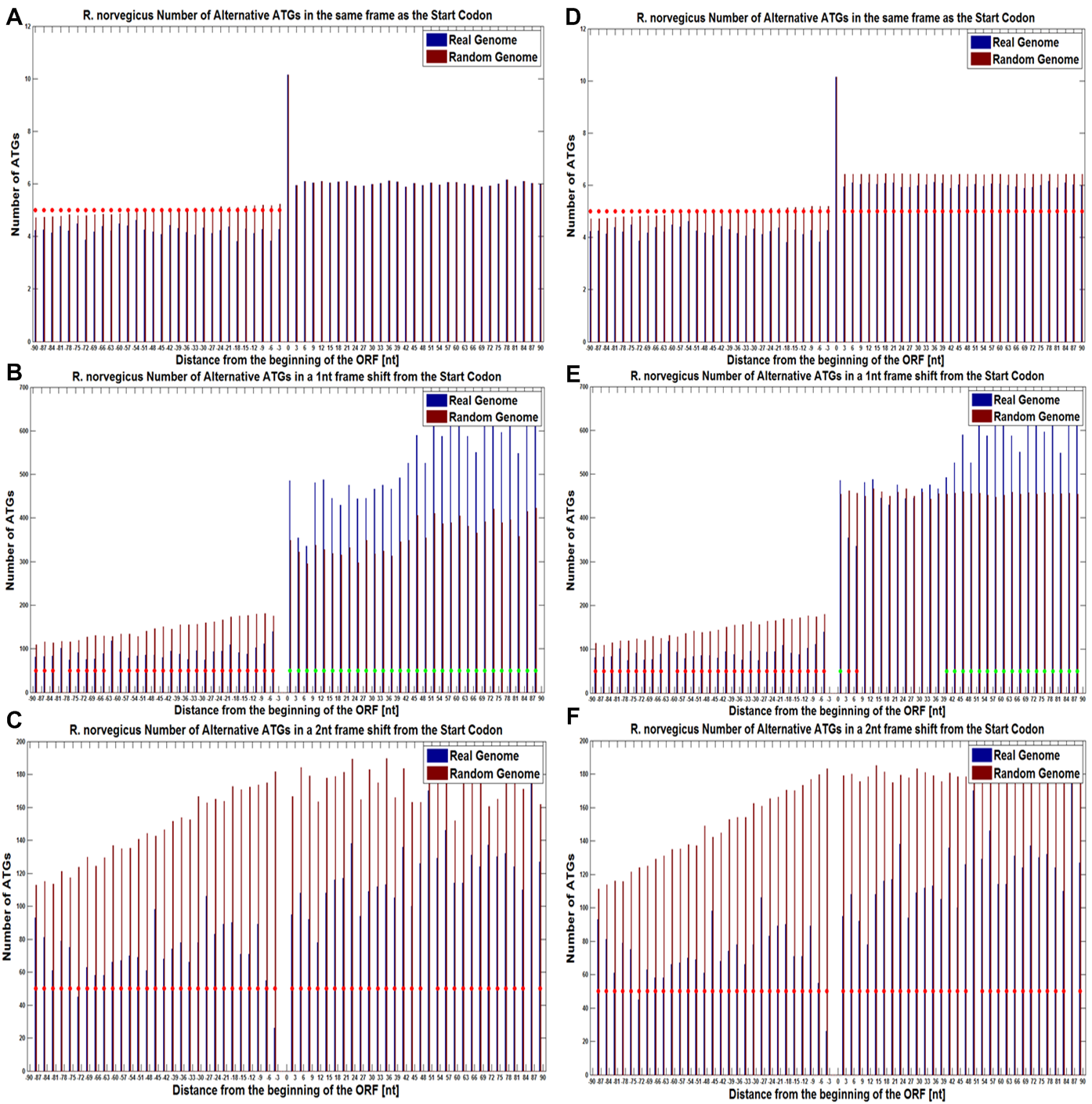

Supplement: Figure S33 — A–C. R. norvegicus comparison of the genomic profiles of number of ATGs in the three frames to the ones obtained for randomized genomes with the same proteins, GC content, and codon bias (Methods). D–F. R. norvegicus comparison of the genomic profiles of number of ATGs in the three frames to the ones obtained for randomized genomes that were generated by permuting the codons of each gene (Methods). (TIF) [file pcbi.1003136.s033.tif]

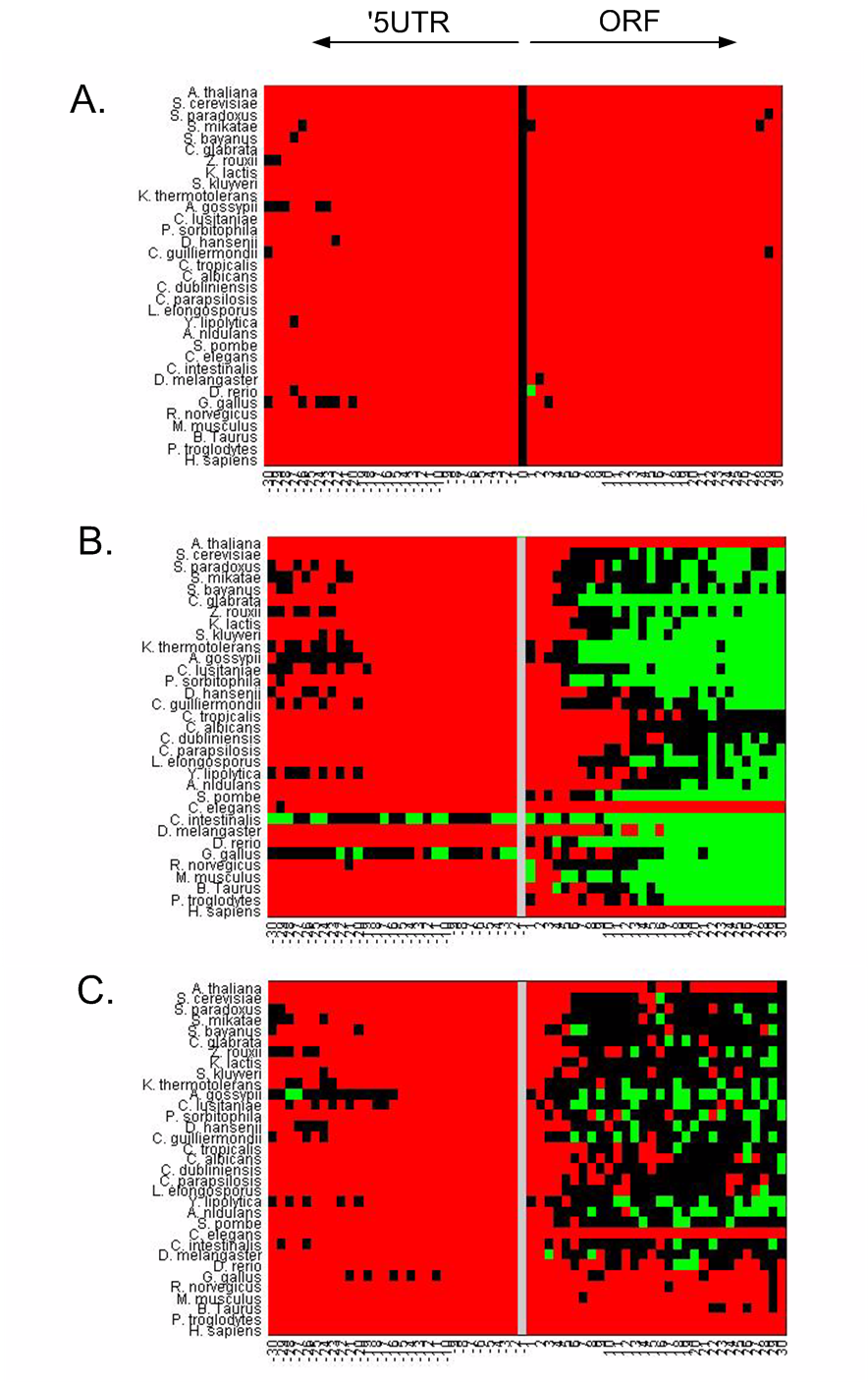

Supplement: Figure S34 — A–C. Genomic profiles of number of ATGs in the three frames of 33 eukaryotes' permuted randomized genomes' (Methods) first thirty codons upstream, and downstream the START ATG (A.- Frame 0 which is the reading frame of the protein, B. – Frame 1: 1 nt frame shift from the reading frame, C. – Frame 2: 2 nt frame shift from the reading frame). Each organism's number of ATGs were scaled according to the number of STDs distance from the mean, for the 5′UTR and ORF separately. As can be seen, in all frames, there is a universal signal of fewer ATGs near the beginning of the ORF. (TIF) [file pcbi.1003136.s034.tif]

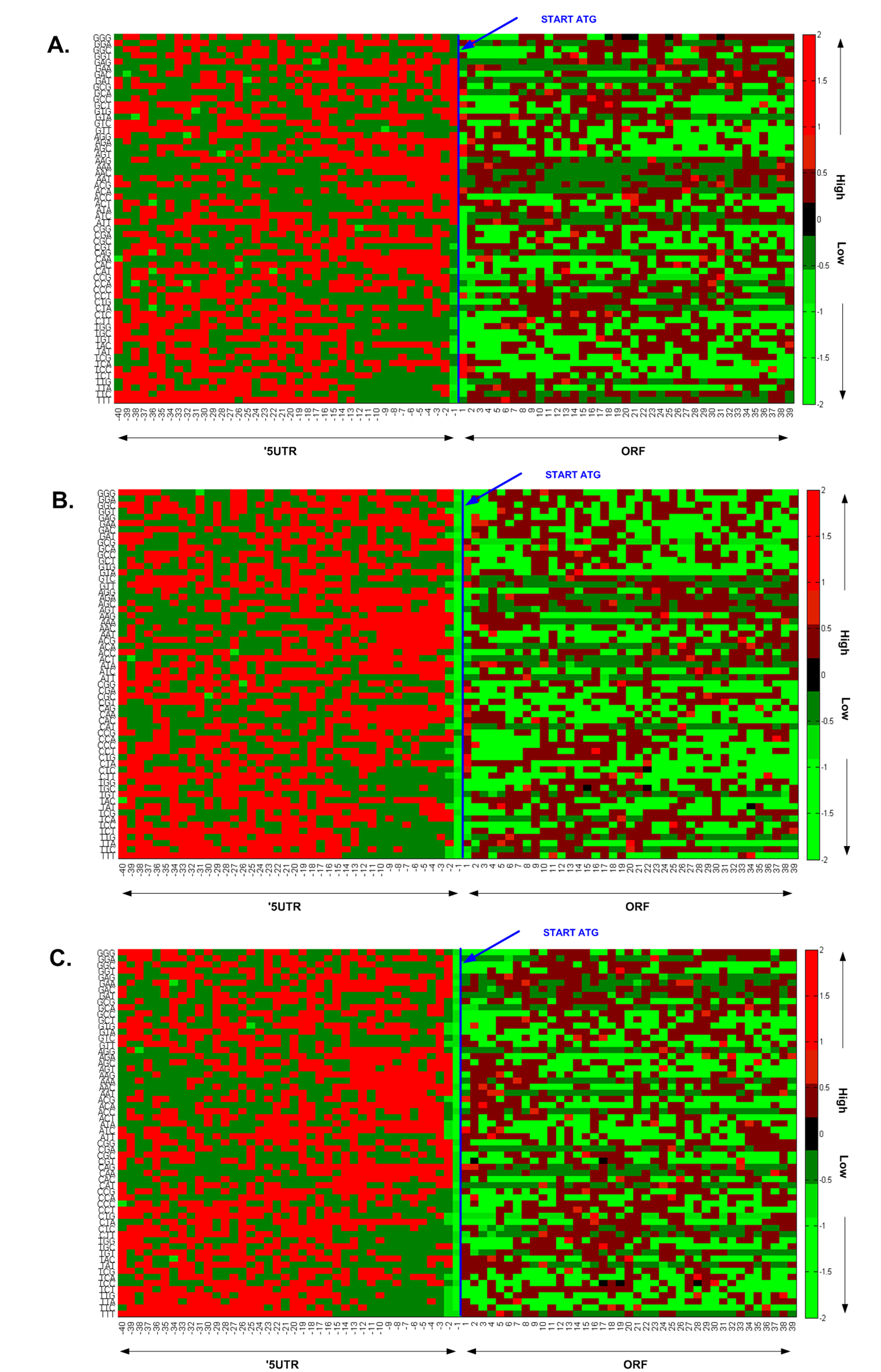

Supplement: Figure S35 — Genomic profiles of number of non-ATG codons in the three frames in S. cerevisiae's first forty codons upstream, and downstream the START ATG (A. Frame 0, B. Frame 1, C. Frame 2). For each organism, in each nucleotide position, the number of appearances of a codon was scaled according to the distance (in terms of the number of STDs, after normalizing by the maximal possible number of appearances of the codon in the position) from the mean number of appearances of the codon across all the positions, for the 5′UTR and ORF separately. A position with relatively more appearances of a codon (positive number of STDs from the mean) was marked in red (more appearances corresponds to a more reddish color), while positions with relatively less appearances of a codon (negative number of STDs from the mean) were marked in green (less appearances corresponds to a more greenish color). For each codon, for both the 5′UTR and the non-main frame shifts of the ORF, the length of the region with significantly lower number of appearances of the codon (Figure 4) was zero (i.e. there is no such region); this result demonstrates that the ATG codon behaves differently from non-ATG codons, supporting the hypothesis that the ATG depletion is related to translation initiation from the alternative ATG codons. (TIF) [file pcbi.1003136.s035.tif]

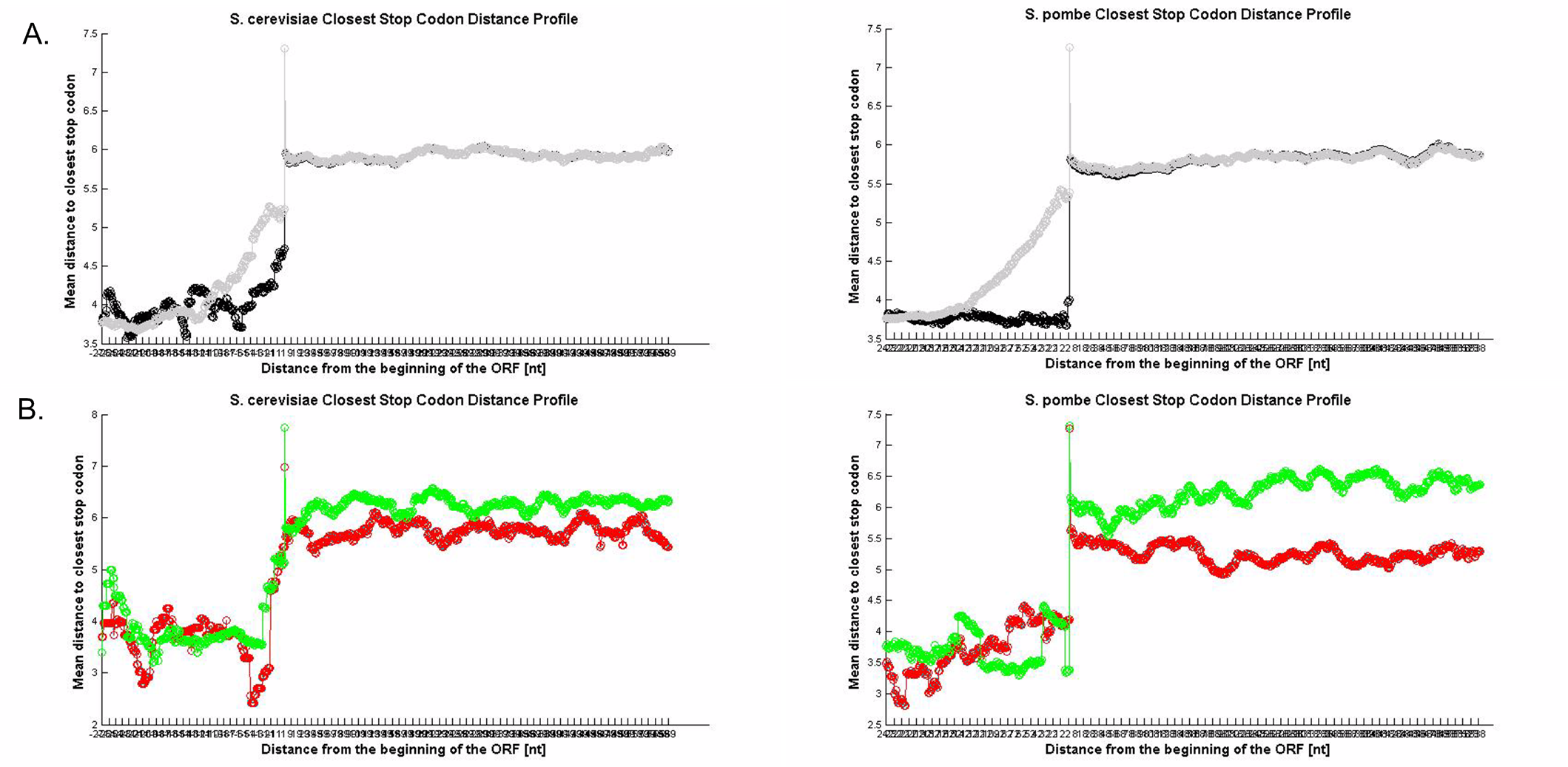

Supplement: Figure S36 — A. Distance to closest stop codon from alternative ATGs for real (black) and randomized (gray) genomes of S. cerevisiae and S. pombe. B. Distance to closest stop codon from alternative ATGs for highly (red) and lowly (green) expressed genes in terms of their (ribosomal density)·(mRNA levels) for S. cerevisiae and S. pombe (top/bottom 10%). (TIF) [file pcbi.1003136.s036.tif]

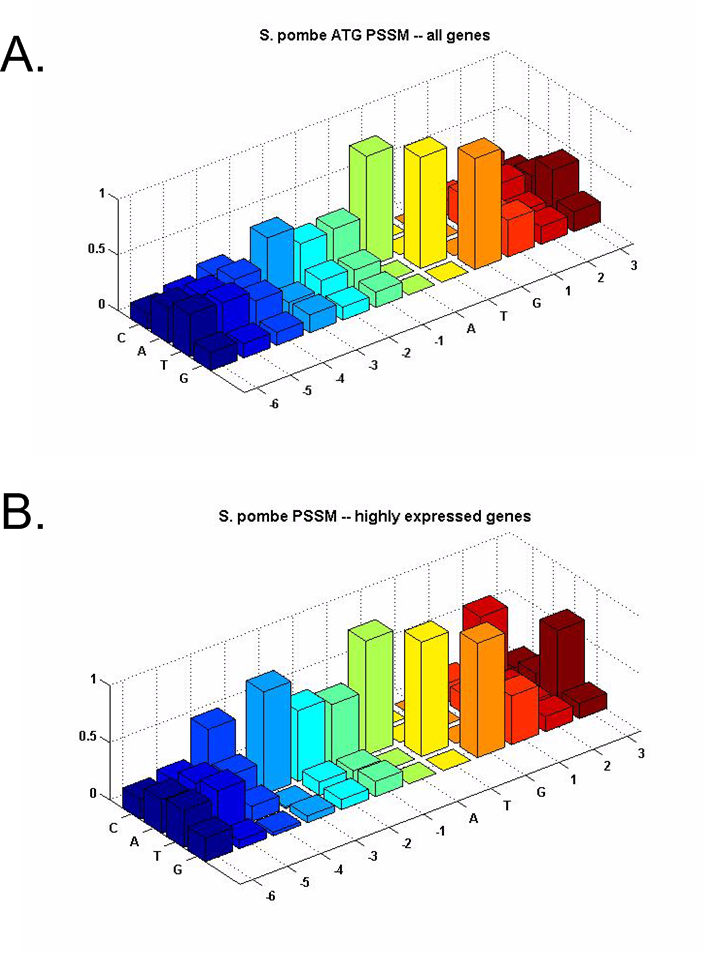

Supplement: Figure S37 — The PSSM describing the distribution of nucleotides near the main START ATG in S. pombe for all genes (A.), and for genes with high ribosomal-load (B.). (TIF) [file pcbi.1003136.s037.tif]
